# Supplementary material for: Nuclear bodies protect phase separated proteins from degradation in stressed proteome
Source: bioRxiv. 2023 Apr 21:2023.04.19.537522. Preprint. [Version 1] doi: 10.1101/2023.04.19.537522 (PMC10153235; doi:10.1101/2023.04.19.537522)

bioRxiv preprint doi: https://doi.org/10.1101/2023.04.19.537522; this version posted April 21, 2023. The copyright holder for this preprint (which was not certified by peer review) is the author/funder, who has granted bioRxiv a license to display the preprint in perpetuity. It is made available under aCC-BY 4.0 International license.

Supplementary Figures

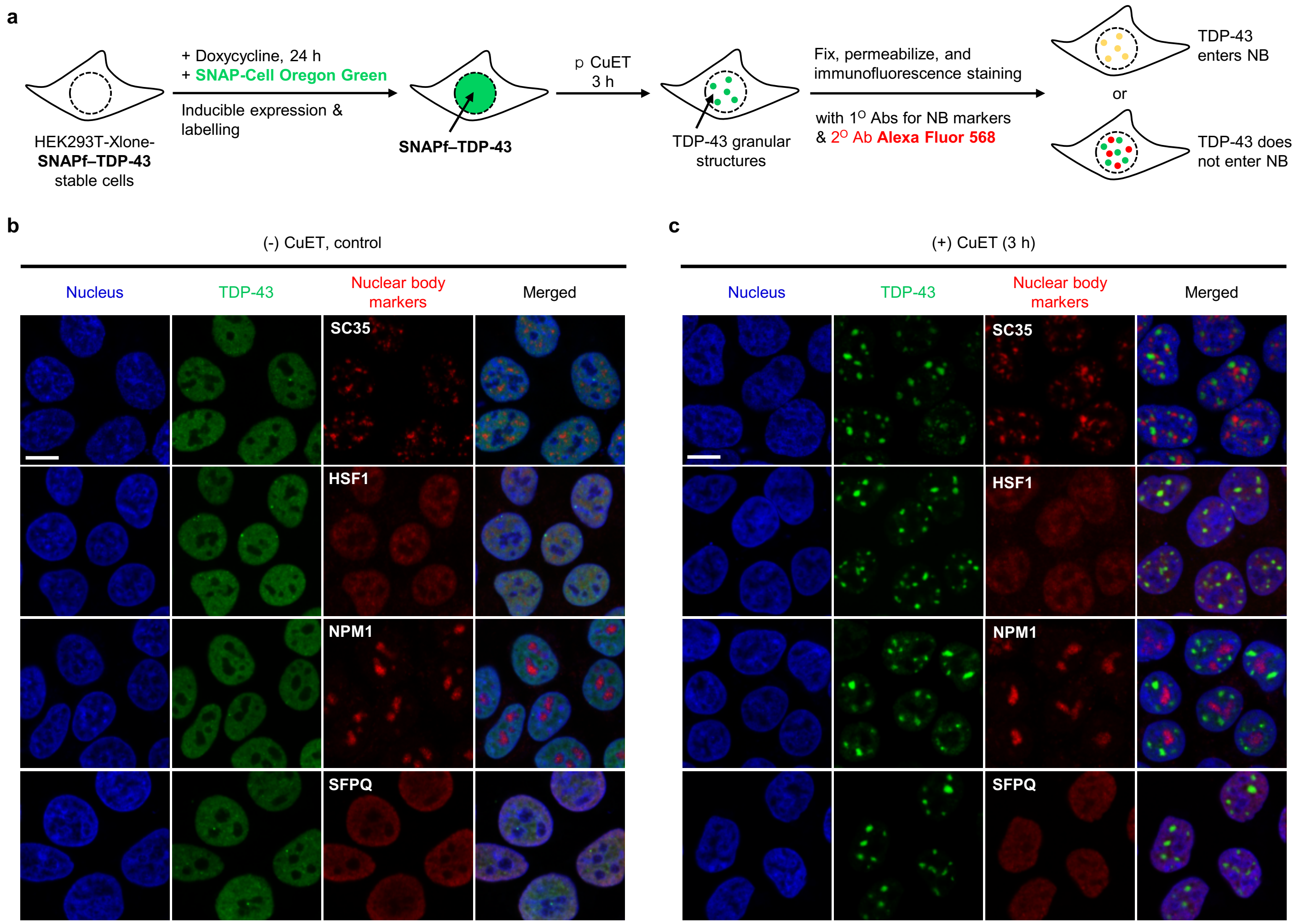

Figure S1. Immunofluorescence experiments show that TDP-43 does not enter other nuclear bodies, including nuclear speckles, nuclear stress bodies, nucleoli, and paraspeckles, upon treatment with CuET. (**a**) Schematic illustration of the procedure for the immunofluorescence experiments with anti-nuclear body makers (red) for cells stably expressing SNAPf–TDP-43 (green). SNAPf–TDP-43 stable cells were treated with doxycycline (140 ng/mL) and SNAP-Cell Oregon Green (0.5 µM) for 24 hours for the inducible expression and labeling of SNAPf–TDP-43, respectively. After the cells were further treated with CuET (5 µM) for 3 hours, they were fixed, permeabilized, and stained with the primary antibodies for several different nuclear body markers, and the secondary antibody conjugated with red fluorophore. (**b**), (**c**) Immunofluorescence imaging with anti-SC35 (for nuclear speckles), anti-HSF1 (for nuclear stress bodies), anti-NPM1 (for nucleoli), or anti-SFPQ (for paraspeckles) for cells stably expressing SNAPf-TDP-43 (labeled by SNAP-Cell Oregon Green, green) (b) without CuET treatment, or (c) upon treatment with CuET for 3 hours. Scale bar = 10 µm.

bioRxiv preprint doi: https://doi.org/10.1101/2023.04.19.537522; this version posted April 21, 2023. The copyright holder for this preprint (which was not certified by peer review) is the author/funder, who has granted bioRxiv a license to display the preprint in perpetuity. It is made available under aCC-BY 4.0 International license.

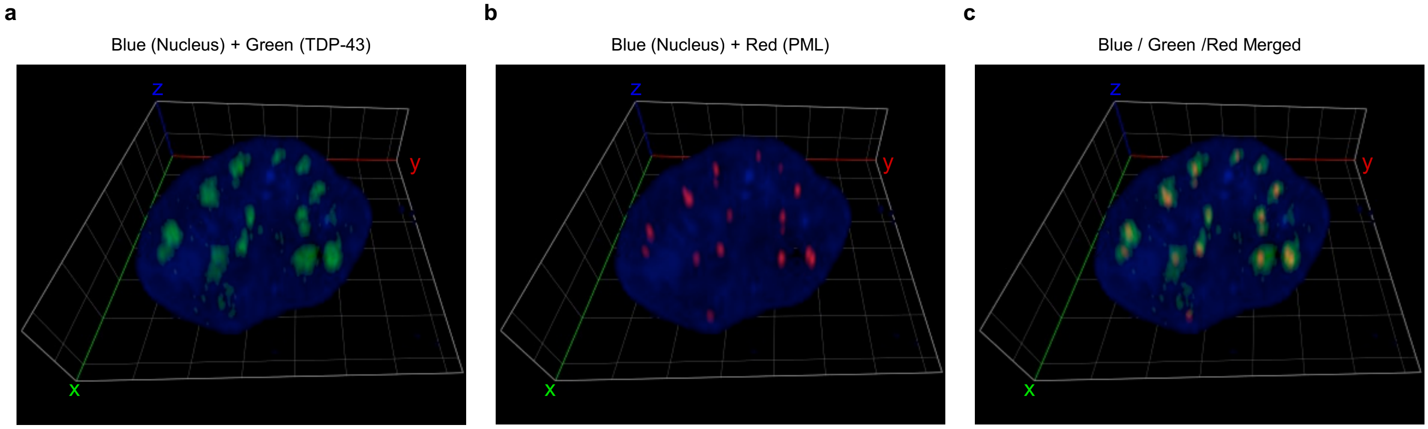

Figure S2. Z-stack and Airyscan imaging experiments reveal that PML nuclear bodies reside in the core of the TDP-43 granular structures formed by CuET treatment. Three-dimensional z-stack reconstruction of Airyscan imaging for the cell stably expressing SNAPf–TDP-43 (labeled by SNAP-Cell Oregon Green, green) upon treatment with CuET for 1 h. PML nuclear bodies were labeled with anti-PML (red) by immunofluorescence. (**a**) Merged image of blue (DAPI) and green (TDP-43). (**b**) Merged image of blue (DAPI) and red (PML). (**c**) Merged image of blue (DAPI), green (TDP-43) and red (PML). The unit of the grid is 3 μm.

bioRxiv preprint doi: https://doi.org/10.1101/2023.04.19.537522; this version posted April 21, 2023. The copyright holder for this preprint (which was not certified by peer review) is the author/funder, who has granted bioRxiv a license to display the preprint in perpetuity. It is made available under aCC-BY 4.0 International license.

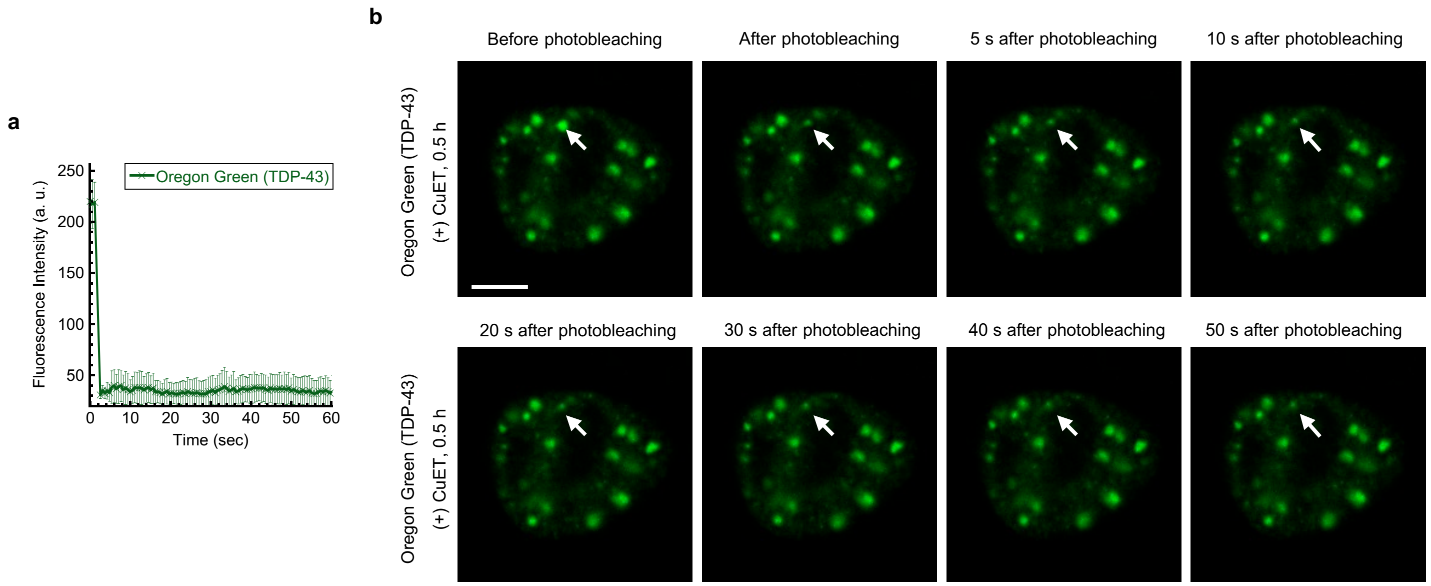

Figure S3. Fluorescence recovery after photobleaching experiments indicate that TDP-43 does not exhibit dynamic properties in the granular structures formed by CuET treatment. (**a**) Time-course of the fluorescence intensity profile at regions of interest from Oregon Green (TDP-43) channel in cells stably expressing SNAPf–TDP-43 (labeled by SNAP-Cell Oregon Green, green) upon treatment with CuET for 0.5 h. The error bar represents standard deviation from three independent measurements at each respective time point. (**b**) Representative time-lapse images of cells stably expressing SNAPf–TDP-43 (labeled by SNAP-Cell Oregon Green, green) with CuET treatment before and after photobleaching. The white arrow indicates the region of interest for the FRAP analysis. Scale bar = 5 µm.

bioRxiv preprint doi: https://doi.org/10.1101/2023.04.19.537522; this version posted April 21, 2023. The copyright holder for this preprint (which was not certified by peer review) is the author/funder, who has granted bioRxiv a license to display the preprint in perpetuity. It is made available under aCC-BY 4.0 International license.

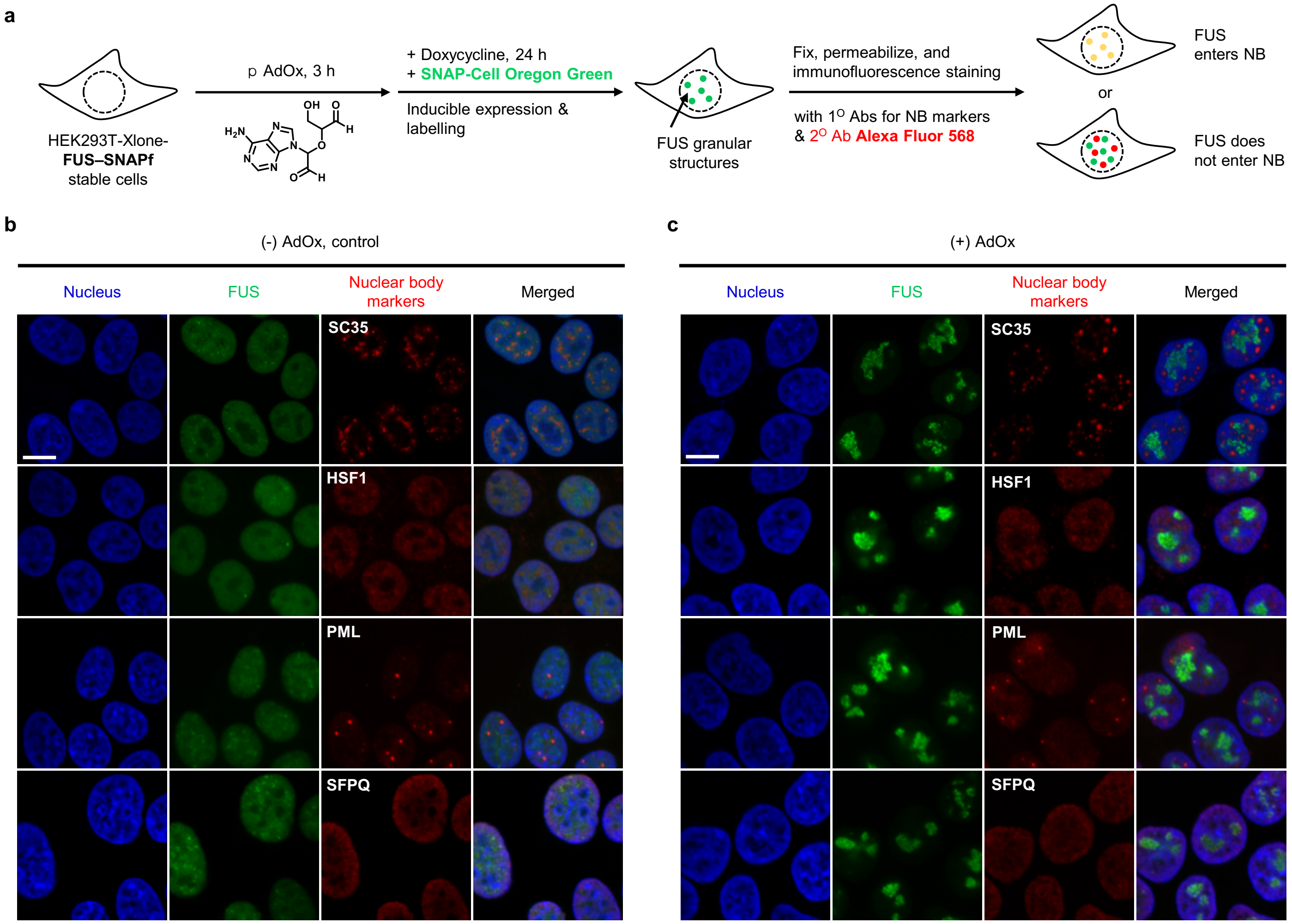

Figure S4. Immunofluorescence experiments show that FUS does not enter other nuclear bodies, including nuclear speckles, nuclear stress bodies, PML nuclear bodies, and paraspeckles, upon pretreatment with AdOx. (**a**) Schematic illustration of the procedure for the immunofluorescence experiments with anti-nuclear body makers (red) for cells stably expressing FUS–SNAPf (green). FUS–SNAPf stable cells were pretreated with AdOx (25 μM) or not 3 hours before they were further treated with doxycycline (140 ng/mL) and SNAP-Cell Oregon Green (0.5 μM) for 24 hours for the inducible expression and labeling of FUS–SNAPf, respectively. The cells were then fixed, permeabilized, and stained with the primary antibodies for several different nuclear body markers, and the secondary antibody conjugated with red fluorophore. (**b**), (**c**) Immunofluorescence imaging with anti-SC35 (for nuclear speckles), anti-HSF1 (for nuclear stress bodies), anti-PML (for the PML nuclear bodies), or anti-SFPQ (for paraspeckles) for cells stably expressing FUS–SNAPf (labeled by SNAP-Cell Oregon Green, green) (b) without AdOx pretreatment, or (c) with AdOx pretreatment. Scale bar = 10 μm.

bioRxiv preprint doi: https://doi.org/10.1101/2023.04.19.537522; this version posted April 21, 2023. The copyright holder for this preprint (which was not certified by peer review) is the author/funder, who has granted bioRxiv a license to display the preprint in perpetuity. It is made available under aCC-BY 4.0 International license.

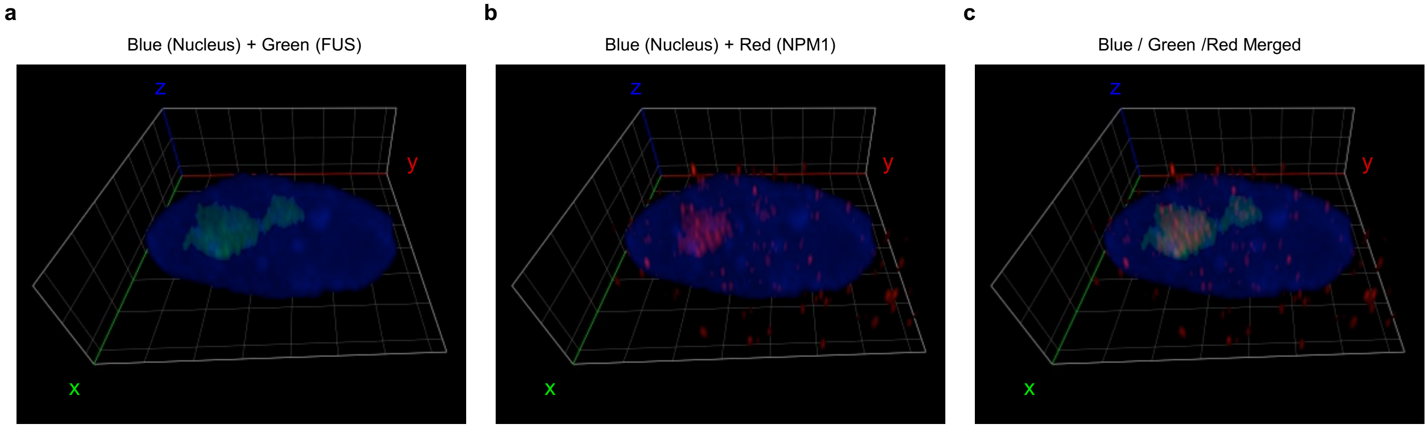

Figure S5. Z-stack and Airyscan imaging experiments reveal that FUS resides within the nucleolus when pretreated with AdOx. Three-dimensional z-stack reconstruction of Airyscan imaging for the cell stably expressing FUS–SNAPf (labeled by SNAP-Cell Oregon Green, green) upon AdOx treatment. Nucleoli were labeled with anti-NPM1 (red) by immunofluorescence. (**a**) Merged image of blue (DAPI) and green (FUS). (**b**) Merged image of blue (DAPI) and red (NPM1). (**c**) Merged image of blue (DAPI), green (FUS) and red (NPM1). The unit of the grid is 3 μm.

bioRxiv preprint doi: https://doi.org/10.1101/2023.04.19.537522; this version posted April 21, 2023. The copyright holder for this preprint (which was not certified by peer review) is the author/funder, who has granted bioRxiv a license to display the preprint in perpetuity. It is made available under aCC-BY 4.0 International license.

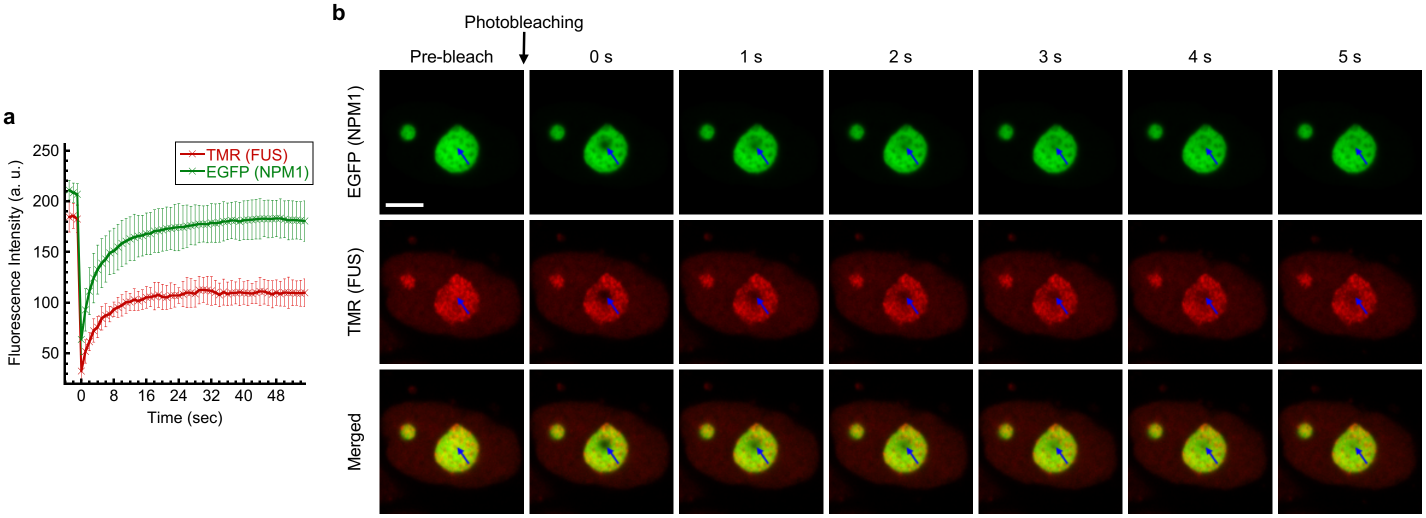

Figure S6. Fluorescence recovery after photobleaching experiments indicate that FUS remains diffuse in the nucleolus when pretreated with AdOx. (**a**) Time-course of the fluorescence intensity profiles at regions of interest from EGFP (NPM1) and TMR (FUS) channels in cells co-expressing EGFP–NPM1 (green) and FUS–SNAPf (labeled by SNAP-Cell TMR Star, red) with AdOx pretreatment. The error bar represents standard deviation from three independent measurements at each respective time point. (**b**) Representative time-lapse images of live cells co-expressing EGFP–NPM1 (green) and FUS–SNAPf (labeled by SNAP-Cell TMR Star, red) with AdOx pretreatment before and after photobleaching. The blue arrow indicates the region of interest for the FRAP analysis. Scale bar = 5 μm.

bioRxiv preprint doi: https://doi.org/10.1101/2023.04.19.537522; this version posted April 21, 2023. The copyright holder for this preprint (which was not certified by peer review) is the author/funder, who has granted bioRxiv a license to display the preprint in perpetuity. It is made available under aCC-BY 4.0 International license.

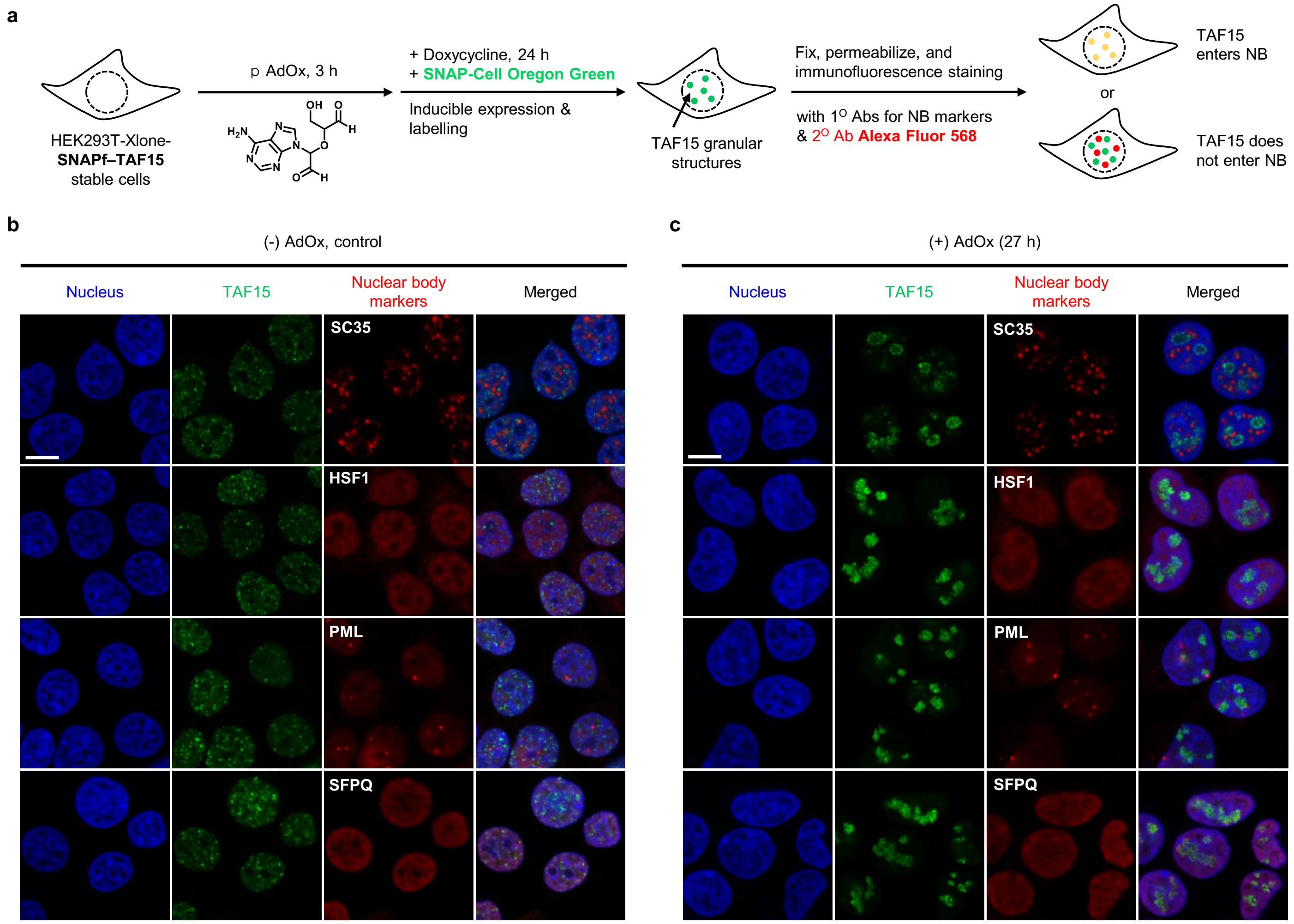

Figure S7. Immunofluorescence experiments show that TAF15 does not enter other nuclear bodies, including nuclear speckles, nuclear stress bodies, PML nuclear bodies, and paraspeckles, upon pretreatment with AdOx. (**a**) Schematic illustration of the general experimental procedure for the immunofluorescence imaging with anti-nuclear body makers (red) for cells stably expressing SNAPf–TAF15 (green). SNAPf–TAF15 stable cells were pretreated with AdOx (25 μM) or not 3 hours before they were further treated with doxycycline (140 ng/mL) and SNAP-Cell Oregon Green (0.5 μM) for 24 hours for the inducible expression and labeling of SNAPf–TAF15, respectively. The cells were then fixed, permeabilized, and stained with the primary antibodies for several different nuclear body markers, and the secondary antibody conjugated with red fluorophore. (**b**), (**c**) Immunofluorescence imaging with anti-SC35 (for nuclear speckles), anti-HSF1 (for nuclear stress bodies), anti-PML (for the PML nuclear bodies), or anti-SFPQ (for the paraspeckles) for cells stably expressing SNAPf–TAF15 (labeled by SNAP-Cell Oregon Green) (b) without AdOx pretreatment, or (c) with AdOx pretreatment. Scale bar = 10 μm.

bioRxiv preprint doi: https://doi.org/10.1101/2023.04.19.537522; this version posted April 21, 2023. The copyright holder for this preprint (which was not certified by peer review) is the author/funder, who has granted bioRxiv a license to display the preprint in perpetuity. It is made available under aCC-BY 4.0 International license.

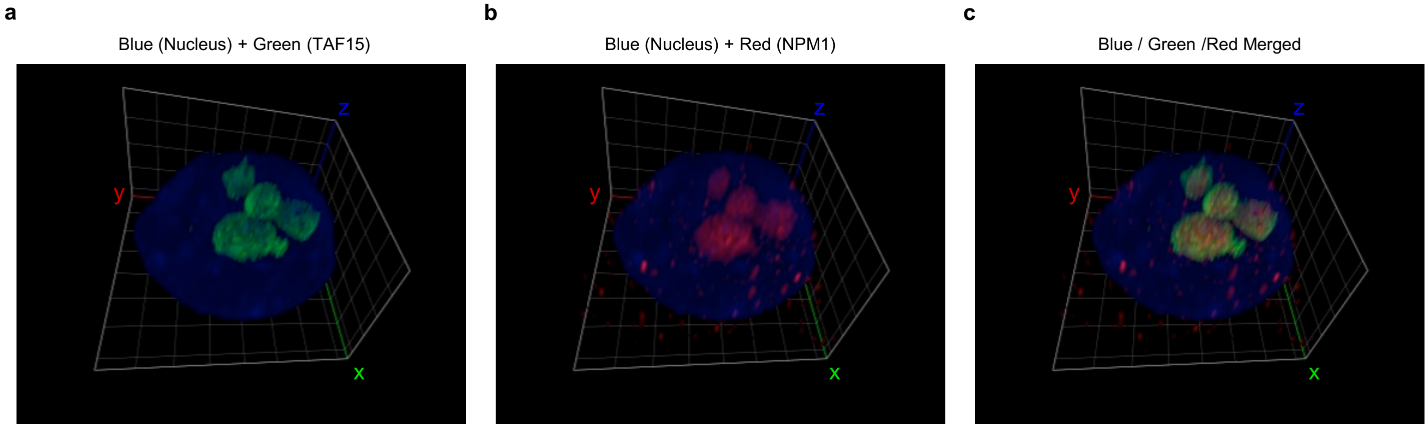

Figure S8. Z-stack and Airyscan imaging experiments reveal that TAF15 resides within the nucleolus when pretreated AdOx. Three-dimensional z-stack reconstruction of Airyscan imaging for the cell stably expressing SNAPf–TAF15 (labeled by SNAP-Cell Oregon Green, green) upon AdOx treatment. The nucleoli were labeled with anti-NPM1 (red) by immunofluorescence. (**a**) Merged image of blue (DAPI) and green (TAF15). (**b**) Merged image of blue (DAPI) and red (NPM1). (**c**) Merged image of blue (DAPI), green (TAF15) and red (NPM1). The unit of the grid is 3 μm.

bioRxiv preprint doi: https://doi.org/10.1101/2023.04.19.537522; this version posted April 21, 2023. The copyright holder for this preprint (which was not certified by peer review) is the author/funder, who has granted bioRxiv a license to display the preprint in perpetuity. It is made available under aCC-BY 4.0 International license.

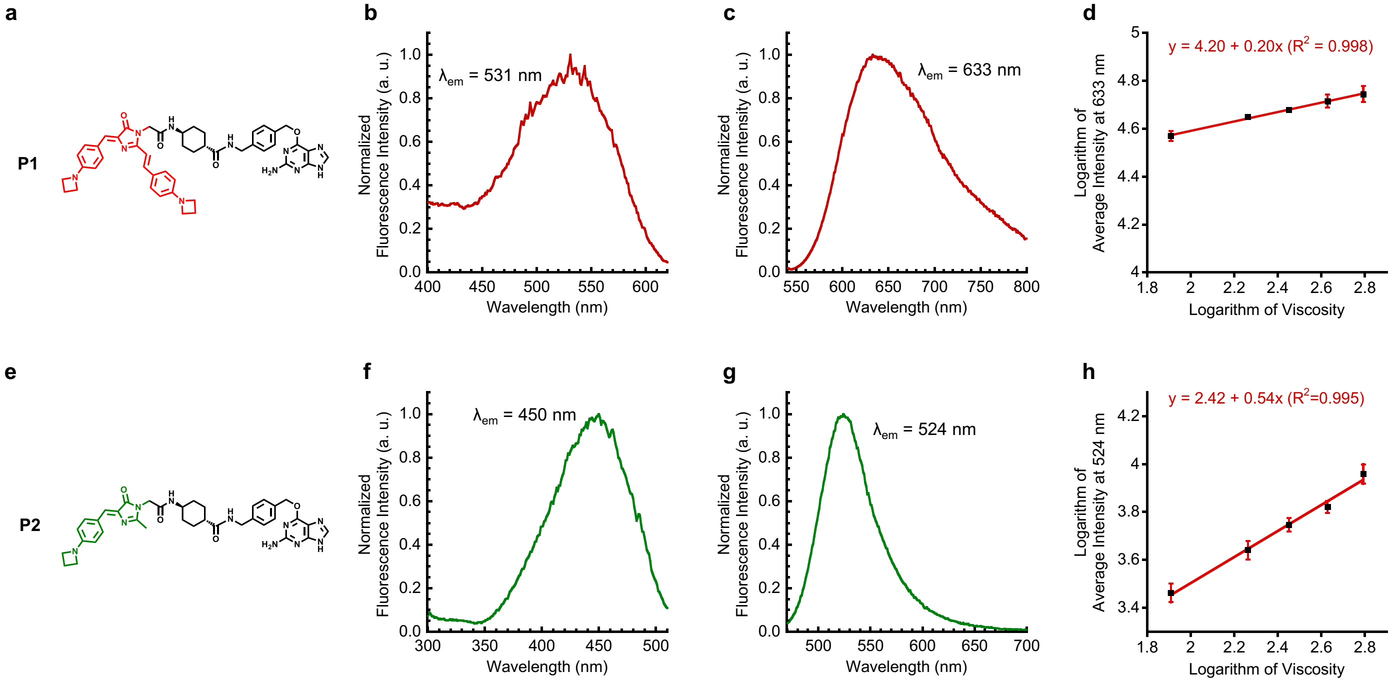

Figure S9. Photophysical characterization of P1 and P2 shows that they are spectrally orthogonal one another and have distinct viscosity sensitivity. (**a**) Structure of **P1**. (**b**) Fluorescence excitation spectrum of **P1** (5 μM) in glycerol. (**c**) Fluorescence emission spectrum of **P1** (5 μM) in glycerol. (**d**) The viscosity sensitivity of **P1** was determined to be 0.20 from the slope of the linear plot of the logarithm of the fluorescence emission intensity of **P1** at 633 nm as a function of logarithm of viscosity. (**e**) Structure of **P2**. (**f**) Fluorescence excitation spectrum of **P2** (5 μM) in glycerol. (**g**) Fluorescence emission spectrum of **P2** (5 μM) in glycerol. (**h**) The viscosity sensitivity of **P2** was determined to be 0.54 from the slope of the linear plot of the logarithm of the fluorescence emission intensity of **P2** at 524 nm as a function of logarithm of viscosity. All fluorescence was measured by using a Tecan infinite M1000Pro fluorescence microplate reader at room temperature.

bioRxiv preprint doi: https://doi.org/10.1101/2023.04.19.537522; this version posted April 21, 2023. The copyright holder for this preprint (which was not certified by peer review) is the author/funder, who has granted bioRxiv a license to display the preprint in perpetuity. It is made available under aCC-BY 4.0 International license.

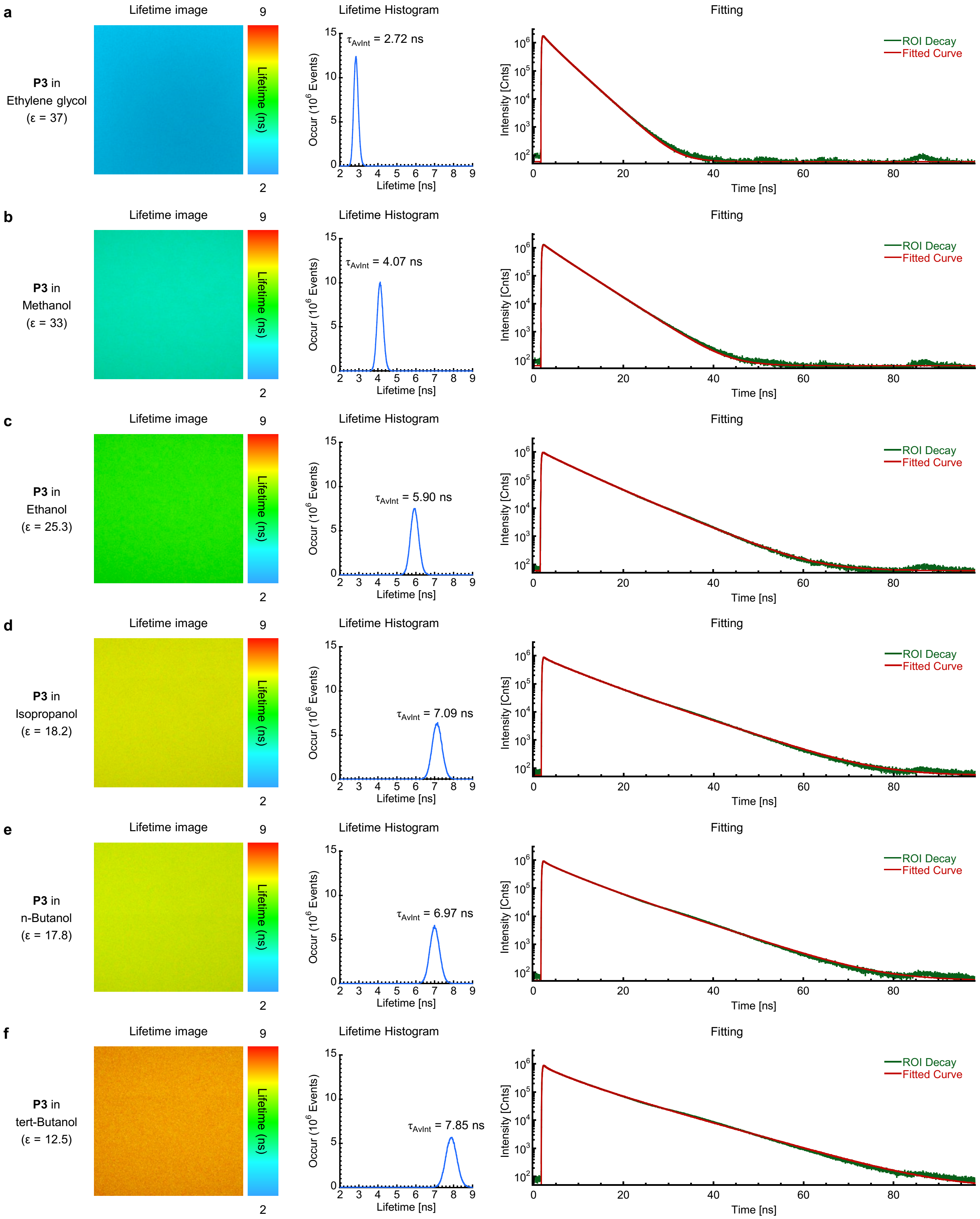

bioRxiv preprint doi: https://doi.org/10.1101/2023.04.19.537522; this version posted April 21, 2023. The copyright holder for this preprint (which was not certified by peer review) is the author/funder, who has granted bioRxiv a license to display the preprint in perpetuity. It is made available under aCC-BY 4.0 International license.

Figure S10. Average fluorescence lifetime (τ_{AvInt}) of P3 increases as the dielectric constant (ε) of solvent tested decreases. The fluorescence lifetime images (left panel), lifetime histograms (center panel), and lifetime decay curves (right panel) of **P3** in various polar protic solvents with different dielectric constant values. (**a**) in ethylene glycol, (**b**) in methanol, (**c**) in ethanol, (**d**) isopropanol, (**e**) n-butanol, (**f**) tert-butanol.

bioRxiv preprint doi: https://doi.org/10.1101/2023.04.19.537522; this version posted April 21, 2023. The copyright holder for this preprint (which was not certified by peer review) is the author/funder, who has granted bioRxiv a license to display the preprint in perpetuity. It is made available under aCC-BY 4.0 International license.

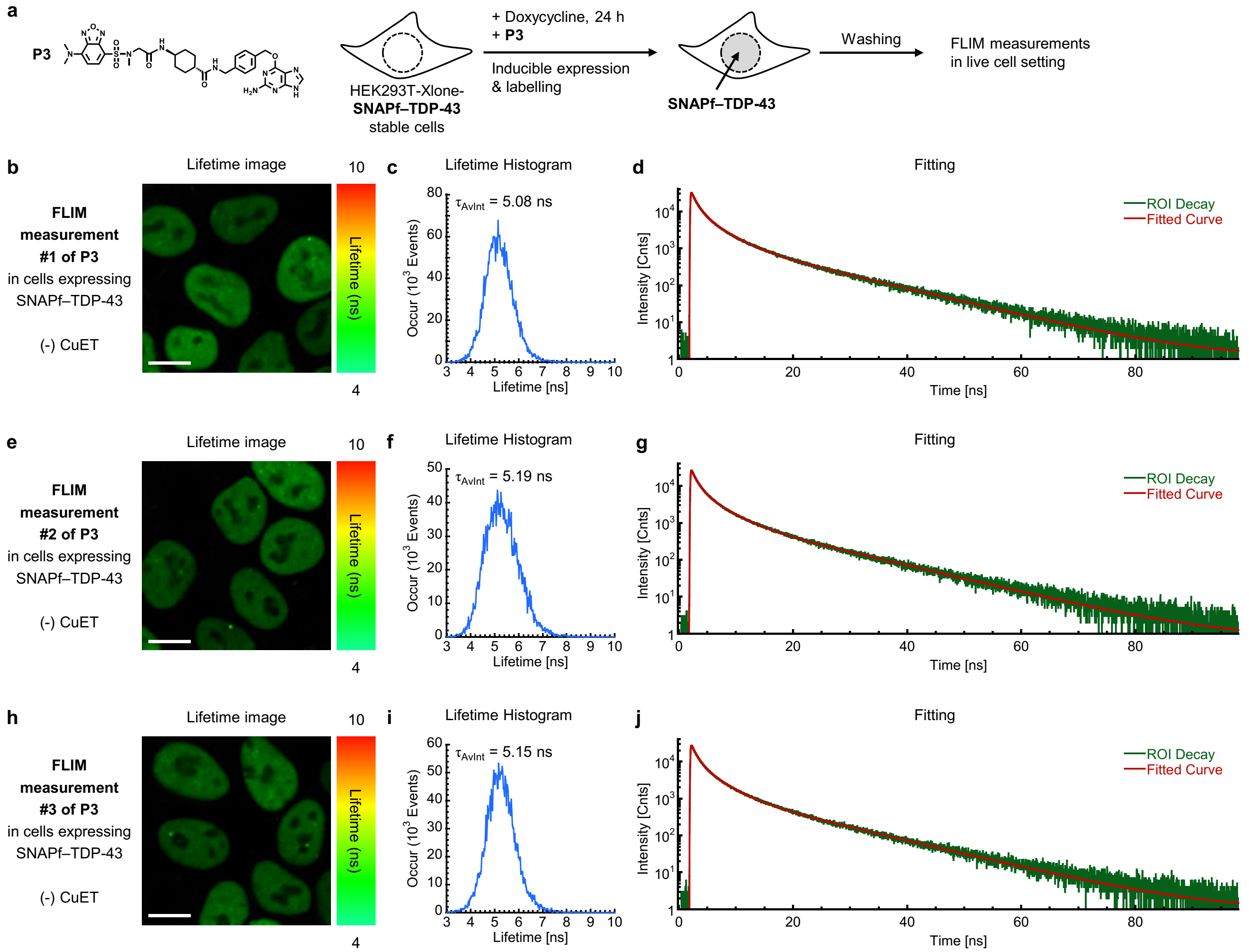

Figure S11. FLIM measurements of P3 with live cells stably expressing SNAPf–TDP-43 without CuET treatment. (**a**) The stable cells were treated with doxycycline (140 ng/mL) and **P3** (0.5 µM) for 24 hours for the inducible expression and labeling of SNAPf–TDP-43, respectively. After the cells were washed with fresh fluorobrite^{TM} DMEM media supplemented with fetal bovine serum (10%) to remove excess probes, the lifetime of **P3** was measured using Zeiss LSM 880 microscope with a PicoQuant-FLIM LSM upgrade KIT. (**b**), (**e**), (**h**) lifetime images from three individual measurements. (**c**), (**f**), (**i**) lifetime histograms from the images b, e, and h, respectively. (**d**), (**g**), (**j**) lifetime decay fitting from the images b, e, and h, respectively. Scale bar = 10 µm.

bioRxiv preprint doi: https://doi.org/10.1101/2023.04.19.537522; this version posted April 21, 2023. The copyright holder for this preprint (which was not certified by peer review) is the author/funder, who has granted bioRxiv a license to display the preprint in perpetuity. It is made available under aCC-BY 4.0 International license.

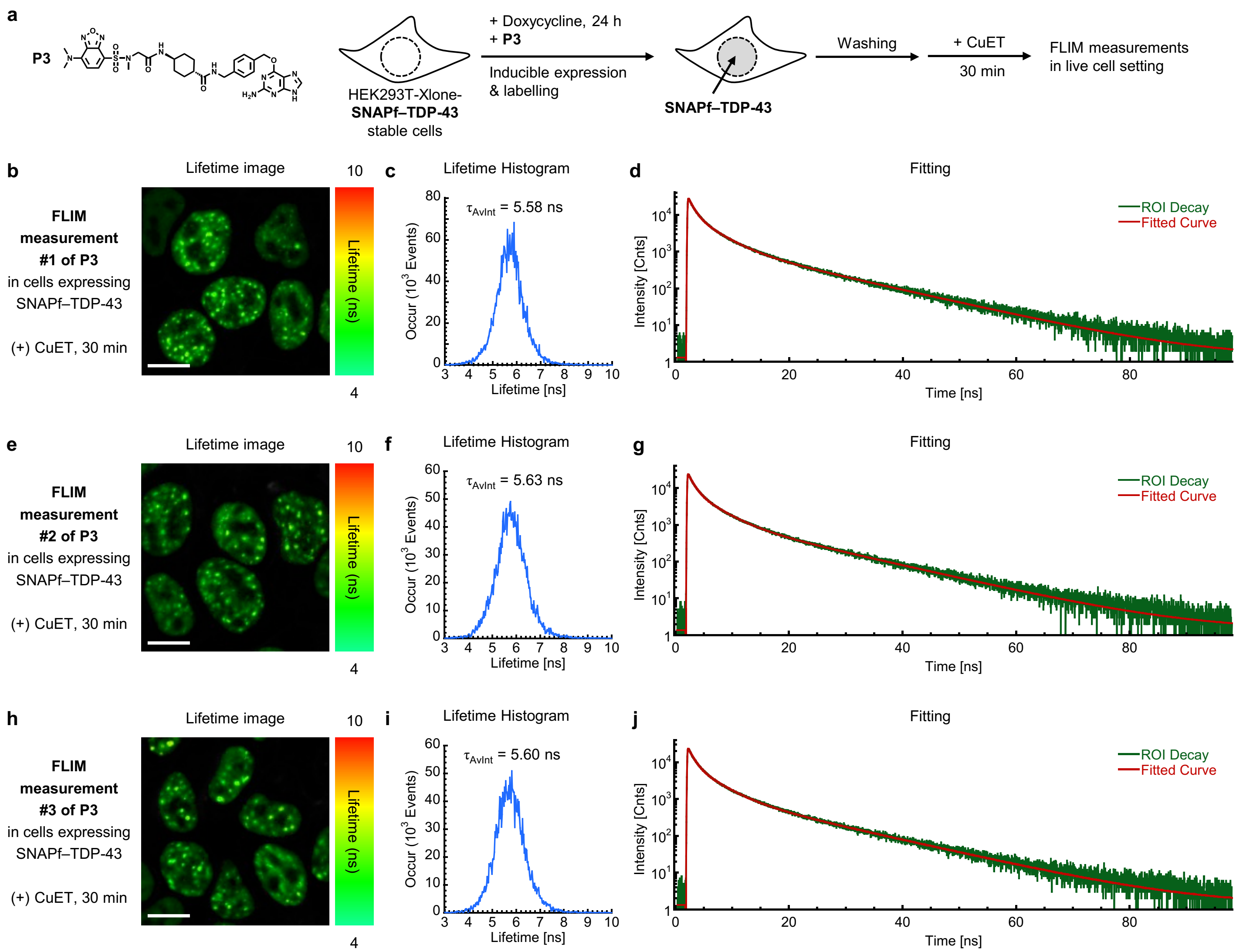

Figure S12. FLIM measurements of P3 with live cells stably expressing SNAPf–TDP-43 upon CuET treatment 30 minutes. (**a**) The stable cells were treated with doxycycline (140 ng/mL) and **P3** (0.5 μM) for 24 hours for the inducible expression and labeling of SNAPf–TDP-43, respectively. After the cells were washed with fresh fluorobrite^{TM} DMEM media supplemented with fetal bovine serum (10%) to remove excess probes, the cells were treated with CuET for 30 minutes at 37 °C under CO_2 (5%) and subsequently the lifetime of **P3** was measured using Zeiss LSM 880 microscope with a PicoQuant-FLIM LSM upgrade KIT. (**b**), (**e**), (**h**) lifetime images from three individual measurements. (**c**), (**f**), (**i**) lifetime histograms from the images b, e, and h, respectively. (**d**), (**g**), (**j**) lifetime decay fitting from the images b, e, and h, respectively. Scale bar = 10 μm.

bioRxiv preprint doi: https://doi.org/10.1101/2023.04.19.537522; this version posted April 21, 2023. The copyright holder for this preprint (which was not certified by peer review) is the author/funder, who has granted bioRxiv a license to display the preprint in perpetuity. It is made available under aCC-BY 4.0 International license.

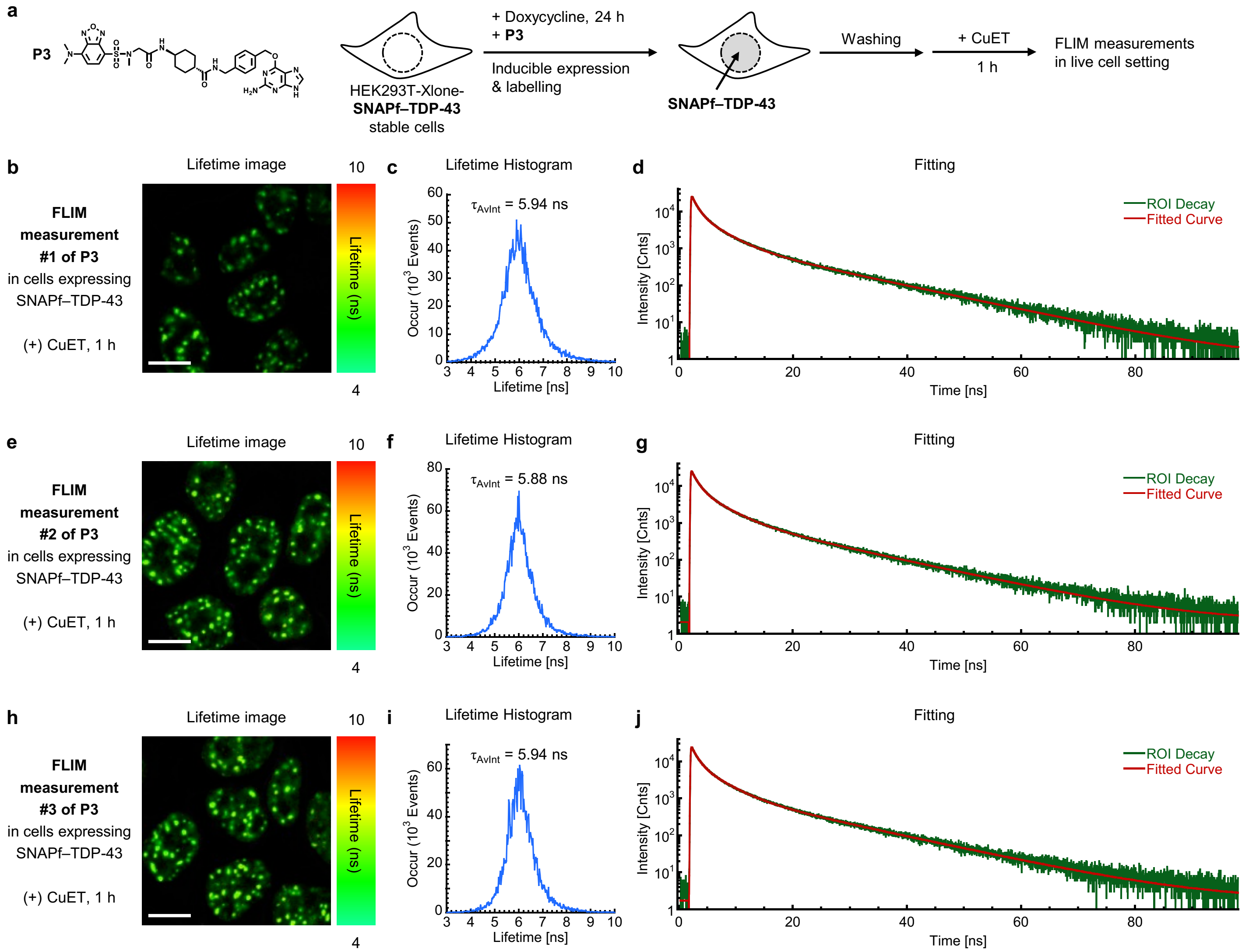

Figure S13. FLIM measurements of P3 with live cells stably expressing SNAPf–TDP-43 upon CuET treatment for 1 hour. (**a**) The stable cells were treated with doxycycline (140 ng/mL) and **P3** (0.5 µM) for 24 hours for the inducible expression and labeling of SNAPf–TDP-43, respectively. After the cells were washed with fresh fluorobrite^{TM} DMEM media supplemented with fetal bovine serum (10%) to remove excess probes, the cells were treated with CuET for 1 hour at 37 °C under CO_2 (5%) and subsequently the lifetime of **P3** was measured using Zeiss LSM 880 microscope with a PicoQuant-FLIM LSM upgrade KIT. (**b**), (**e**), (**h**) lifetime images from three individual measurements. (**c**), (**f**), (**i**) lifetime histograms from the images b, e, and h, respectively. (**d**), (**g**), (**j**) lifetime decay fitting from the images b, e, and h, respectively. Scale bar = 10 µm.

bioRxiv preprint doi: https://doi.org/10.1101/2023.04.19.537522; this version posted April 21, 2023. The copyright holder for this preprint (which was not certified by peer review) is the author/funder, who has granted bioRxiv a license to display the preprint in perpetuity. It is made available under aCC-BY 4.0 International license.

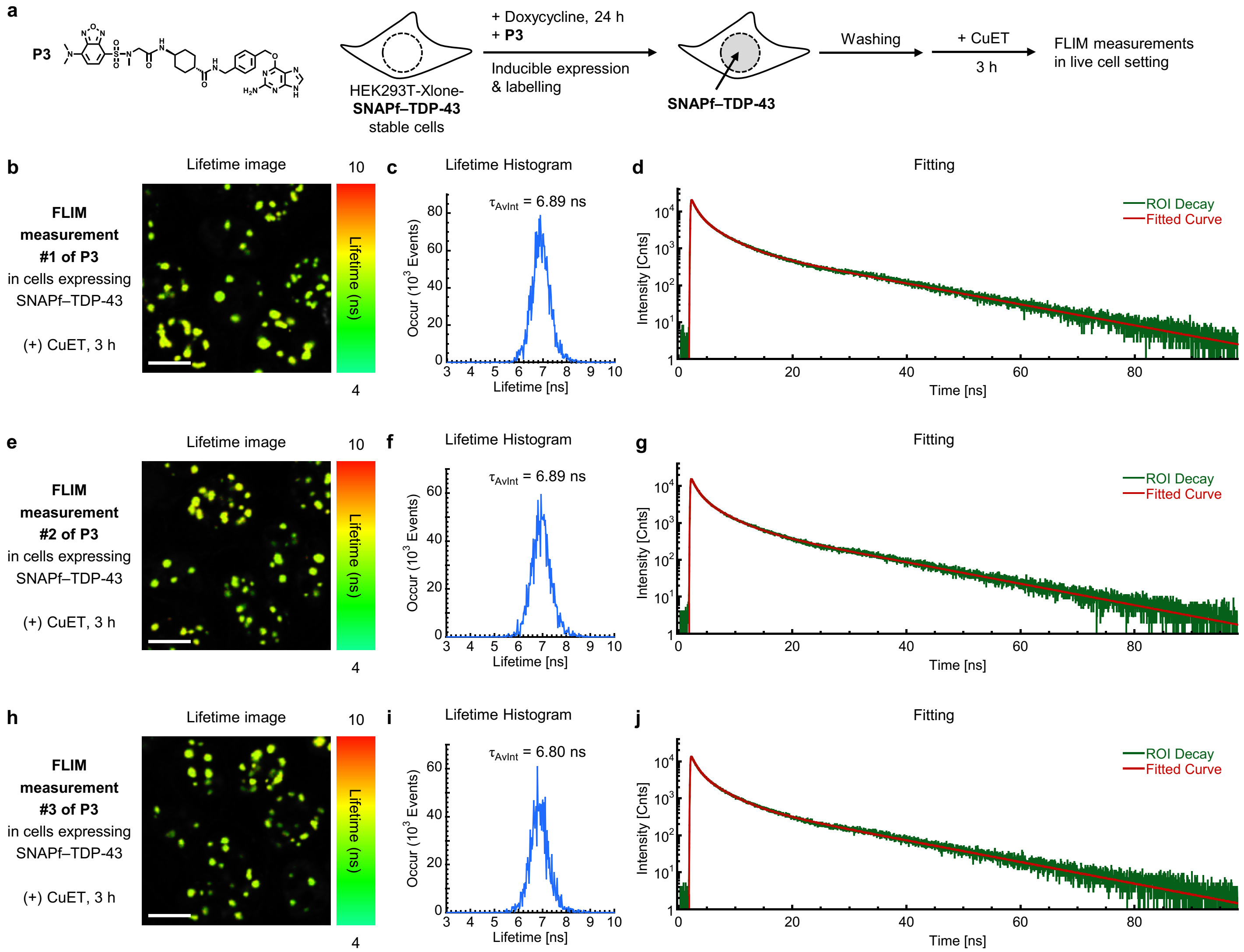

Figure S14. FLIM measurements of P3 with live cells stably expressing SNAPf–TDP-43 upon CuET treatment for 3 hours. The stable cells were treated with doxycycline (140 ng/mL) and **P3** (0.5 μM) for 24 hours for the inducible expression and labeling of SNAPf–TDP-43, respectively. After the cells were washed with fresh fluorobrite™ DMEM media supplemented with fetal bovine serum (10%) to remove excess probes, the cells were treated with CuET for 3 hours at 37 °C under CO_2 (5%) and subsequently the lifetime of **P3** was measured using Zeiss LSM 880 microscope with a PicoQuant-FLIM LSM upgrade KIT. (**b**), (**e**), (**h**) lifetime images from three individual measurements. (**c**), (**f**), (**i**) lifetime histograms from the images b, e, and h, respectively. (**d**), (**g**), (**j**) lifetime decay fitting from the images b, e, and h, respectively. Scale bar = 10 μm.

bioRxiv preprint doi: https://doi.org/10.1101/2023.04.19.537522; this version posted April 21, 2023. The copyright holder for this preprint (which was not certified by peer review) is the author/funder, who has granted bioRxiv a license to display the preprint in perpetuity. It is made available under aCC-BY 4.0 International license.

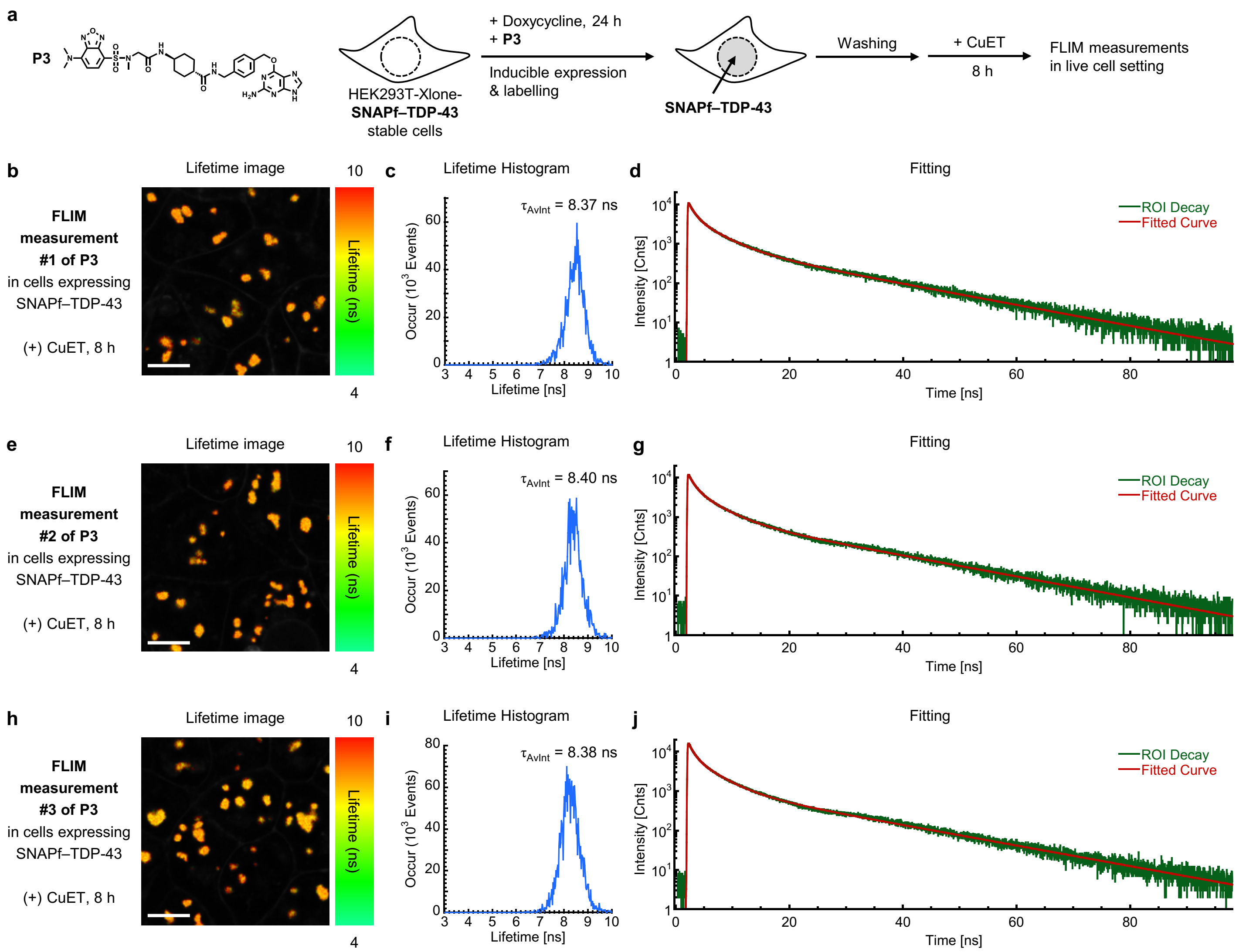

Figure S15. FLIM measurements of P3 with live cells stably expressing SNAPf–TDP-43 upon CuET treatment for 8 hours. The stable cells were treated with doxycycline (140 ng/mL) and **P3** (0.5 μM) for 24 hours for the inducible expression and labeling of SNAPf–TDP-43, respectively. After the cells were washed with fresh fluorobrite™ DMEM media supplemented with fetal bovine serum (10%) to remove excess probes, the cells were treated with CuET for 8 hours at 37 °C under CO_2 (5%) and subsequently the lifetime of **P3** was measured using Zeiss LSM 880 microscope with a PicoQuant-FLIM LSM upgrade KIT. (**b**), (**e**), (**h**) lifetime images from three individual measurements. (**c**), (**f**), (**i**) lifetime histograms from the images b, e, and h, respectively. (**d**), (**g**), (**j**) lifetime decay fitting from the images b, e, and h, respectively. Scale bar = 10 μm.

bioRxiv preprint doi: https://doi.org/10.1101/2023.04.19.537522; this version posted April 21, 2023. The copyright holder for this preprint (which was not certified by peer review) is the author/funder, who has granted bioRxiv a license to display the preprint in perpetuity. It is made available under aCC-BY 4.0 International license.

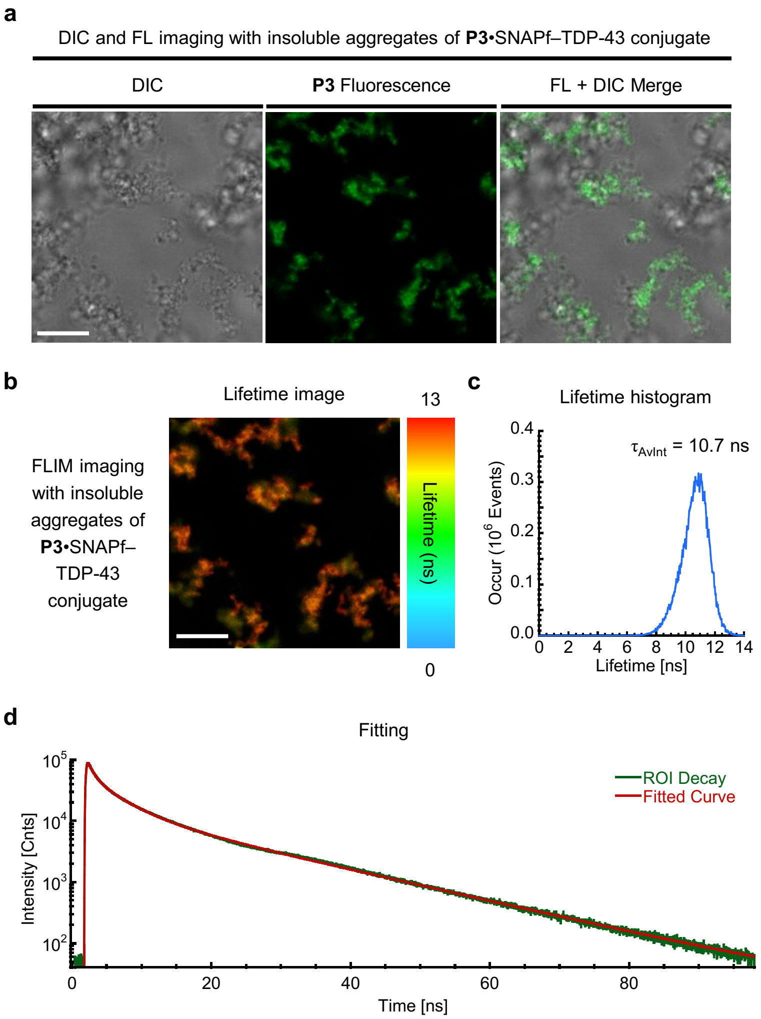

Figure S16. Confocal and FLIM imaging experiments with insoluble aggregates of P3•SNAPf–TDP-43 conjugate. (**a**) DIC and FL imaging, (**b**) lifetime imaging, (**c**) lifetime histogram, (**d**) lifetime decay fitting with insoluble aggregates of **P3**•SNAPf–TDP-43 conjugate. Scale bar = 10 μm. The insoluble aggregates of **P3**•SNAPf–TDP-43 conjugate were generated from recombinantly purified SNAPf–TDP-43–TEV–Halo fusion protein. The protein was constructed and purified from *E. Coli* according to the previously reported procedures.[73] For labeling, the fusion protein (10 μM) was incubated with **P3** (5 μM) at 37 °C for 30 minutes in HEPES buffer (20 mM, pH 7.5, 140 mM NaCl) containing DTT (1 mM). After labeling, the protein conjugate solution was treated with TEV protease (0.75 μM) and PEG3350 (5%) at room temperature for 1 hour to induce the insoluble aggregates.

bioRxiv preprint doi: https://doi.org/10.1101/2023.04.19.537522; this version posted April 21, 2023. The copyright holder for this preprint (which was not certified by peer review) is the author/funder, who has granted bioRxiv a license to display the preprint in perpetuity. It is made available under aCC-BY 4.0 International license.

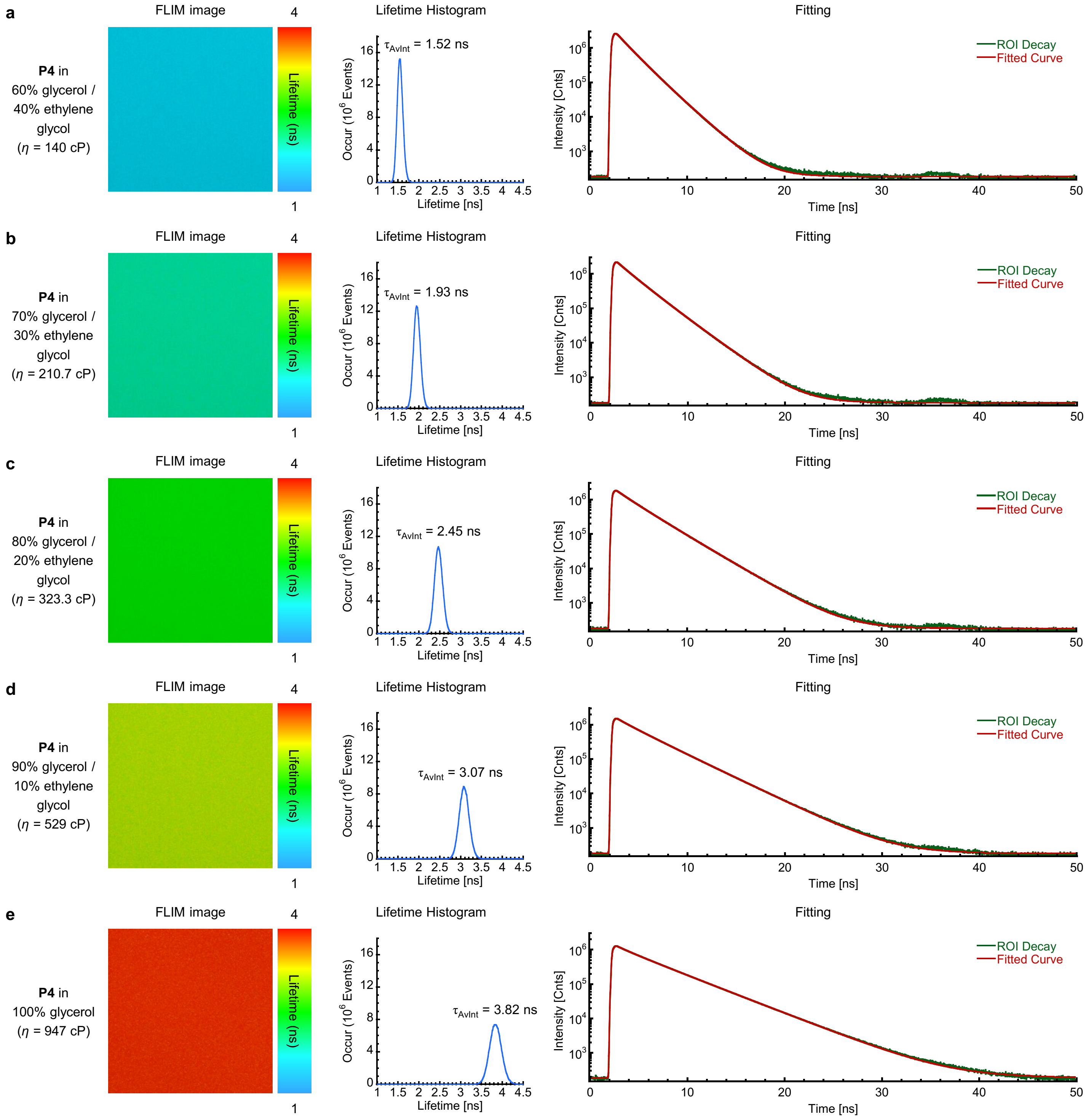

Figure S17. Average fluorescence lifetime (τ_{AvInt}) of P4 increases as the viscosity (η) of solvent mixtures tested increases. The fluorescence lifetime images (left panel), lifetime histograms (center panel), and lifetime decay curves (right panel) of **P4** in mixtures of glycerol and ethylene glycol with different volume fractions

bioRxiv preprint doi: https://doi.org/10.1101/2023.04.19.537522; this version posted April 21, 2023. The copyright holder for this preprint (which was not certified by peer review) is the author/funder, who has granted bioRxiv a license to display the preprint in perpetuity. It is made available under aCC-BY 4.0 International license.

(v/v, %). (**a**) in 60% glycerol and 40% ethylene glycol, (**b**) in 70% glycerol and 30% ethylene glycol, (**c**) in 80% glycerol and 20% ethylene glycol, (**d**) in 90% glycerol and 10% ethylene glycol, (**e**) in 100% glycerol.

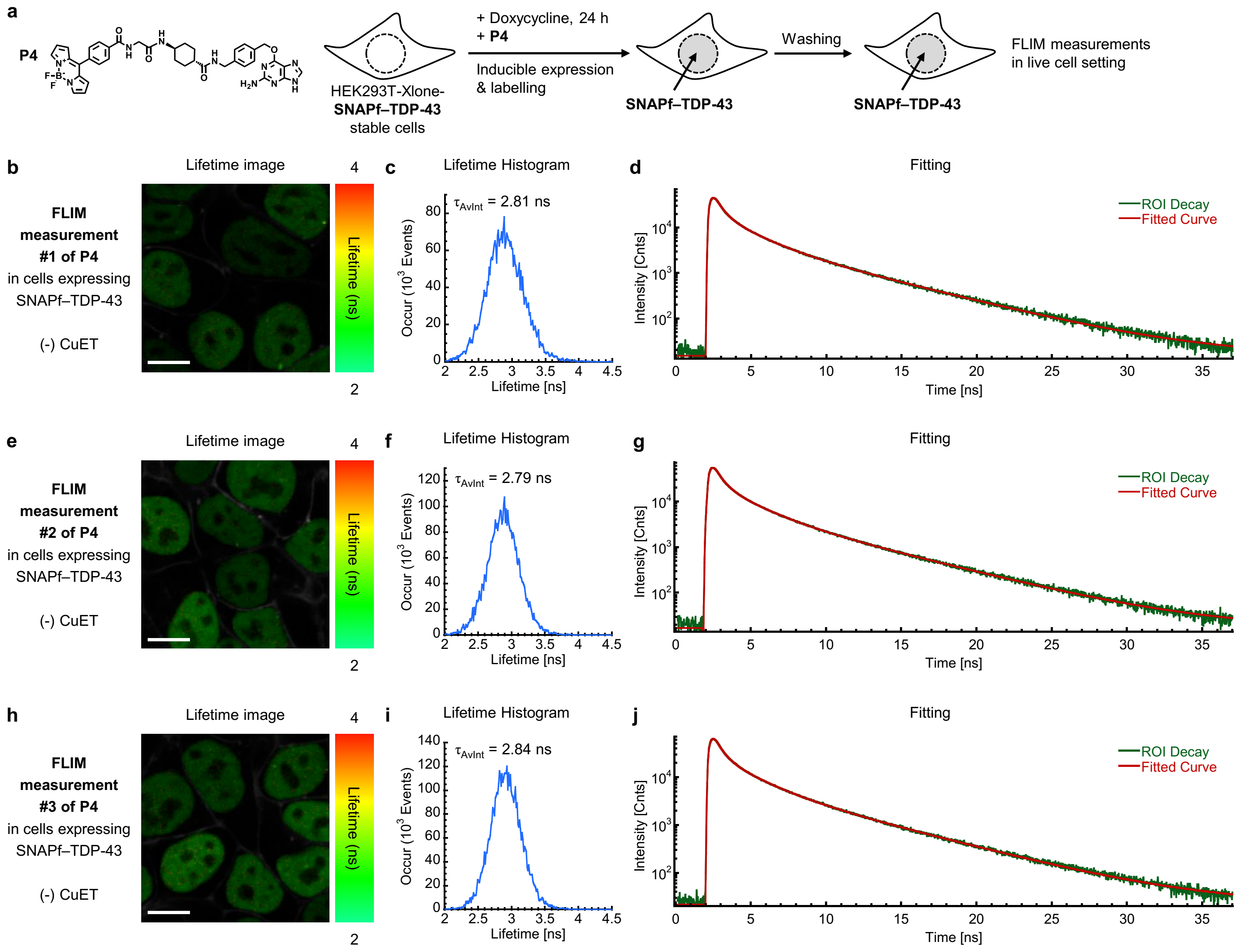

Figure S18. FLIM measurements of P4 with live cells stably expressing SNAPf–TDP-43 without CuET treatment. (**a**) The stable cells were treated with doxycycline (140 ng/mL) and **P4** (0.5 μM) for 24 hours for the inducible expression and labeling of SNAPf–TDP-43, respectively. After the cells were washed with fresh fluorobrite^{TM} DMEM media supplemented with fetal bovine serum (10%) to remove excess probes, the lifetime of **P4** was measured using Zeiss LSM 880 microscope with a PicoQuant-FLIM LSM upgrade KIT. (**b**), (**e**), (**h**) lifetime images from three individual measurements, (**c**), (**f**), (**i**) lifetime histograms from the images b, e, and h, respectively, (**d**), (**g**), (**j**) lifetime decay fitting from the images b, e, and h, respectively. Scale bar = 10 μm.

bioRxiv preprint doi: https://doi.org/10.1101/2023.04.19.537522; this version posted April 21, 2023. The copyright holder for this preprint (which was not certified by peer review) is the author/funder, who has granted bioRxiv a license to display the preprint in perpetuity. It is made available under aCC-BY 4.0 International license.

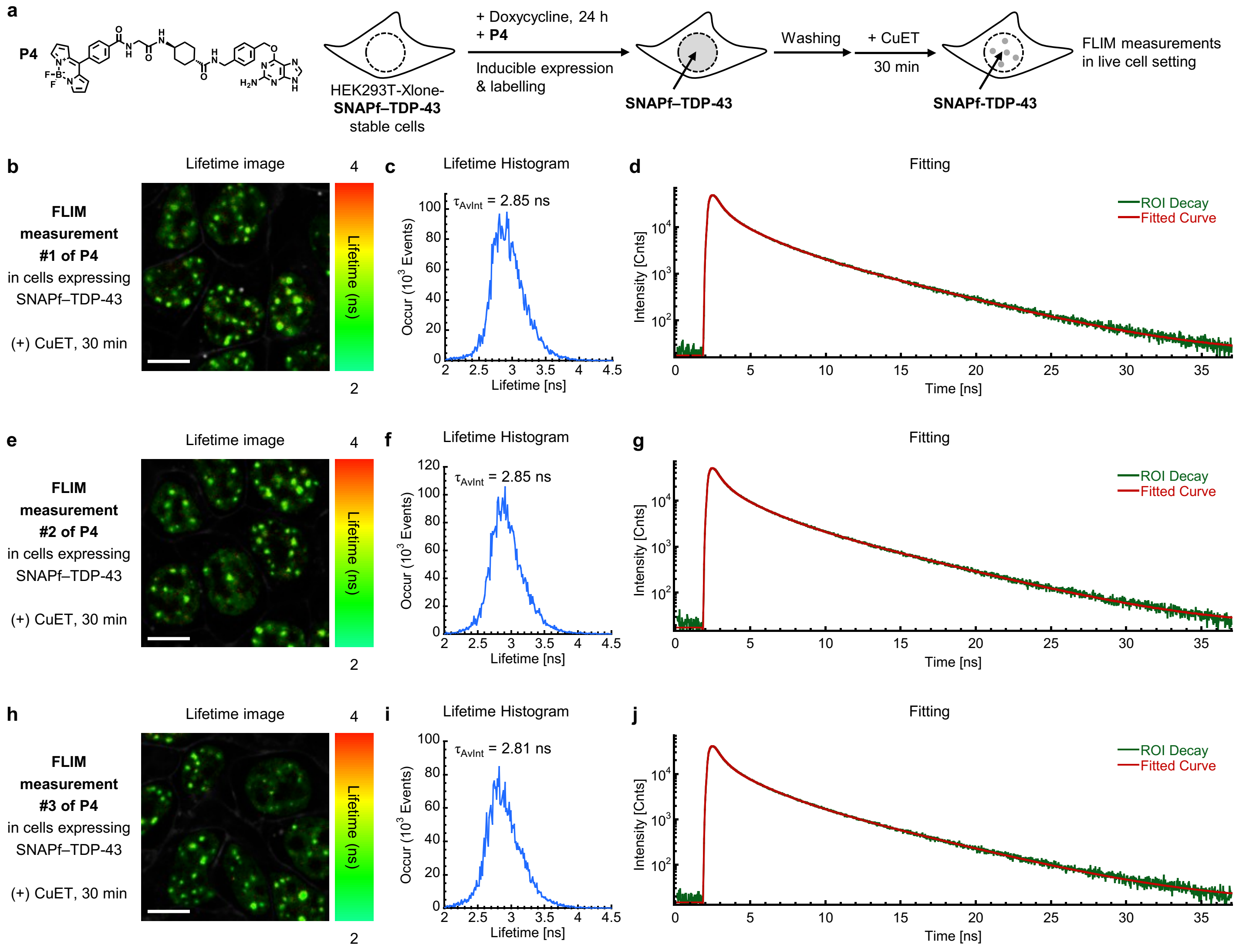

Figure S19. FLIM measurements of P4 with live cells stably expressing SNAPf–TDP-43 upon CuET treatment for 30 minutes. (**a**) The stable cells were treated with doxycycline (140 ng/mL) and **P4** (0.5 µM) for 24 hours for the inducible expression and labeling of SNAPf–TDP-43, respectively. After the cells were washed with fresh fluorobrite^{TM} DMEM media supplemented with fetal bovine serum (10%) to remove excess probes, the cells were treated with CuET for 30 minutes at 37 °C under CO_2 (5%) and subsequently the lifetime of **P4** was measured using Zeiss LSM 880 microscope with a PicoQuant-FLIM LSM upgrade KIT. (**b**), (**e**), (**h**) lifetime images from three individual measurements. (**c**), (**f**), (**i**) lifetime histograms from the images b, e, and h, respectively. (**d**), (**g**), (**j**) lifetime decay fitting from the images b, e, and h, respectively. Scale bar = 10 µm.

bioRxiv preprint doi: https://doi.org/10.1101/2023.04.19.537522; this version posted April 21, 2023. The copyright holder for this preprint (which was not certified by peer review) is the author/funder, who has granted bioRxiv a license to display the preprint in perpetuity. It is made available under aCC-BY 4.0 International license.

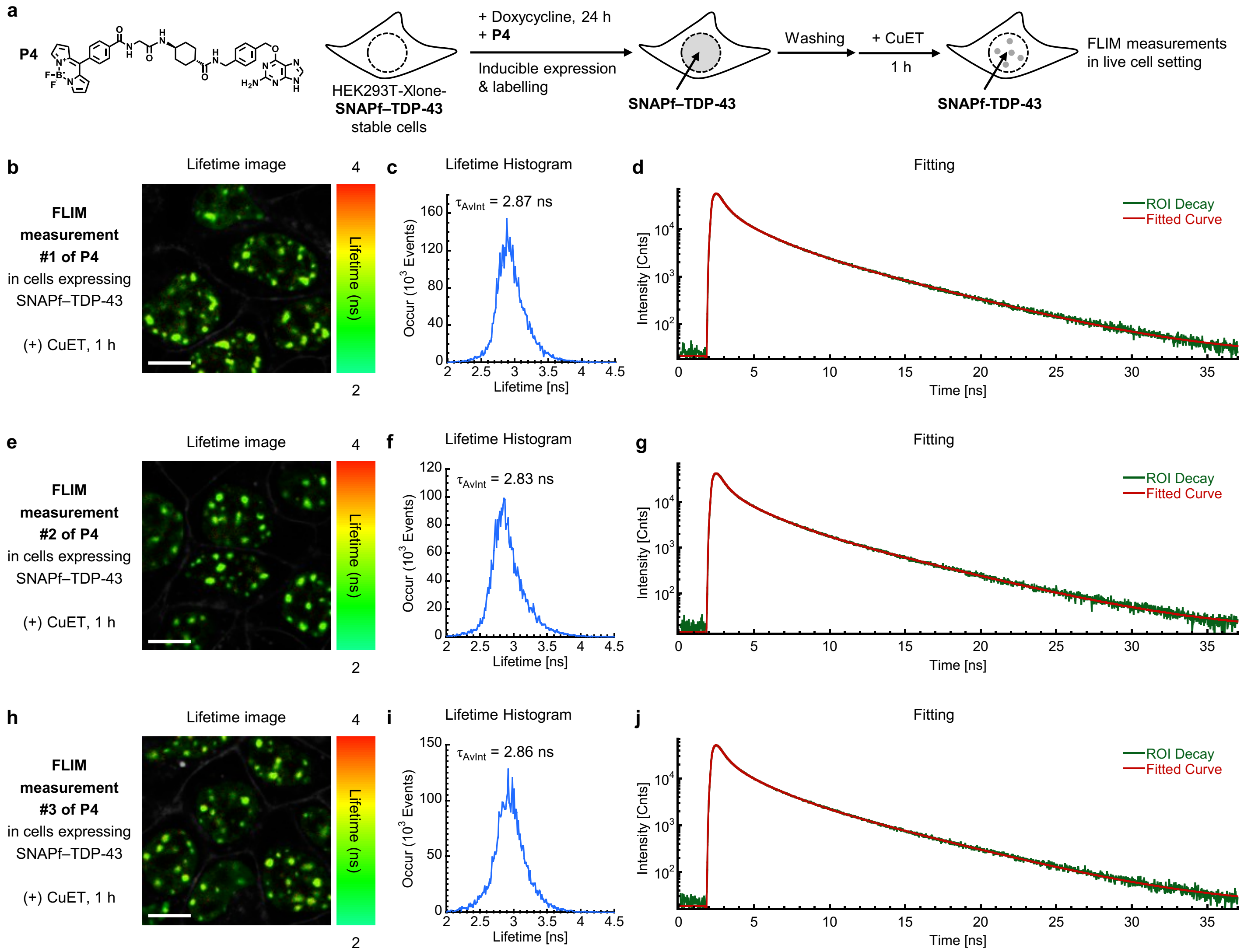

Figure S20. FLIM measurements of P4 with live cells stably expressing SNAPf–TDP-43 upon CuET treatment for 1 hour. (**a**) The stable cells were treated with doxycycline (140 ng/mL) and **P4** (0.5 µM) for 24 hours for the inducible expression and labeling of SNAPf–TDP-43, respectively. After the cells were washed with fresh fluorobrite™ DMEM media supplemented with fetal bovine serum (10%) to remove excess probes, the cells were treated with CuET for 1 hour at 37 °C under CO_2 (5%) and subsequently the lifetime of **P4** was measured using Zeiss LSM 880 microscope with a PicoQuant-FLIM LSM upgrade KIT. (**b**), (**e**), (**h**) lifetime images from three individual measurements. (**c**), (**f**), (**i**) lifetime histograms from the images b, e, and h, respectively. (**d**), (**g**), (**j**) lifetime decay fitting from the images b, e, and h, respectively. Scale bar = 10 µm.

bioRxiv preprint doi: https://doi.org/10.1101/2023.04.19.537522; this version posted April 21, 2023. The copyright holder for this preprint (which was not certified by peer review) is the author/funder, who has granted bioRxiv a license to display the preprint in perpetuity. It is made available under aCC-BY 4.0 International license.

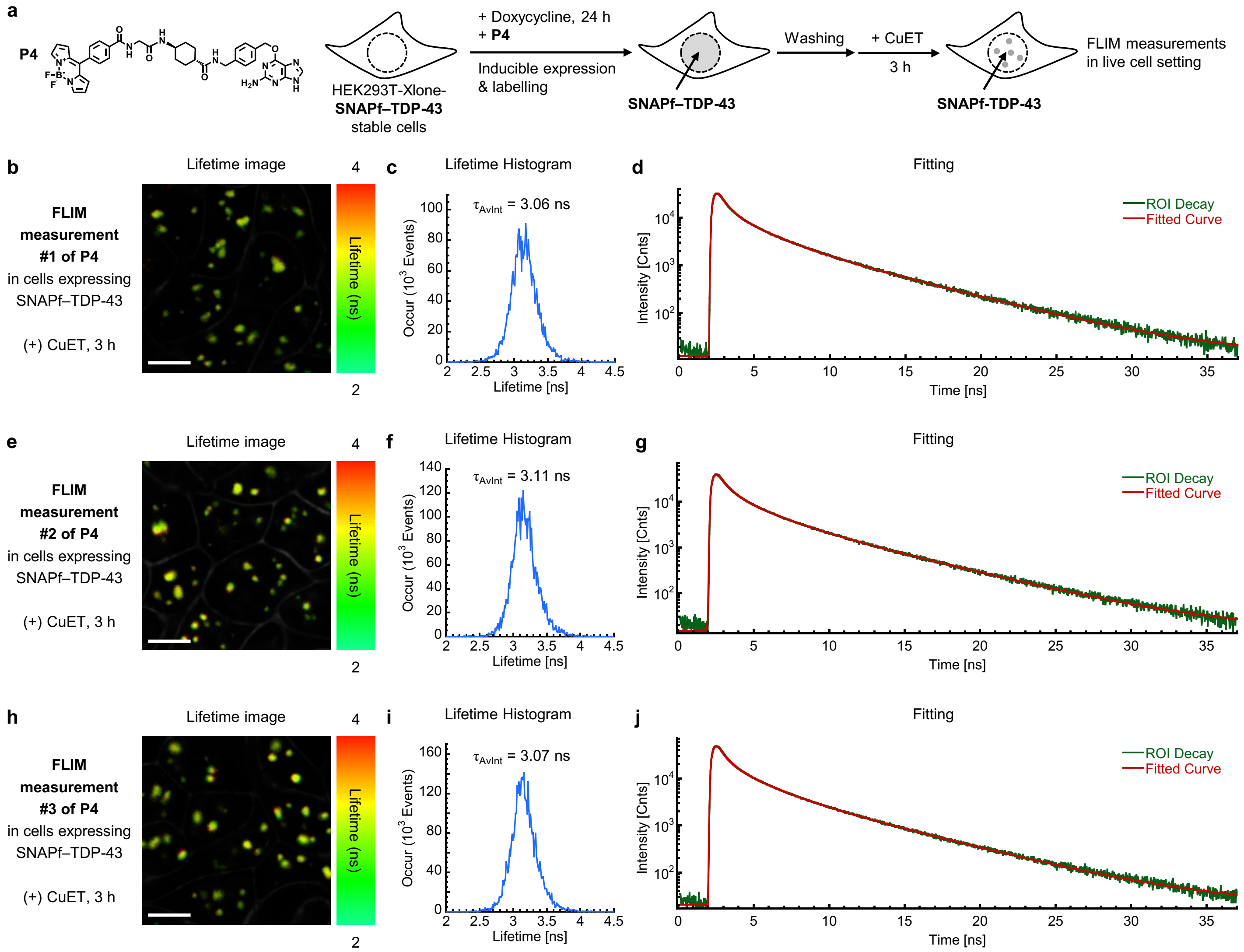

Figure S21. FLIM measurements of P4 with live cells stably expressing SNAPf–TDP-43 upon CuET treatment for 3 hours. (**a**) The stable cells were treated with doxycycline (140 ng/mL) and **P4** (0.5 μM) for 24 hours for the inducible expression and labeling of SNAPf–TDP-43, respectively. After the cells were washed with fresh fluorobrite™ DMEM media supplemented with fetal bovine serum (10%) to remove excess probes, the cells were treated with CuET for 3 hours at 37 °C under CO_2 (5%) and subsequently the lifetime of **P4** was measured using Zeiss LSM 880 microscope with a PicoQuant-FLIM LSM upgrade KIT. (**b**), (**e**), (**h**) lifetime images from three individual measurements. (**c**), (**f**), (**i**) lifetime histograms from the images b, e, and h, respectively. (**d**), (**g**), (**j**) lifetime decay fitting from the images b, e, and h, respectively. Scale bar = 10 μm.

bioRxiv preprint doi: https://doi.org/10.1101/2023.04.19.537522; this version posted April 21, 2023. The copyright holder for this preprint (which was not certified by peer review) is the author/funder, who has granted bioRxiv a license to display the preprint in perpetuity. It is made available under aCC-BY 4.0 International license.

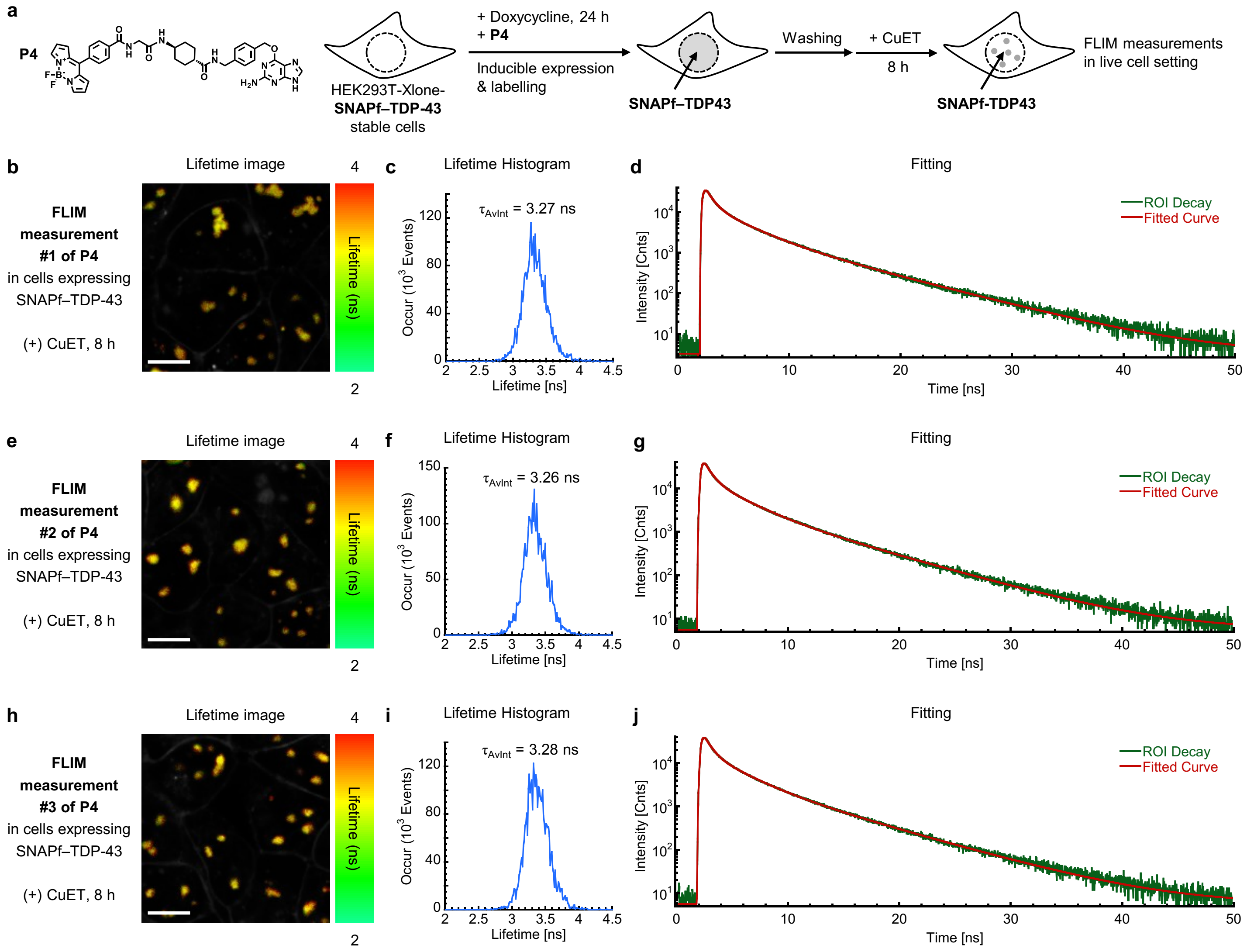

Figure S22. FLIM measurements of P4 with live cells stably expressing SNAPf–TDP-43 upon CuET treatment for 8 hours. (**a**) The stable cells were treated with doxycycline (140 ng/mL) and **P4** (0.5 µM) for 24 hours for the inducible expression and labeling of SNAPf–TDP-43, respectively. After the cells were washed with fresh fluorobrite™ DMEM media supplemented with fetal bovine serum (10%) to remove excess probes, the cells were treated with CuET for 8 hours at 37 °C under CO_2 (5%) and subsequently the lifetime of **P4** was measured using Zeiss LSM 880 microscope with a PicoQuant-FLIM LSM upgrade KIT. (**b**), (**e**), (**h**) lifetime images from three individual measurements. (**c**), (**f**), (**i**) lifetime histograms from the images b, e, and h, respectively. (**d**), (**g**), (**j**) lifetime decay fitting from the images b, e, and h, respectively. Scale bar = 10 µm.

bioRxiv preprint doi: https://doi.org/10.1101/2023.04.19.537522; this version posted April 21, 2023. The copyright holder for this preprint (which was not certified by peer review) is the author/funder, who has granted bioRxiv a license to display the preprint in perpetuity. It is made available under aCC-BY 4.0 International license.

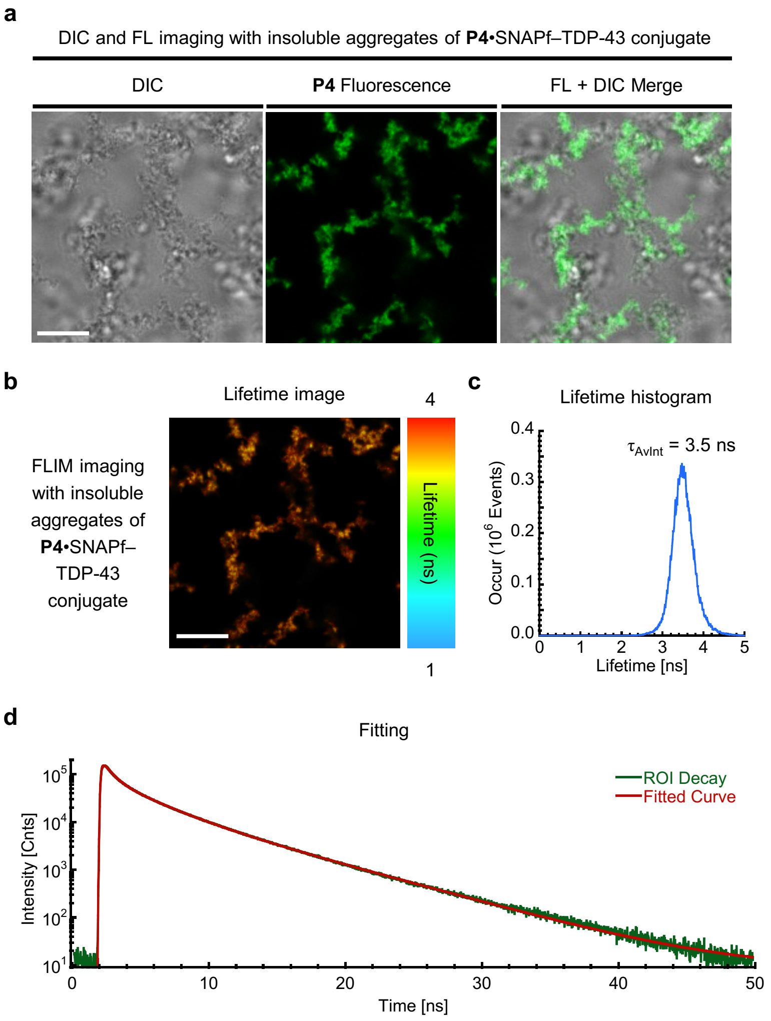

Figure S23. Confocal and FLIM imaging experiments with insoluble aggregates of P4•SNAPf–TDP-43 conjugate. (**a**) DIC and FL imaging, (**b**) lifetime imaging, (**c**) lifetime histogram, (**d**) lifetime decay fitting with insoluble aggregates of **P4**•SNAPf–TDP-43 conjugate. Scale bar = 10 µm. The insoluble aggregates of **P4**•SNAPf–TDP-43 conjugate were generated from recombinantly purified SNAPf–TDP-43–TEV–Halo fusion protein. The protein was constructed and purified from *E. Coli* according to the previously reported procedures.[73] For labeling, the fusion protein (10 µM) was incubated with **P4** (5 µM) at 37 °C for 30 minutes in HEPES buffer (20 mM, pH 7.5, 140 mM NaCl) containing DTT (1 mM). After labeling, the protein conjugate solution was treated with TEV protease (0.75 µM) and PEG3350 (5%) at room temperature for 1 hour to induce the insoluble aggregates.

bioRxiv preprint doi: https://doi.org/10.1101/2023.04.19.537522; this version posted April 21, 2023. The copyright holder for this preprint (which was not certified by peer review) is the author/funder, who has granted bioRxiv a license to display the preprint in perpetuity. It is made available under aCC-BY 4.0 International license.

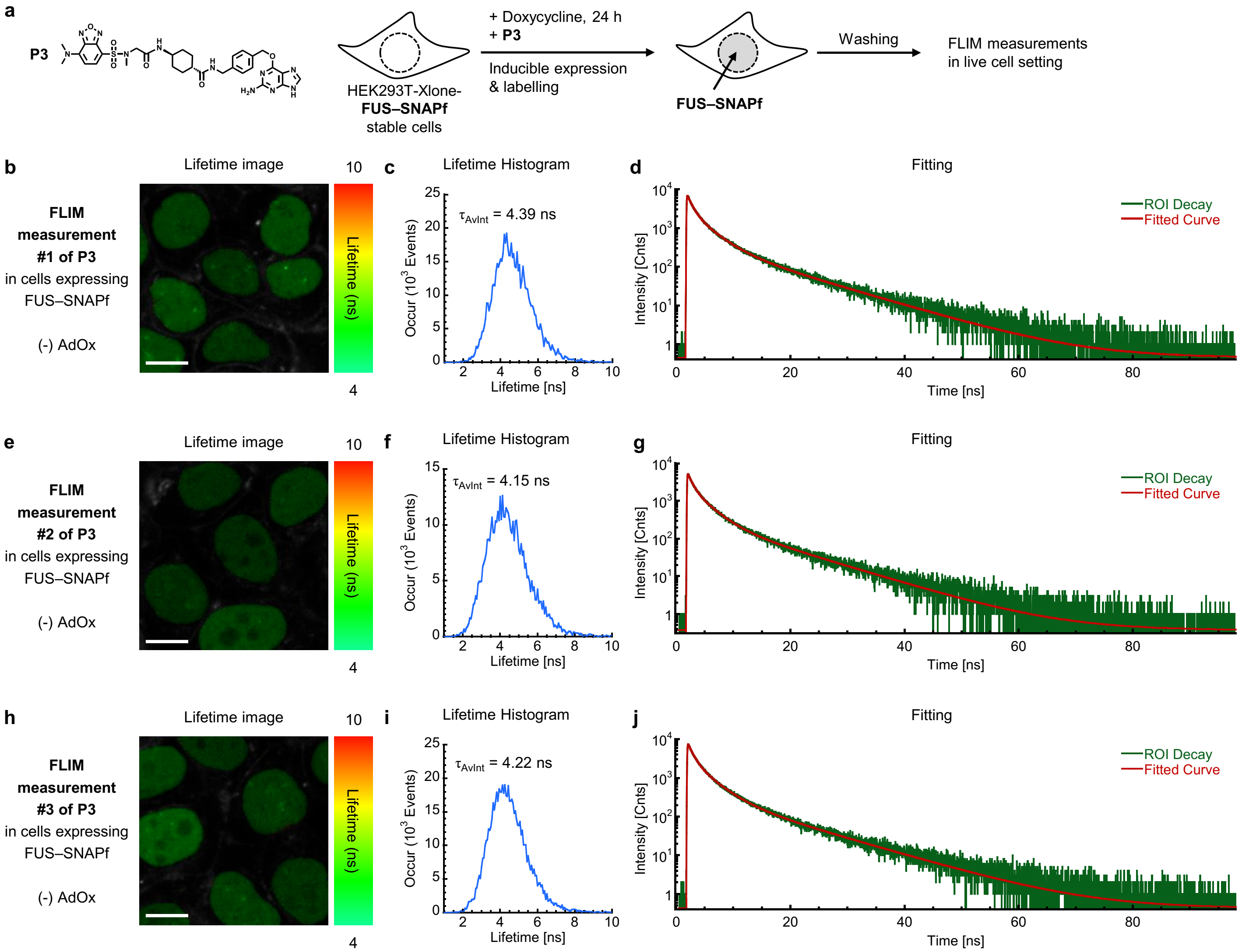

Figure S24. FLIM measurements of P3 with live cells stably expressing FUS–SNAPf without AdOx pretreatment. (**a**) The stable cells were treated with doxycycline (140 ng/mL) and **P3** (0.5 µM) for 24 hours for the inducible expression and labeling of FUS–SNAPf, respectively. After the cells were washed with fresh fluorobrite™ DMEM media supplemented with fetal bovine serum (10%) to remove excess probes, the lifetime of **P3** was measured using Zeiss LSM 880 microscope with a PicoQuant-FLIM LSM upgrade KIT. (**b**), (**e**), (**h**) lifetime images from three individual measurements. (**c**), (**f**), (**i**) lifetime histograms from the images b, e, and h, respectively. (**d**), (**g**), (**j**) lifetime decay fitting from the images b, e, and h, respectively. Scale bar = 10 µm.

bioRxiv preprint doi: https://doi.org/10.1101/2023.04.19.537522; this version posted April 21, 2023. The copyright holder for this preprint (which was not certified by peer review) is the author/funder, who has granted bioRxiv a license to display the preprint in perpetuity. It is made available under aCC-BY 4.0 International license.

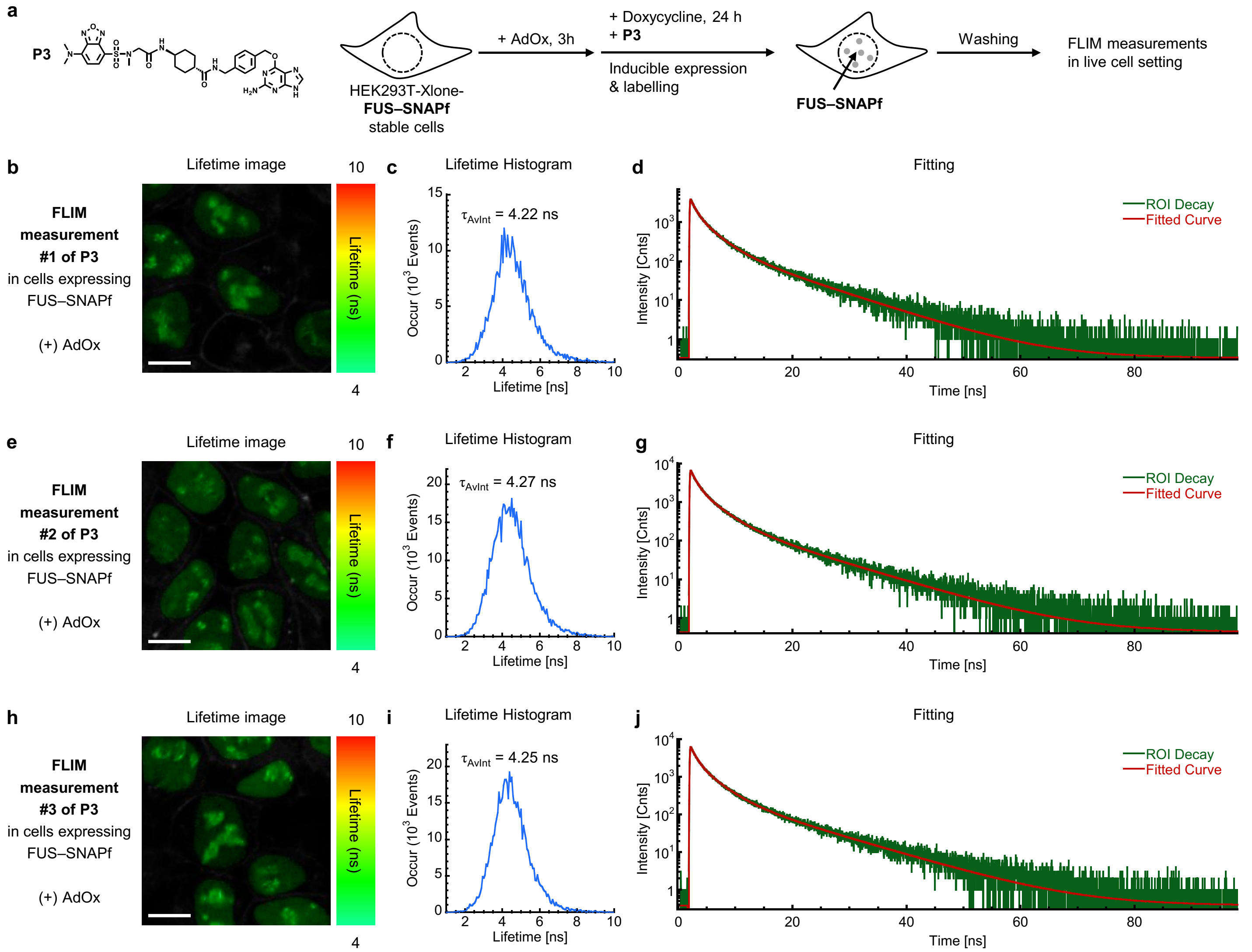

Figure S25. FLIM measurements of P3 with live cells stably expressing FUS–SNAPf with AdOx pretreatment. (**a**) The stable cells were pretreated with AdOx (25 μM) 3 hours before they were further treated with doxycycline (140 ng/mL) and **P3** (0.5 μM) for 24 hours for the inducible expression and labeling of FUS–SNAPf, respectively. After the cells were washed with fresh fluorobrite™ DMEM media supplemented with fetal bovine serum (10%) to remove excess probes, the lifetime of **P3** was measured using Zeiss LSM 880 microscope with a PicoQuant-FLIM LSM upgrade KIT. (**b**), (**e**), (**h**) lifetime images from three individual measurements. (**c**), (**f**), (**i**) lifetime histograms from the images b, e, and h, respectively. (**d**), (**g**), (**j**) lifetime decay fitting from the images b, e, and h, respectively. Scale bar = 10 μm.

bioRxiv preprint doi: https://doi.org/10.1101/2023.04.19.537522; this version posted April 21, 2023. The copyright holder for this preprint (which was not certified by peer review) is the author/funder, who has granted bioRxiv a license to display the preprint in perpetuity. It is made available under aCC-BY 4.0 International license.

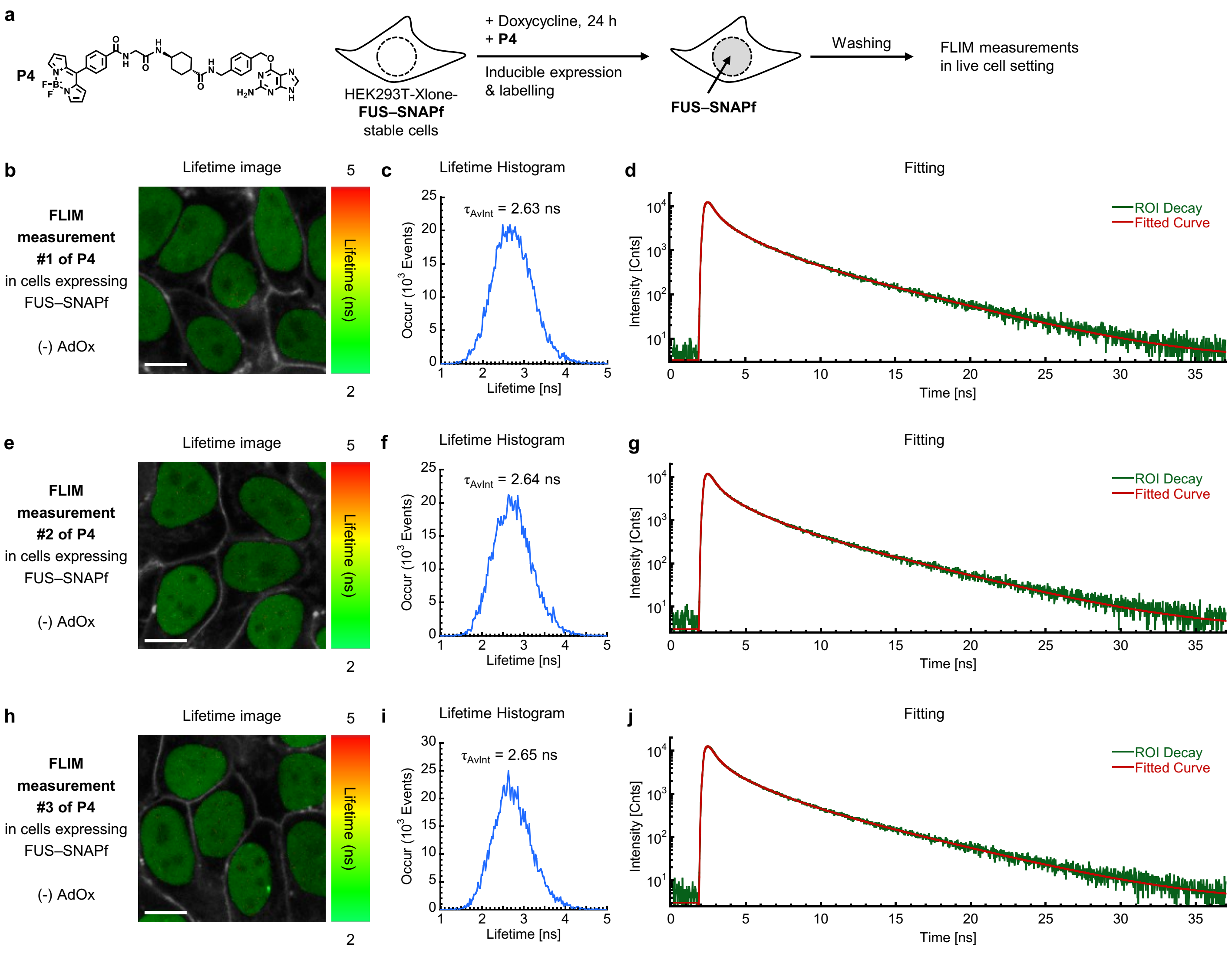

Figure S26. FLIM measurements of P4 with live cells stably expressing FUS–SNAPf without AdOx pretreatment. (**a**) The stable cells were treated with doxycycline (140 ng/mL) and **P4** (0.5 µM) for 24 hours for the inducible expression and labeling of FUS–SNAPf, respectively. After the cells were washed with fresh fluorobrite^{TM} DMEM media supplemented with fetal bovine serum (10%) to remove excess probes, the lifetime of **P4** was measured using Zeiss LSM 880 microscope with a PicoQuant-FLIM LSM upgrade KIT. (**b**), (**e**), (**h**) lifetime images from three individual measurements. (**c**), (**f**), (**i**) lifetime histograms from the images b, e, and h, respectively. (**d**), (**g**), (**j**) lifetime decay fitting from the images b, e, and h, respectively. Scale bar = 10 µm.

bioRxiv preprint doi: https://doi.org/10.1101/2023.04.19.537522; this version posted April 21, 2023. The copyright holder for this preprint (which was not certified by peer review) is the author/funder, who has granted bioRxiv a license to display the preprint in perpetuity. It is made available under aCC-BY 4.0 International license.

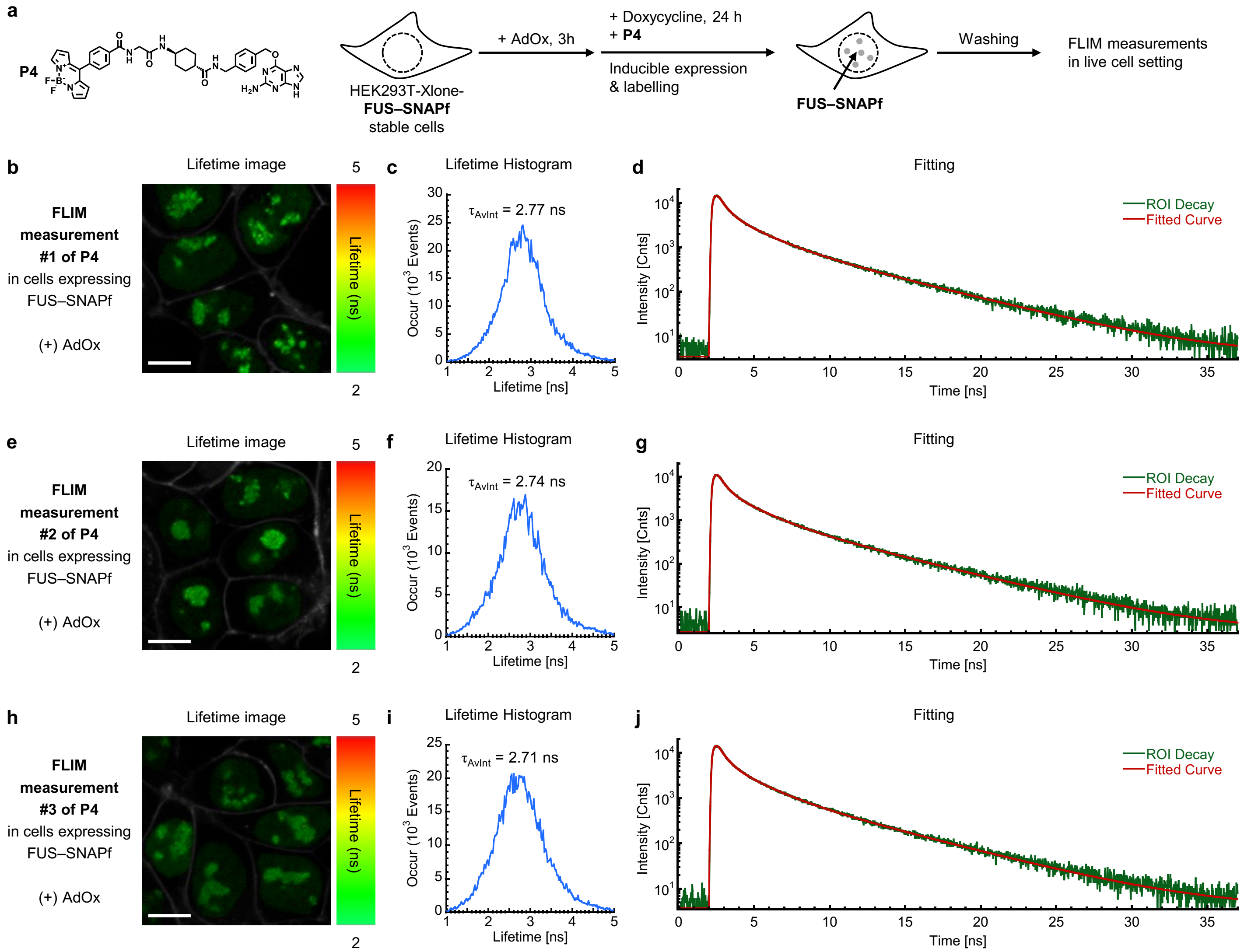

Figure S27. FLIM measurements of P4 with live cells stably expressing FUS–SNAPf with AdOx pretreatment. (**a**) The stable cells were pretreated with AdOx (25 µM) 3 hours before they were further treated with doxycycline (140 ng/mL) and **P4** (0.5 µM) for 24 hours for the inducible expression and labeling of FUS–SNAPf, respectively. After the cells were washed with fresh fluorobrite™ DMEM media supplemented with fetal bovine serum (10%) to remove excess probes, the lifetime of **P4** was measured using Zeiss LSM 880 microscope with a PicoQuant-FLIM LSM upgrade KIT. (**b**), (**e**), (**h**) lifetime images from three individual measurements. (**c**), (**f**), (**i**) lifetime histograms from the images b, e, and h, respectively. (**d**), (**g**), (**j**) lifetime decay fitting from the images b, e, and h, respectively. Scale bar = 10 µm.

bioRxiv preprint doi: https://doi.org/10.1101/2023.04.19.537522; this version posted April 21, 2023. The copyright holder for this preprint (which was not certified by peer review) is the author/funder, who has granted bioRxiv a license to display the preprint in perpetuity. It is made available under aCC-BY 4.0 International license.

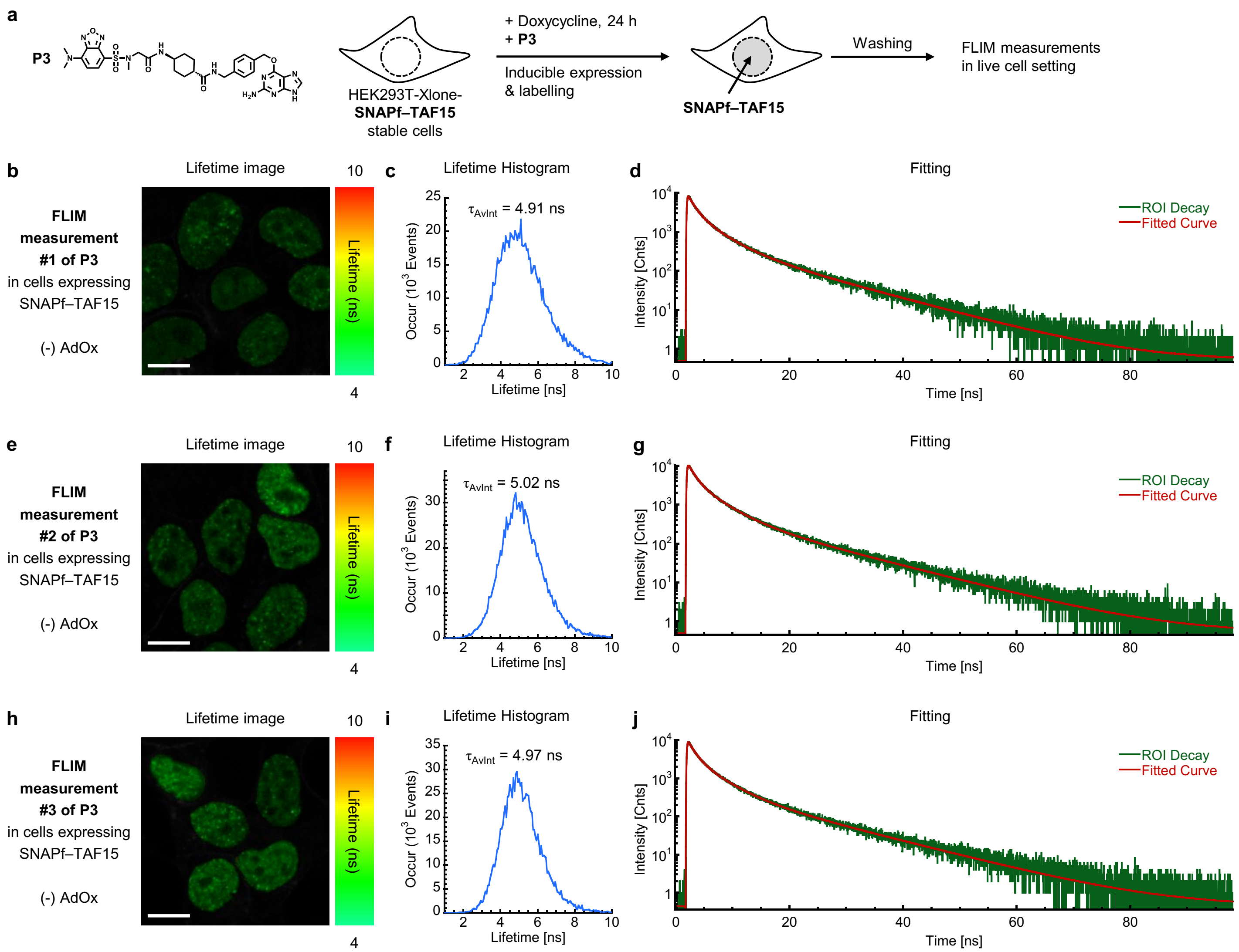

Figure S28. FLIM measurements of P3 with live cells stably expressing SNAPf–TAF15 without AdOx pretreatment. (**a**) The stable cells were treated with doxycycline (140 ng/mL) and **P3** (0.5 µM) for 24 hours for the inducible expression and labeling of SNAPf–TAF15, respectively. After the cells were washed with fresh fluorobrite™ DMEM media supplemented with fetal bovine serum (10%) to remove excess probes, the lifetime of **P3** was measured using Zeiss LSM 880 microscope with a PicoQuant-FLIM LSM upgrade KIT. (**b**), (**e**), (**h**) lifetime images from three individual measurements. (**c**), (**f**), (**i**) lifetime histograms from the images b, e, and h, respectively. (**d**), (**g**), (**j**) lifetime decay fitting from the images b, e, and h, respectively. Scale bar = 10 µm.

bioRxiv preprint doi: https://doi.org/10.1101/2023.04.19.537522; this version posted April 21, 2023. The copyright holder for this preprint (which was not certified by peer review) is the author/funder, who has granted bioRxiv a license to display the preprint in perpetuity. It is made available under aCC-BY 4.0 International license.

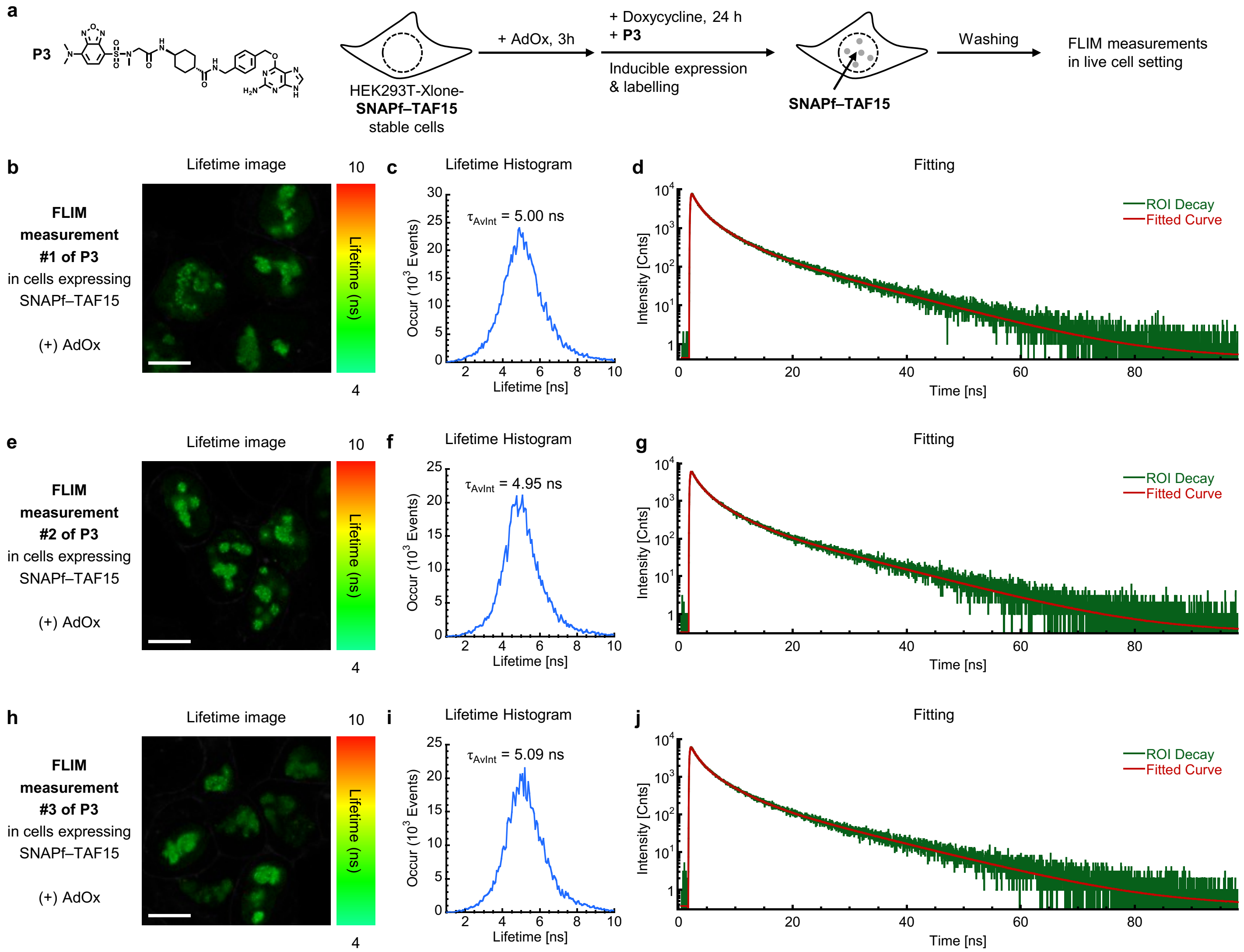

Figure S29. FLIM measurements of P3 with live cells stably expressing SNAPf–TAF15 with AdOx pretreatment. (**a**) The stable cells were pretreated with AdOx (25 µM) 3 hours before they were further treated with doxycycline (140 ng/mL) and **P3** (0.5 µM) for 24 hours for the inducible expression and labeling of SNAPf–TAF15, respectively. After the cells were washed with fresh fluorobrite^{TM} DMEM media supplemented with fetal bovine serum (10%) to remove excess probes, the lifetime of **P3** was measured using Zeiss LSM 880 microscope with a PicoQuant-FLIM LSM upgrade KIT. (**b**), (**e**), (**h**) lifetime images from three individual measurements. (**c**), (**f**), (**i**) lifetime histograms from the images b, e, and h, respectively. (**d**), (**g**), (**j**) lifetime decay fitting from the images b, e, and h, respectively. Scale bar = 10 µm.

bioRxiv preprint doi: https://doi.org/10.1101/2023.04.19.537522; this version posted April 21, 2023. The copyright holder for this preprint (which was not certified by peer review) is the author/funder, who has granted bioRxiv a license to display the preprint in perpetuity. It is made available under aCC-BY 4.0 International license.

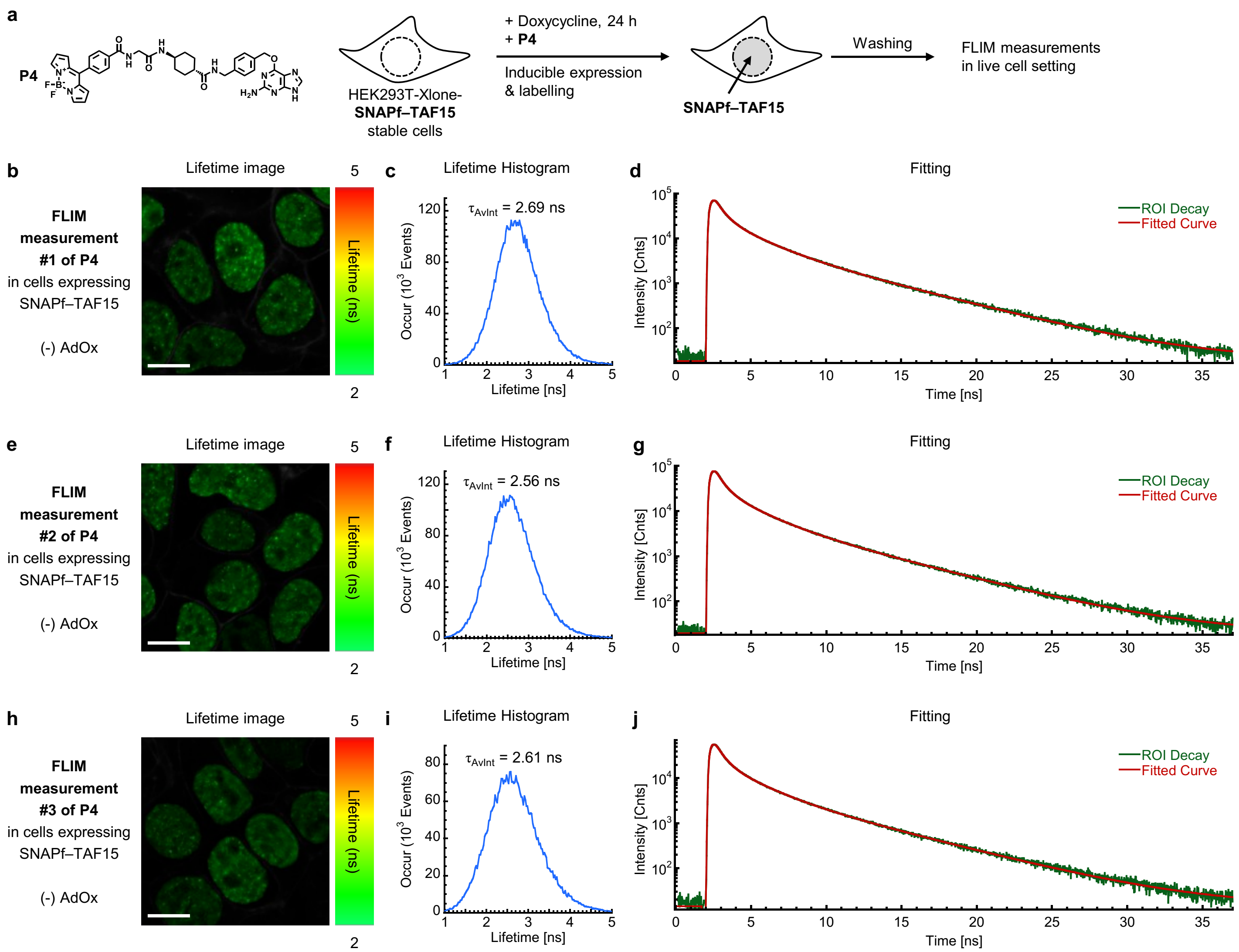

Figure S30. FLIM measurements of P4 with live cells stably expressing SNAPf–TAF15 without AdOx pretreatment. (**a**) The stable cells were treated with doxycycline (140 ng/mL) and **P4** (0.5 µM) for 24 hours for the inducible expression and labeling of SNAPf–TAF15, respectively. After the cells were washed with fresh fluorobrite™ DMEM media supplemented with fetal bovine serum (10%) to remove excess probes, the lifetime of **P4** was measured using Zeiss LSM 880 microscope with a PicoQuant-FLIM LSM upgrade KIT. (**b**), (**e**), (**h**) lifetime images from three individual measurements. (**c**), (**f**), (**i**) lifetime histograms from the images b, e, and h, respectively. (**d**), (**g**), (**j**) lifetime decay fitting from the images b, e, and h, respectively. Scale bar = 10 µm.

bioRxiv preprint doi: https://doi.org/10.1101/2023.04.19.537522; this version posted April 21, 2023. The copyright holder for this preprint (which was not certified by peer review) is the author/funder, who has granted bioRxiv a license to display the preprint in perpetuity. It is made available under aCC-BY 4.0 International license.

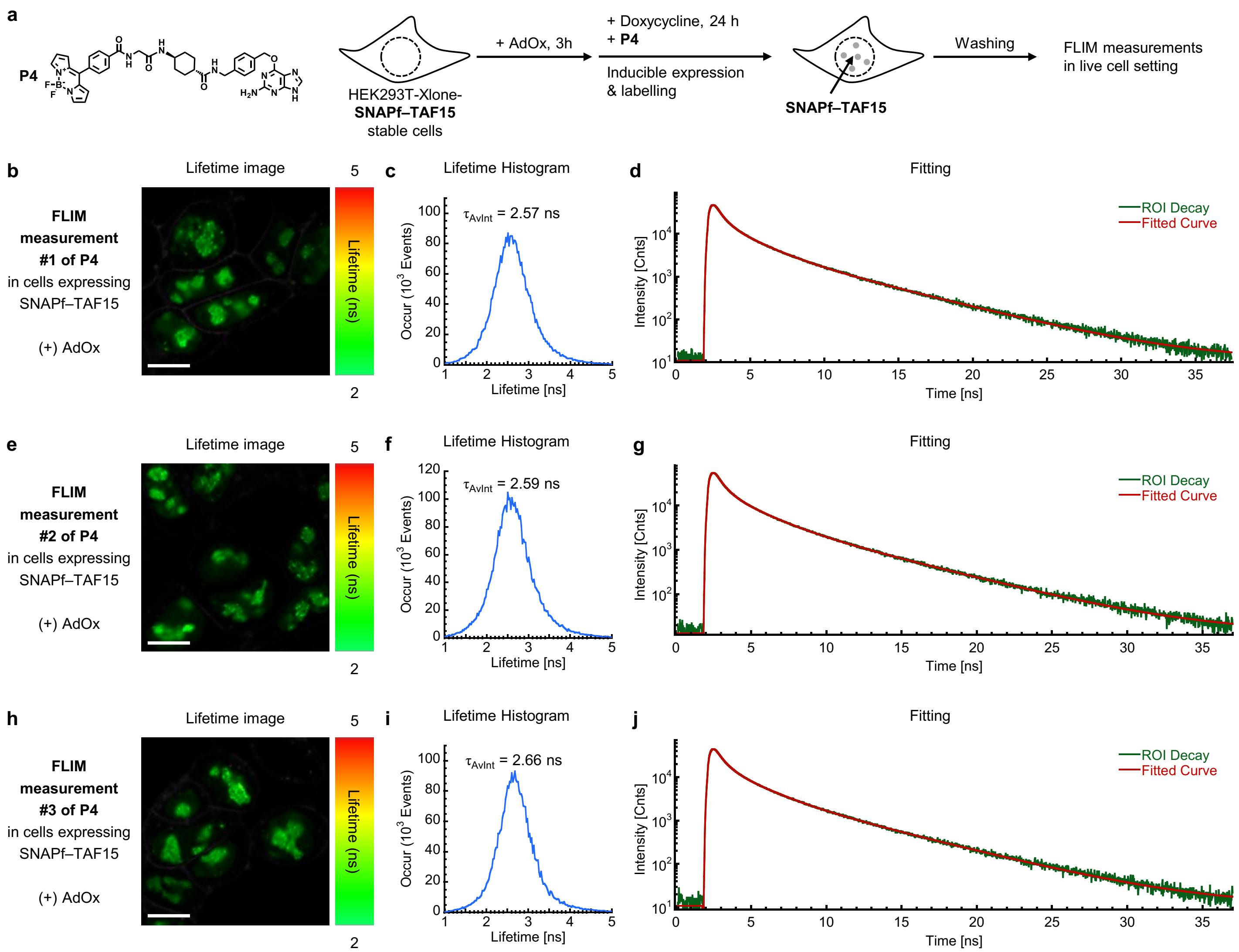

Figure S31. FLIM measurements of P4 with live cells stably expressing SNAPf–TAF15 with AdOx pretreatment. (**a**) The stable cells were pretreated with AdOx (25 μM) 3 hours before they were further treated with doxycycline (140 ng/mL) and **P4** (0.5 μM) for 24 hours for the inducible expression and labeling of SNAPf–TAF15, respectively. After the cells were washed with fresh fluorobrite™ DMEM media supplemented with fetal bovine serum (10%) to remove excess probes, the lifetime of **P4** was measured using Zeiss LSM 880 microscope with a PicoQuant-FLIM LSM upgrade KIT. (**b**), (**e**), (**h**) lifetime images from three individual measurements. (**c**), (**f**), (**i**) lifetime histograms from the images b, e, and h, respectively. (**d**), (**g**), (**j**) lifetime decay fitting from the images b, e, and h, respectively. Scale bar = 10 μm.

bioRxiv preprint doi: https://doi.org/10.1101/2023.04.19.537522; this version posted April 21, 2023. The copyright holder for this preprint (which was not certified by peer review) is the author/funder, who has granted bioRxiv a license to display the preprint in perpetuity. It is made available under aCC-BY 4.0 International license.

NMR Characterizations

^{1}H NMR of **P0** (DMSO-d_6, 500 MHz)

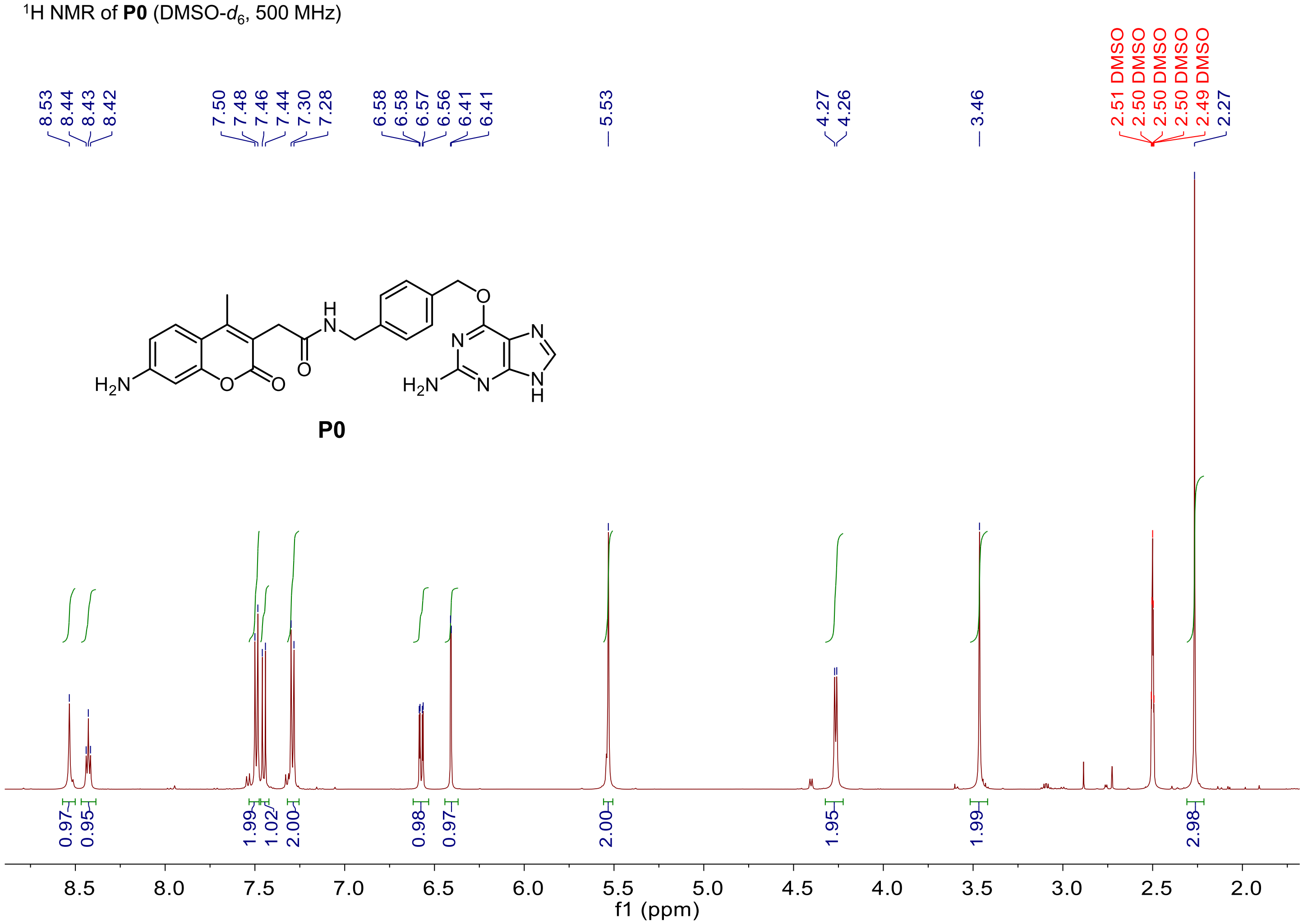

bioRxiv preprint doi: https://doi.org/10.1101/2023.04.19.537522; this version posted April 21, 2023. The copyright holder for this preprint (which was not certified by peer review) is the author/funder, who has granted bioRxiv a license to display the preprint in perpetuity. It is made available under aCC-BY 4.0 International license.

^{13}C NMR of **P0** (DMSO-d_6, 126 MHz)

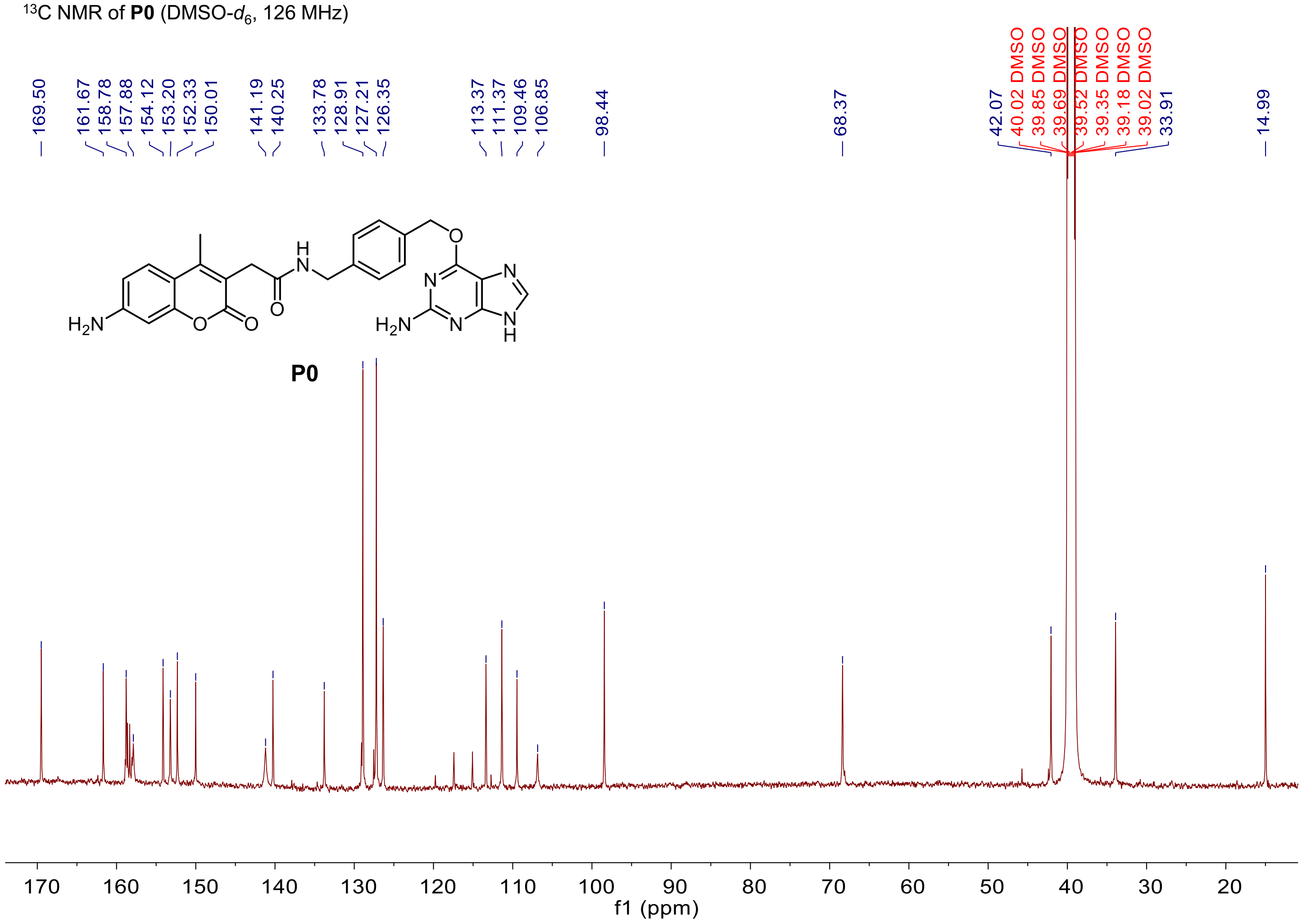

bioRxiv preprint doi: https://doi.org/10.1101/2023.04.19.537522; this version posted April 21, 2023. The copyright holder for this preprint (which was not certified by peer review) is the author/funder, who has granted bioRxiv a license to display the preprint in perpetuity. It is made available under aCC-BY 4.0 International license.

^{1}H NMR of **1** (DMSO-d_6, 500 MHz)

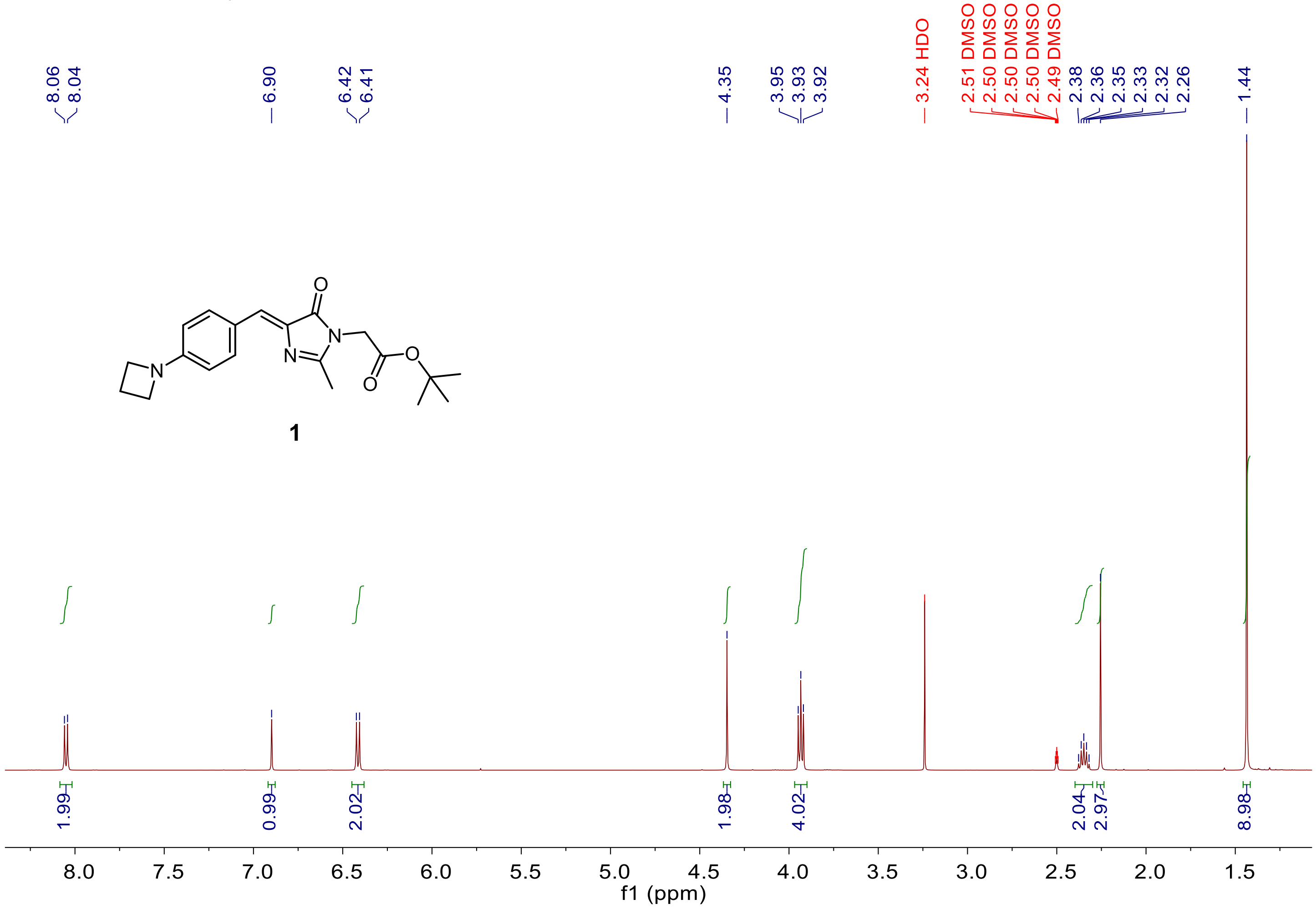

bioRxiv preprint doi: https://doi.org/10.1101/2023.04.19.537522; this version posted April 21, 2023. The copyright holder for this preprint (which was not certified by peer review) is the author/funder, who has granted bioRxiv a license to display the preprint in perpetuity. It is made available under aCC-BY 4.0 International license.

^{13}C NMR of **1** (DMSO-d_6, 126 MHz)

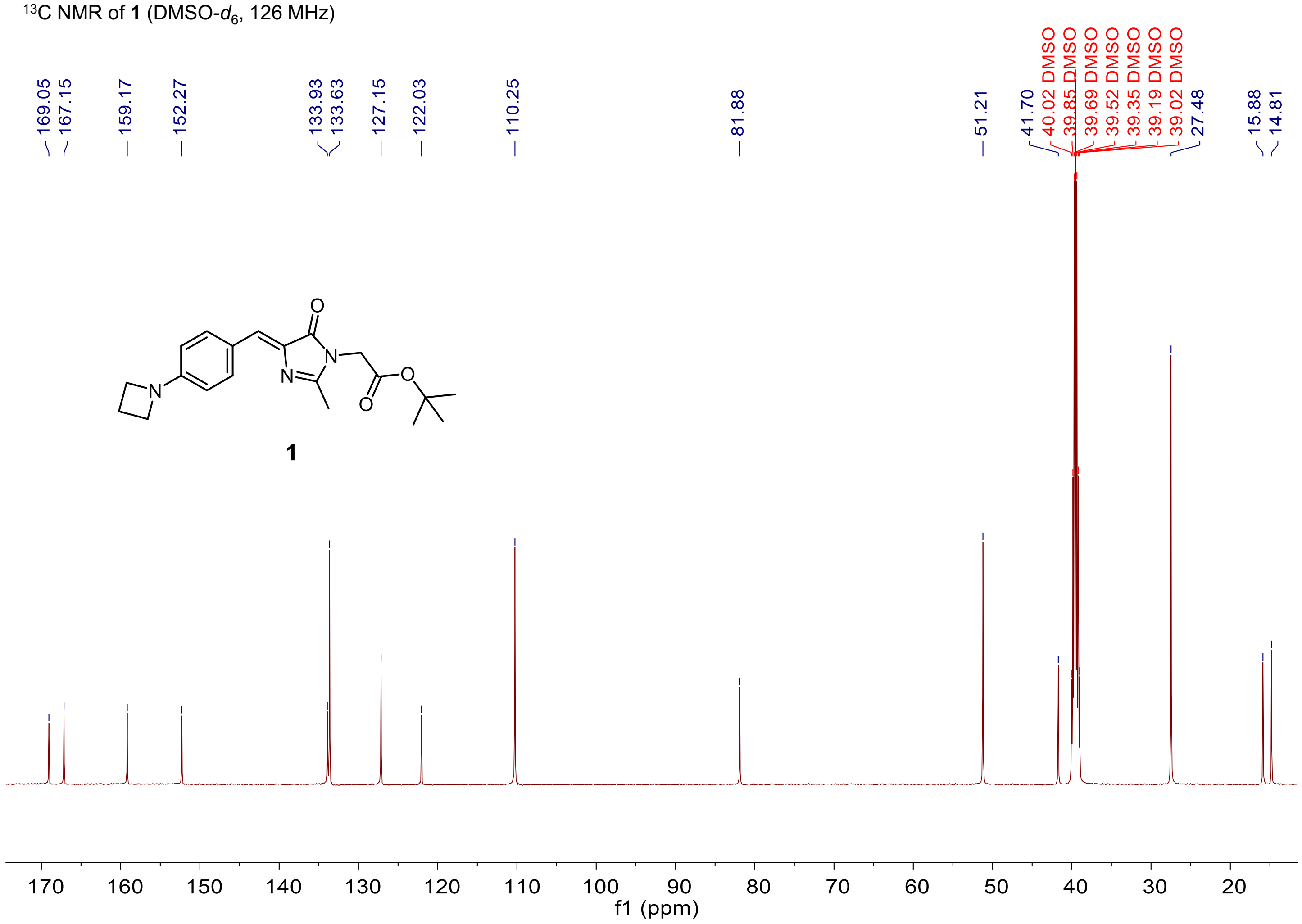

bioRxiv preprint doi: https://doi.org/10.1101/2023.04.19.537522; this version posted April 21, 2023. The copyright holder for this preprint (which was not certified by peer review) is the author/funder, who has granted bioRxiv a license to display the preprint in perpetuity. It is made available under aCC-BY 4.0 International license.

^{1}H NMR of **2** ($CDCl_3$, 500 MHz)

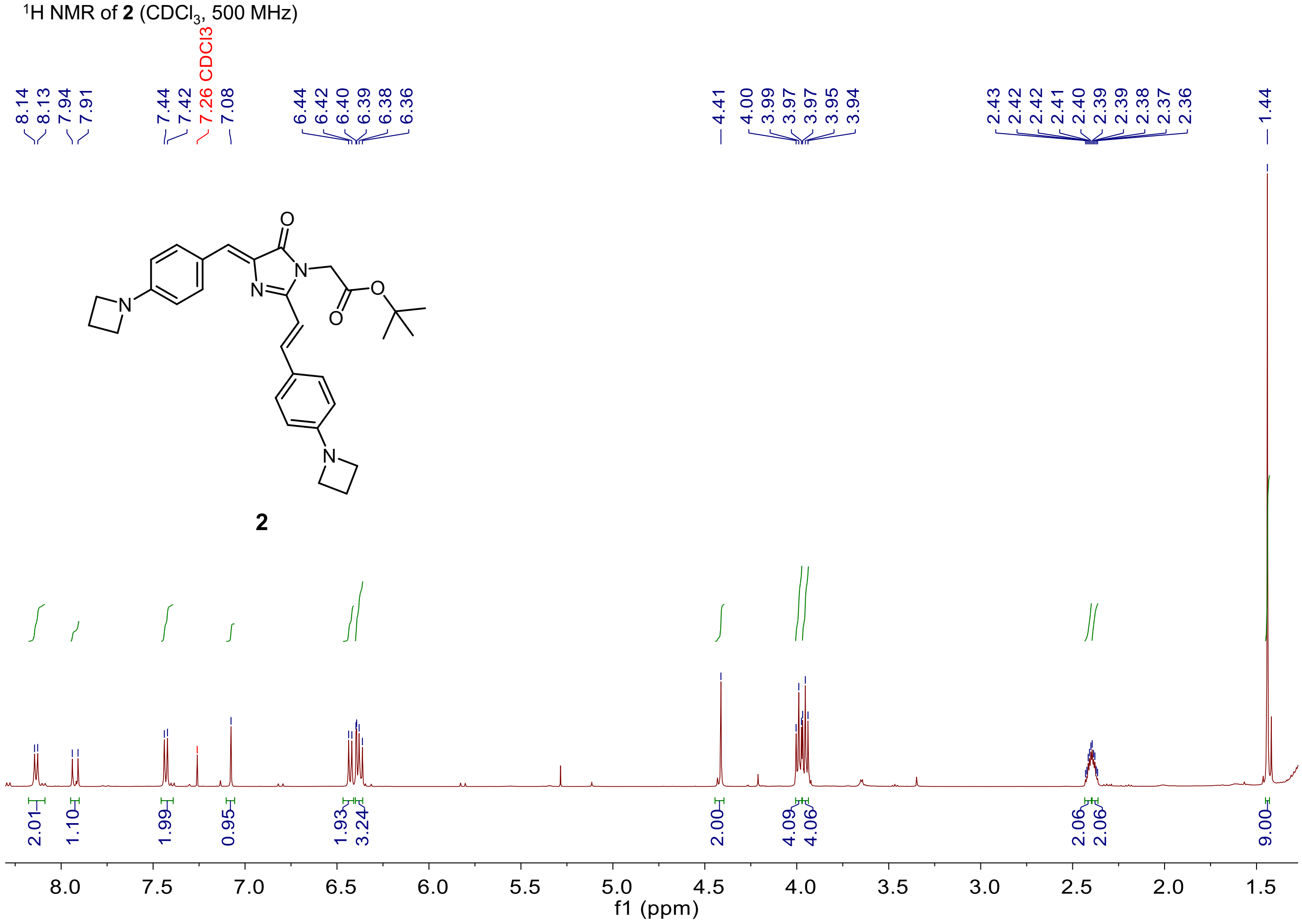

bioRxiv preprint doi: https://doi.org/10.1101/2023.04.19.537522; this version posted April 21, 2023. The copyright holder for this preprint (which was not certified by peer review) is the author/funder, who has granted bioRxiv a license to display the preprint in perpetuity. It is made available under aCC-BY 4.0 International license.

^{13}C NMR of **2** ($CDCl_3$, 126 MHz)

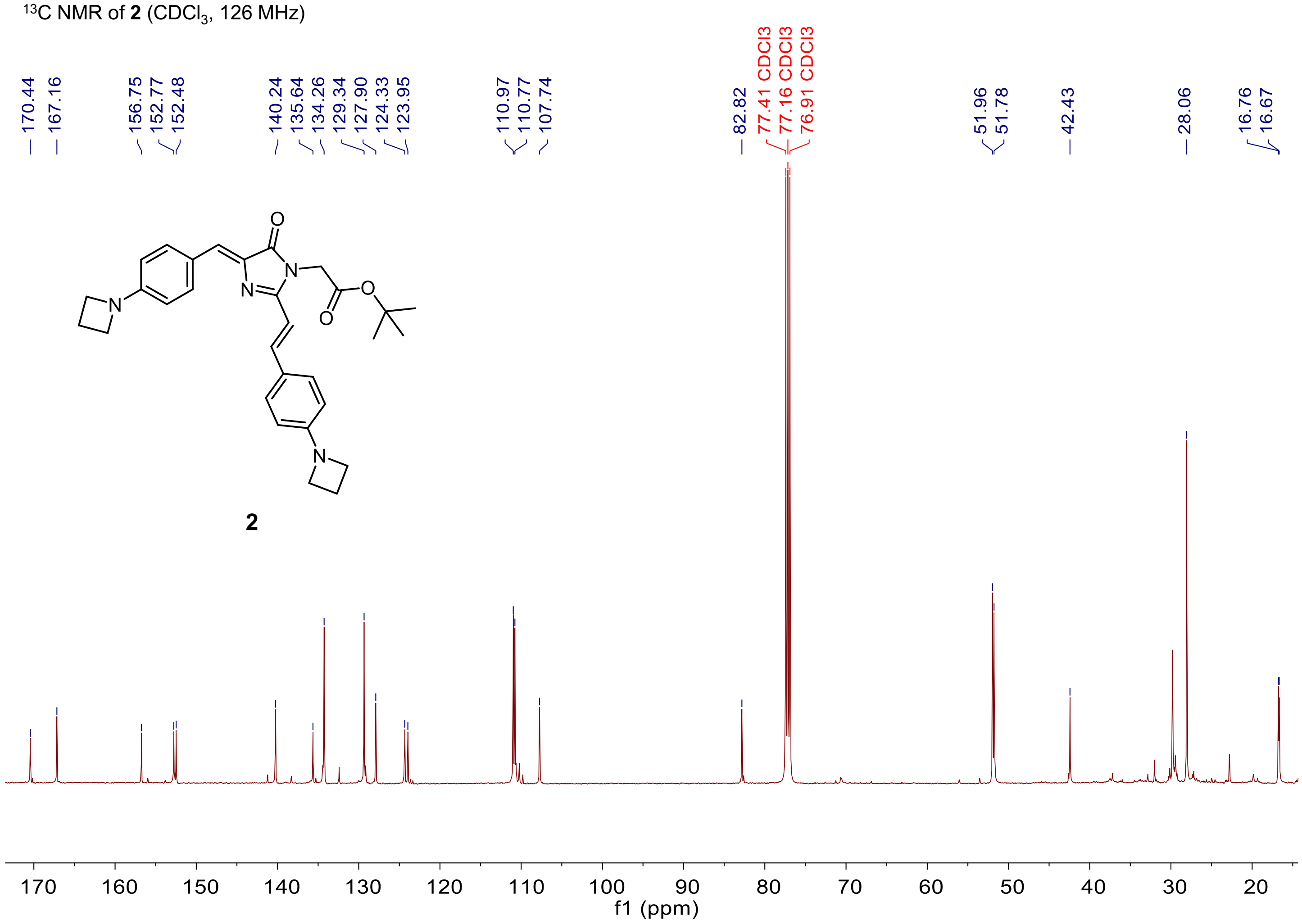

bioRxiv preprint doi: https://doi.org/10.1101/2023.04.19.537522; this version posted April 21, 2023. The copyright holder for this preprint (which was not certified by peer review) is the author/funder, who has granted bioRxiv a license to display the preprint in perpetuity. It is made available under aCC-BY 4.0 International license.

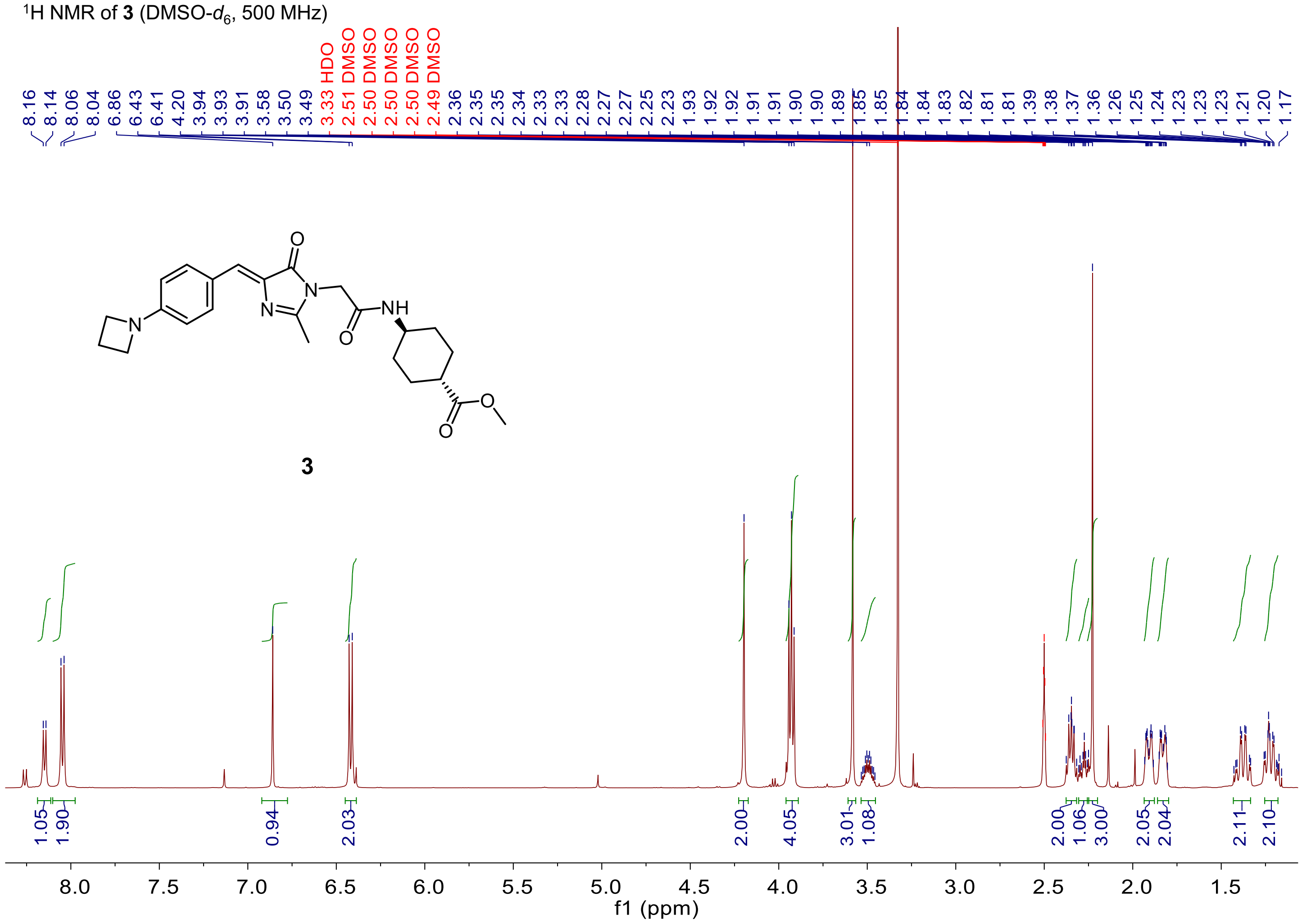

3

bioRxiv preprint doi: https://doi.org/10.1101/2023.04.19.537522; this version posted April 21, 2023. The copyright holder for this preprint (which was not certified by peer review) is the author/funder, who has granted bioRxiv a license to display the preprint in perpetuity. It is made available under aCC-BY 4.0 International license.

^{13}C NMR of **3** (DMSO-d_6, 126 MHz)

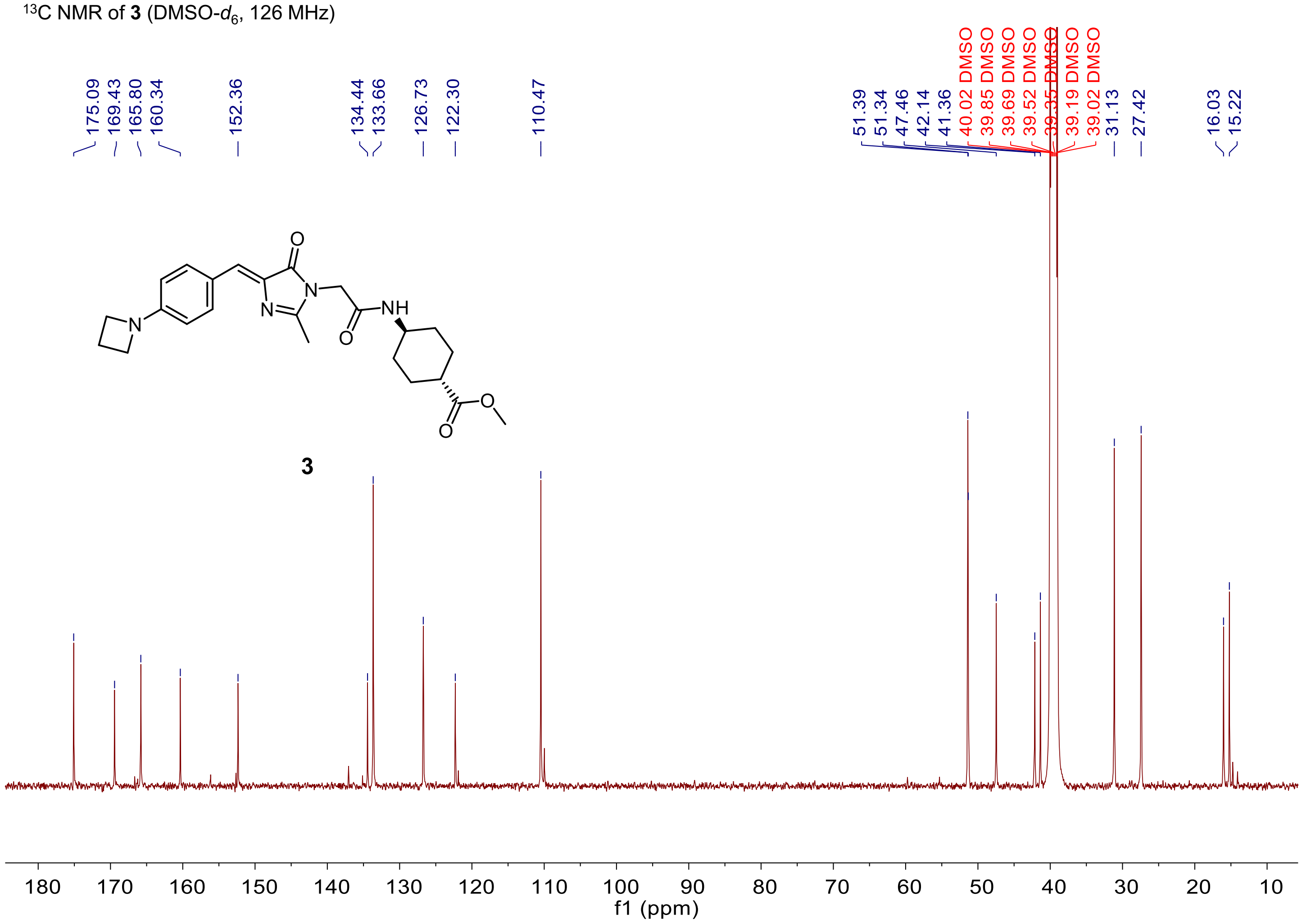

bioRxiv preprint doi: https://doi.org/10.1101/2023.04.19.537522; this version posted April 21, 2023. The copyright holder for this preprint (which was not certified by peer review) is the author/funder, who has granted bioRxiv a license to display the preprint in perpetuity. It is made available under aCC-BY 4.0 International license.

^{1}H NMR of **4** ($CDCl_3$, 500 MHz)

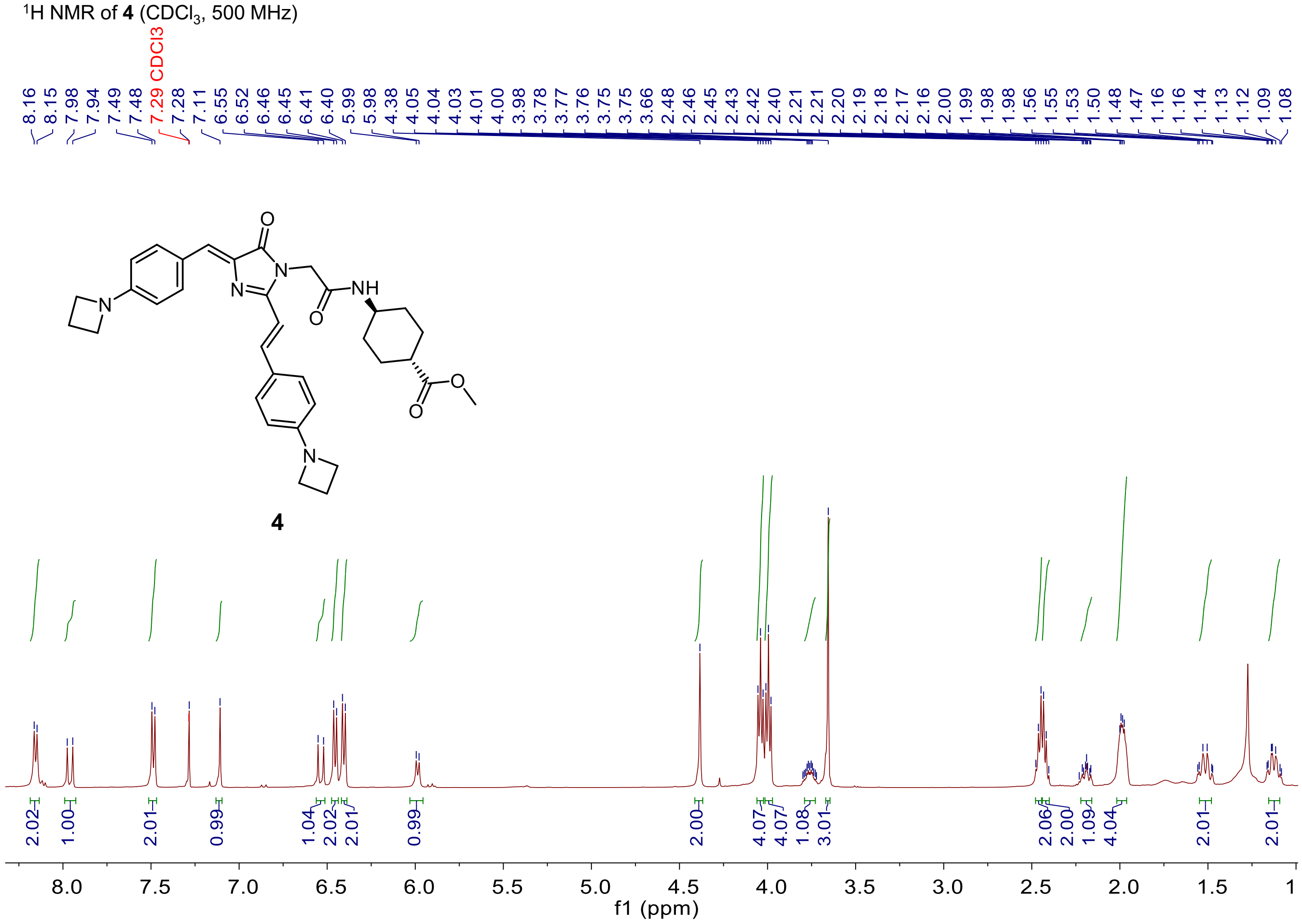

bioRxiv preprint doi: https://doi.org/10.1101/2023.04.19.537522; this version posted April 21, 2023. The copyright holder for this preprint (which was not certified by peer review) is the author/funder, who has granted bioRxiv a license to display the preprint in perpetuity. It is made available under aCC-BY 4.0 International license.

^{13}C NMR of **4** ($CDCl_3$, 126 MHz)

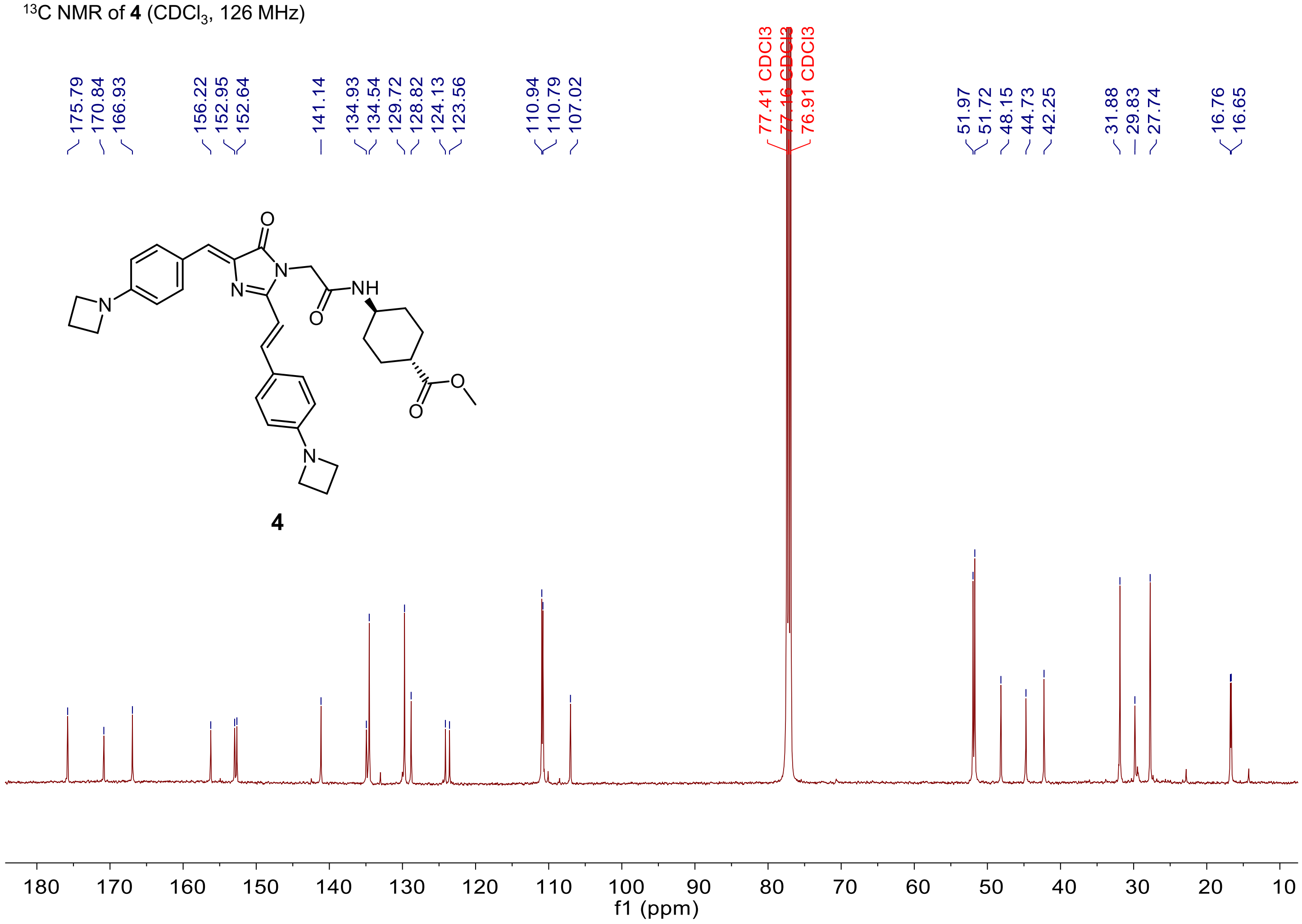

bioRxiv preprint doi: https://doi.org/10.1101/2023.04.19.537522; this version posted April 21, 2023. The copyright holder for this preprint (which was not certified by peer review) is the author/funder, who has granted bioRxiv a license to display the preprint in perpetuity. It is made available under aCC-BY 4.0 International license.

^{1}H NMR of **P2** (DMSO-d_6, 500 MHz)

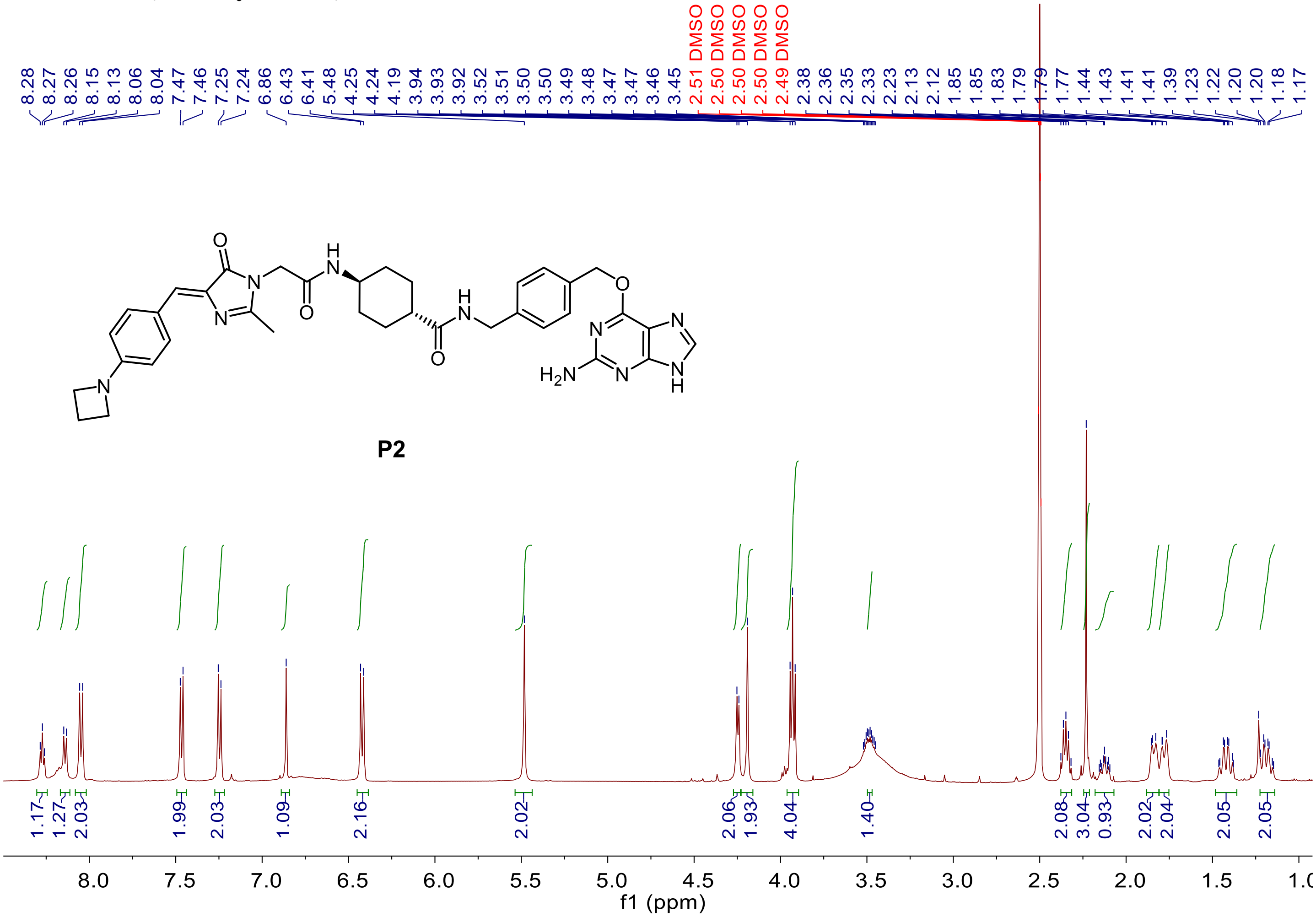

bioRxiv preprint doi: https://doi.org/10.1101/2023.04.19.537522; this version posted April 21, 2023. The copyright holder for this preprint (which was not certified by peer review) is the author/funder, who has granted bioRxiv a license to display the preprint in perpetuity. It is made available under aCC-BY 4.0 International license.

^{13}C NMR of **P2** (DMSO-d_6, 126 MHz)

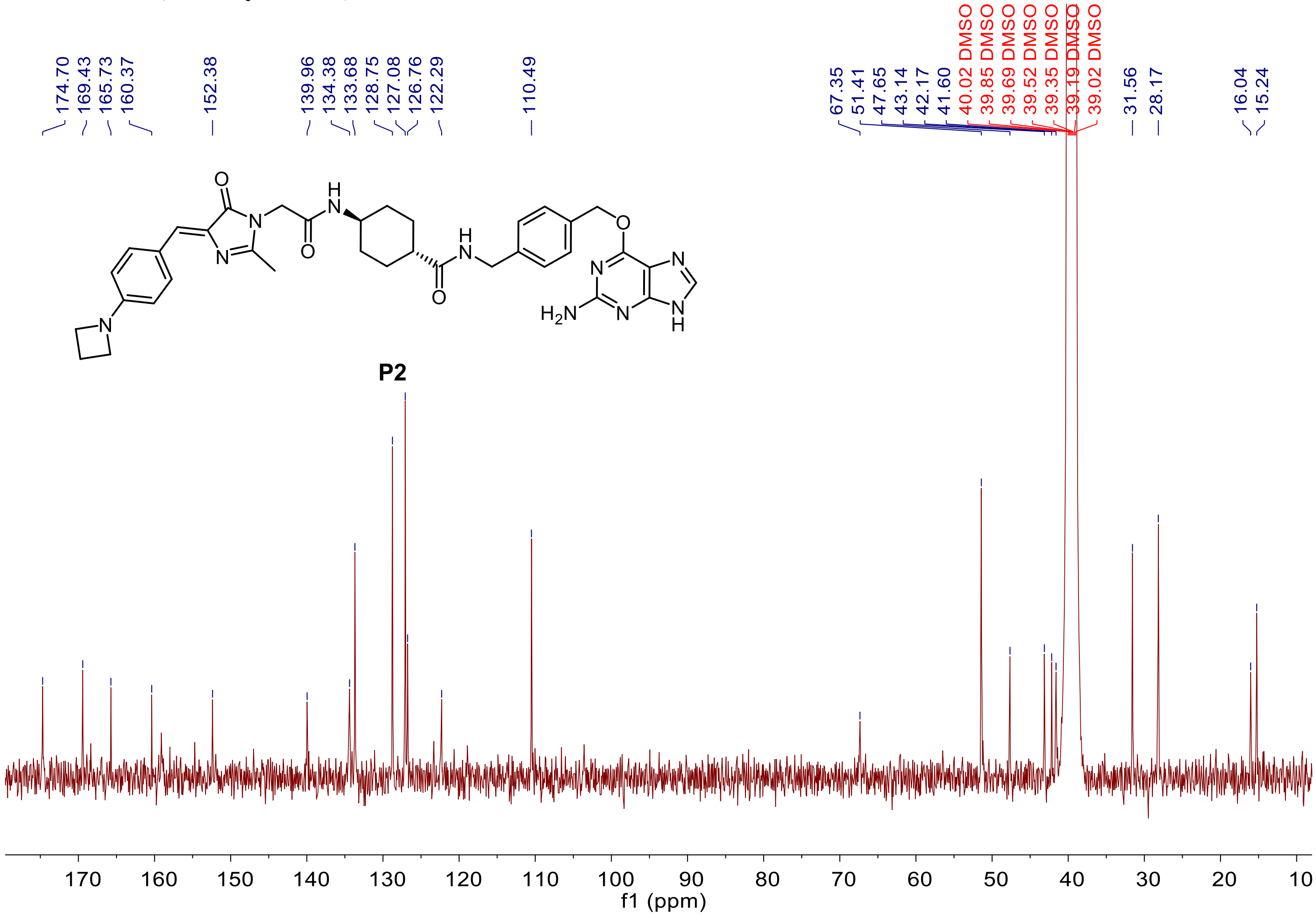

bioRxiv preprint doi: https://doi.org/10.1101/2023.04.19.537522; this version posted April 21, 2023. The copyright holder for this preprint (which was not certified by peer review) is the author/funder, who has granted bioRxiv a license to display the preprint in perpetuity. It is made available under aCC-BY 4.0 International license.

^{1}H NMR of **P1** (DMSO-d_6, 500 MHz)

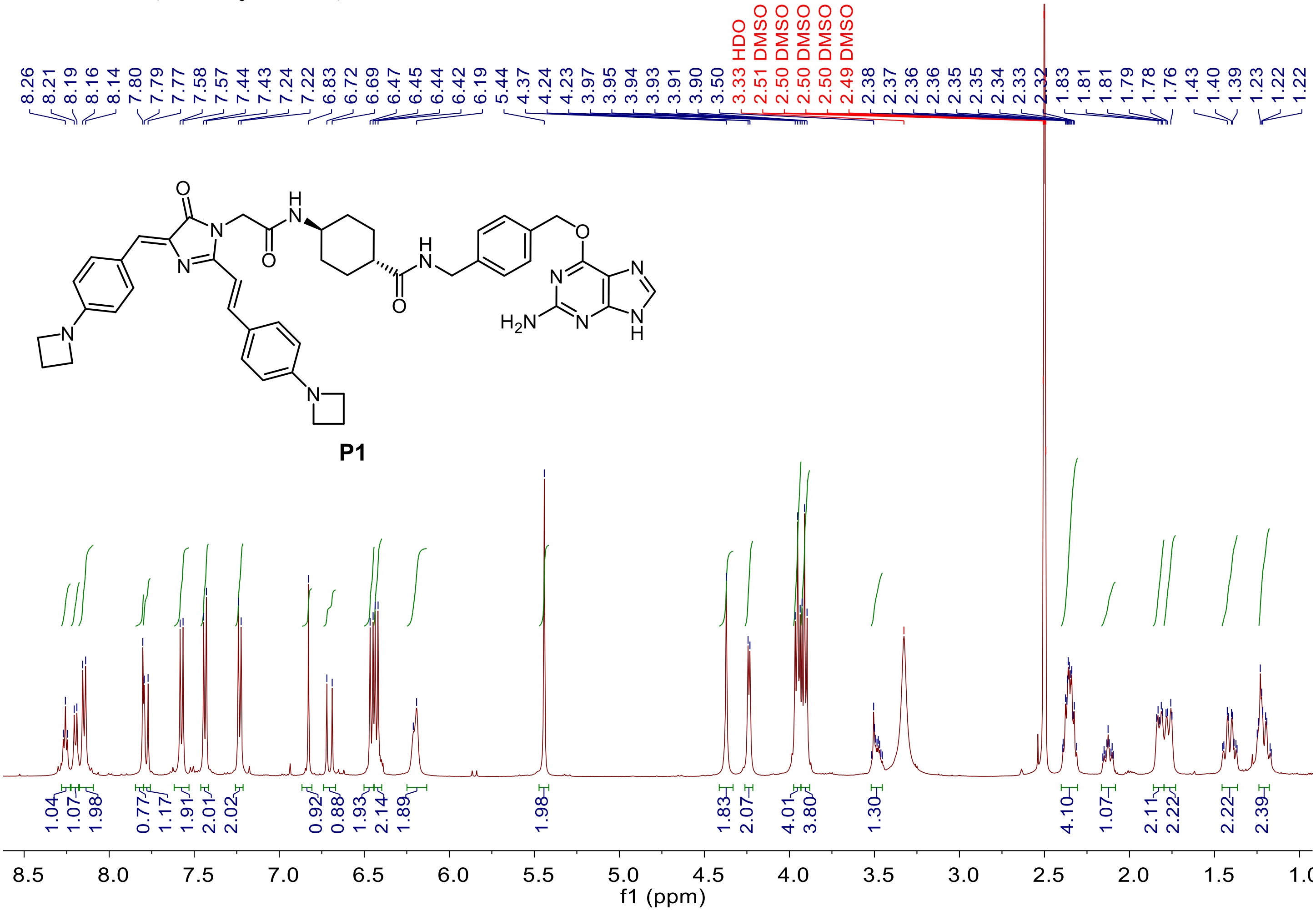

bioRxiv preprint doi: https://doi.org/10.1101/2023.04.19.537522; this version posted April 21, 2023. The copyright holder for this preprint (which was not certified by peer review) is the author/funder, who has granted bioRxiv a license to display the preprint in perpetuity. It is made available under aCC-BY 4.0 International license.

^{13}C NMR of **P1** (DMSO-d_6, 126 MHz)

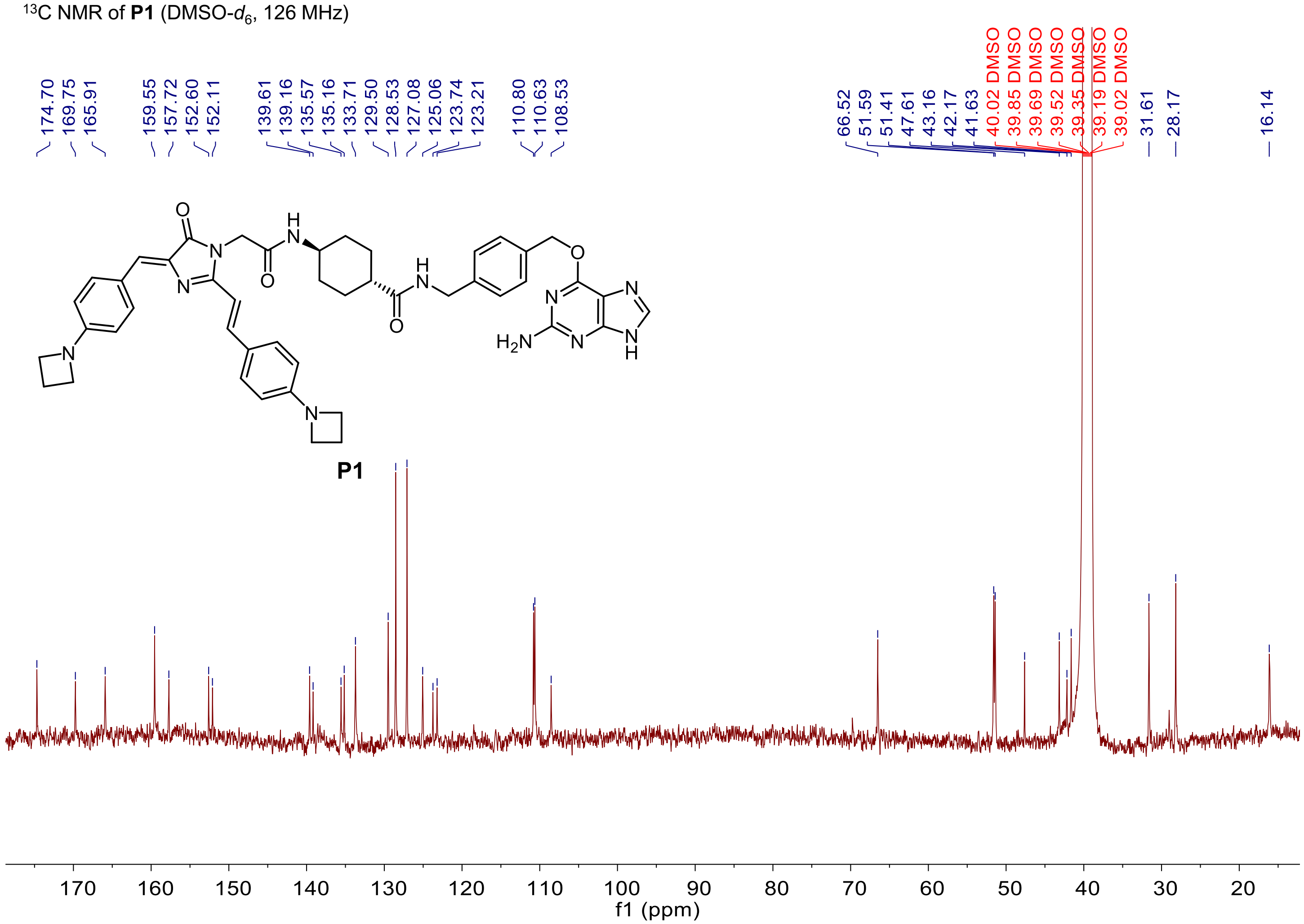

bioRxiv preprint doi: https://doi.org/10.1101/2023.04.19.537522; this version posted April 21, 2023. The copyright holder for this preprint (which was not certified by peer review) is the author/funder, who has granted bioRxiv a license to display the preprint in perpetuity. It is made available under aCC-BY 4.0 International license.

^{1}H NMR of **6** (DMSO-d_6, 500 MHz)

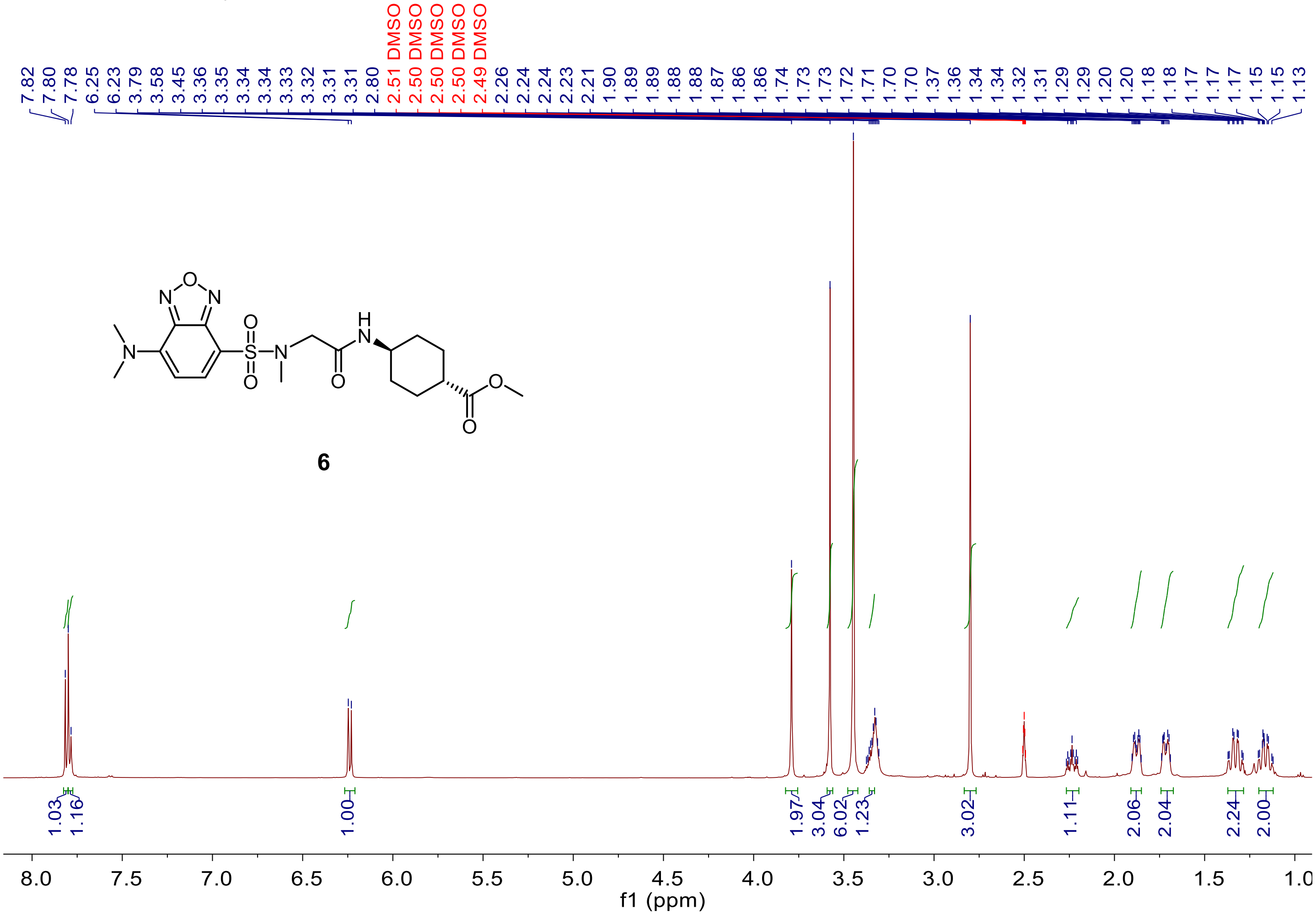

bioRxiv preprint doi: https://doi.org/10.1101/2023.04.19.537522; this version posted April 21, 2023. The copyright holder for this preprint (which was not certified by peer review) is the author/funder, who has granted bioRxiv a license to display the preprint in perpetuity. It is made available under aCC-BY 4.0 International license.

^{13}C NMR of **6** (DMSO-d_6, 126 MHz)

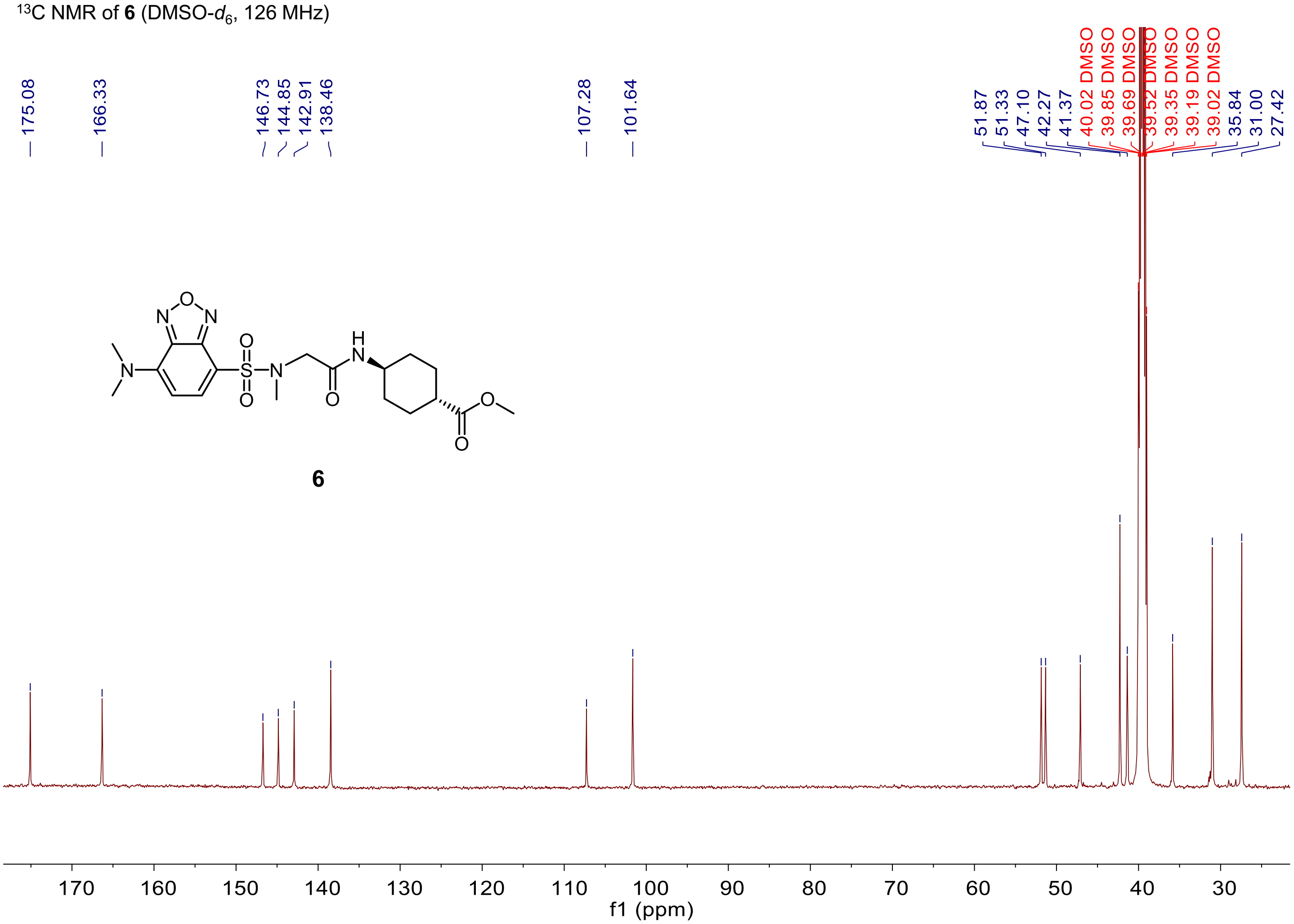

bioRxiv preprint doi: https://doi.org/10.1101/2023.04.19.537522; this version posted April 21, 2023. The copyright holder for this preprint (which was not certified by peer review) is the author/funder, who has granted bioRxiv a license to display the preprint in perpetuity. It is made available under aCC-BY 4.0 International license.

^{1}H NMR of **P3** (DMSO-d_6, 500 MHz)

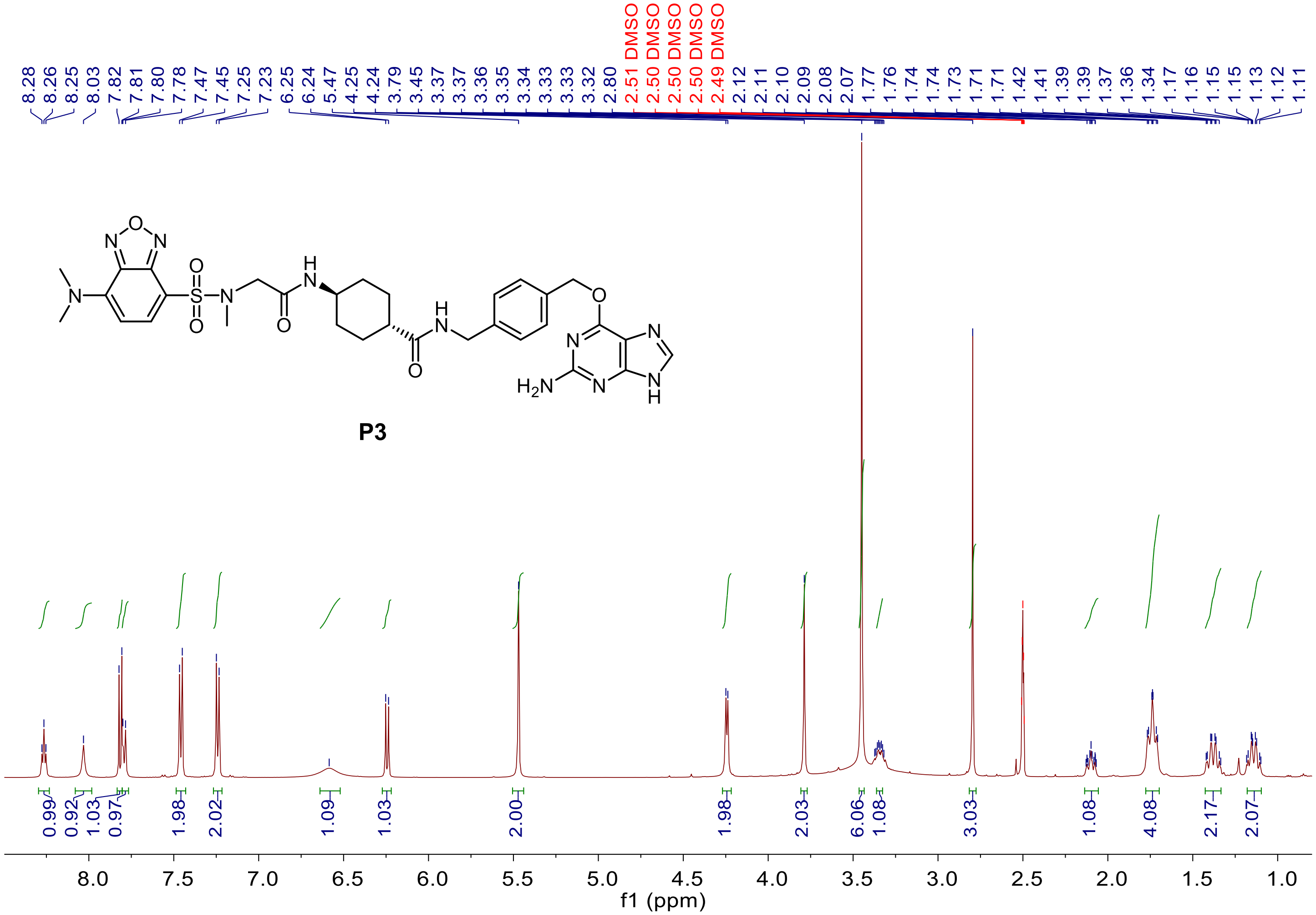

bioRxiv preprint doi: https://doi.org/10.1101/2023.04.19.537522; this version posted April 21, 2023. The copyright holder for this preprint (which was not certified by peer review) is the author/funder, who has granted bioRxiv a license to display the preprint in perpetuity. It is made available under aCC-BY 4.0 International license.

^{13}C NMR of **P3** (DMSO-d_6, 126 MHz)

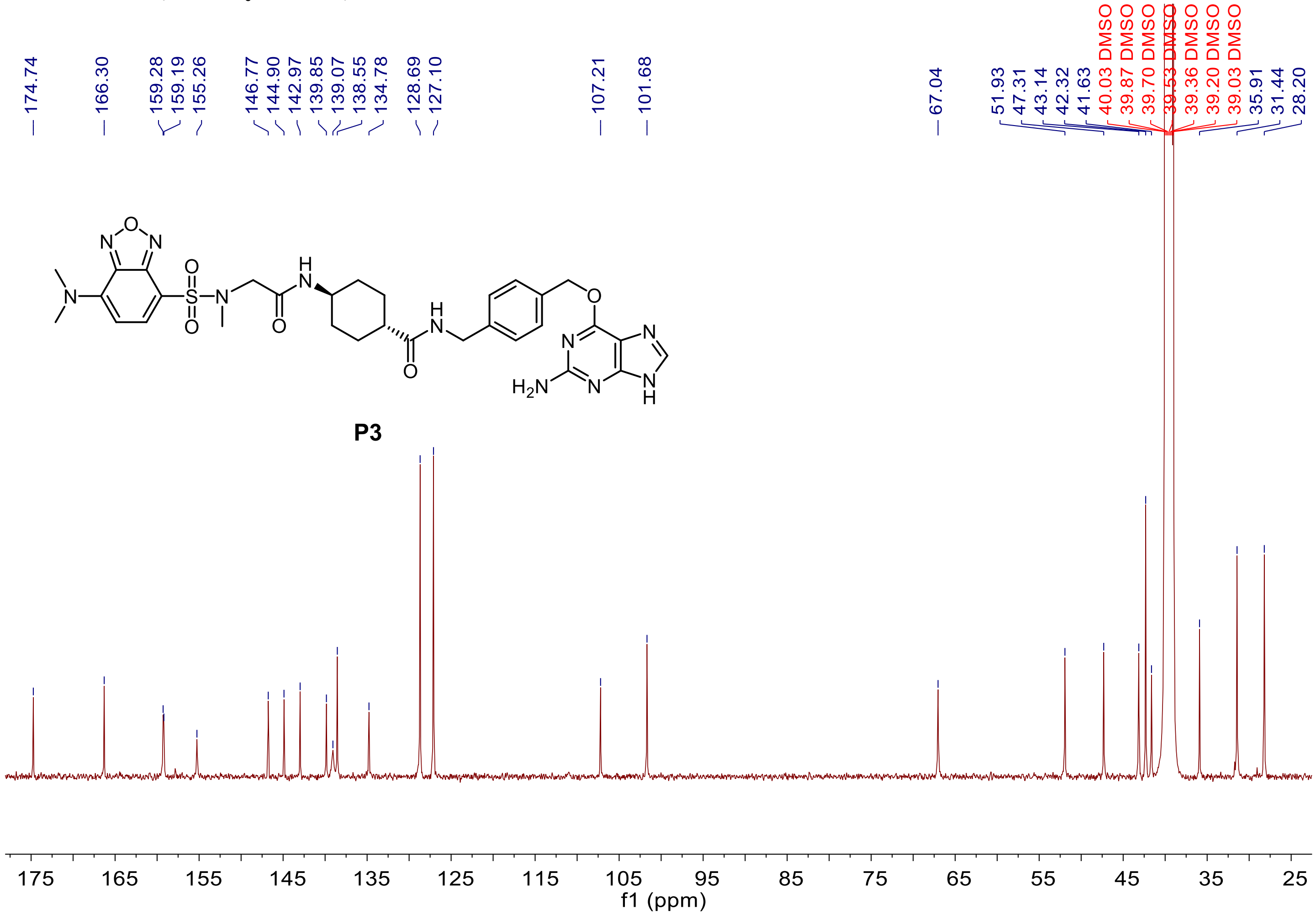

bioRxiv preprint doi: https://doi.org/10.1101/2023.04.19.537522; this version posted April 21, 2023. The copyright holder for this preprint (which was not certified by peer review) is the author/funder, who has granted bioRxiv a license to display the preprint in perpetuity. It is made available under aCC-BY 4.0 International license.

^{1}H NMR of **8** (DMSO-d_6, 500 MHz)

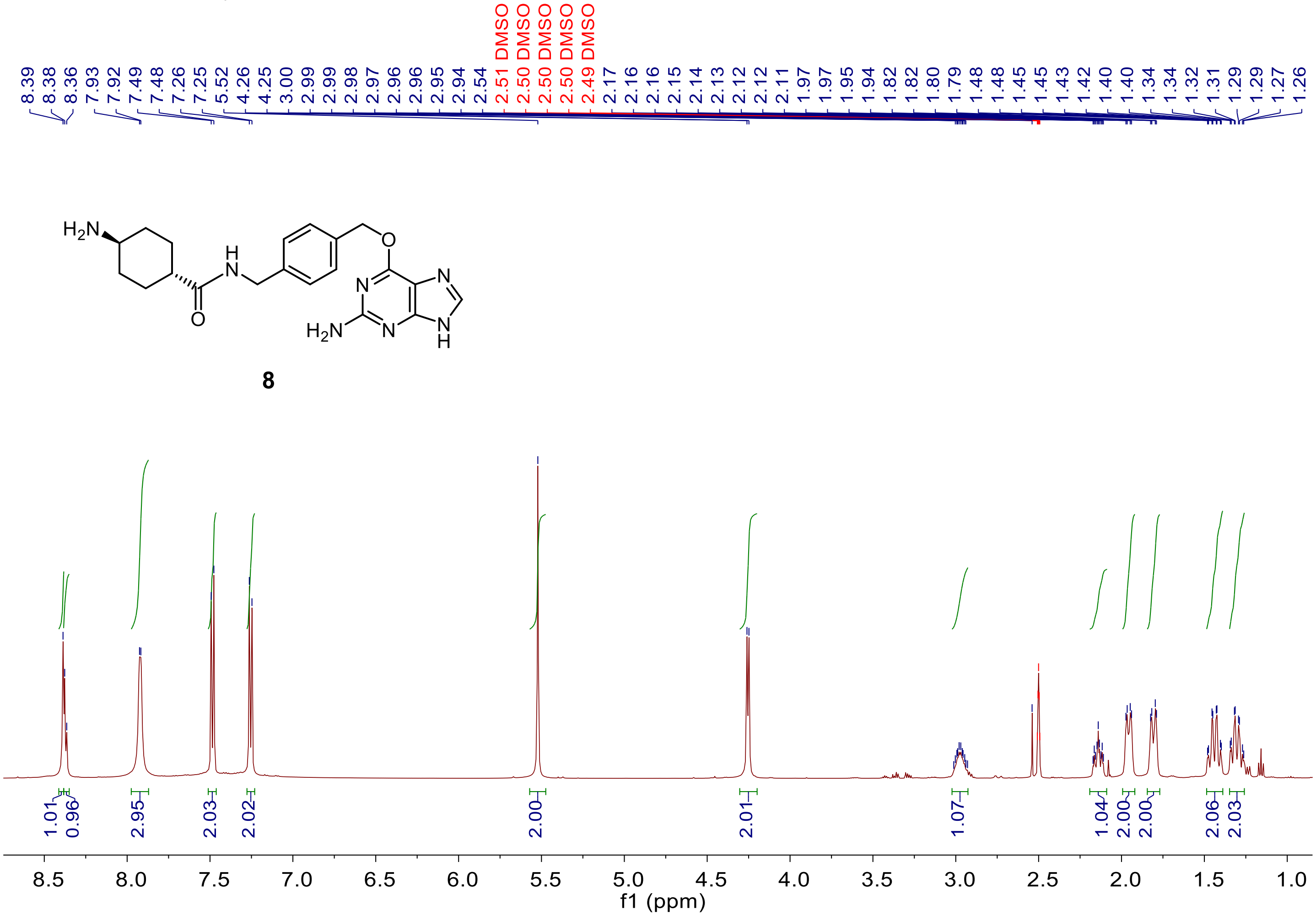

8

bioRxiv preprint doi: https://doi.org/10.1101/2023.04.19.537522; this version posted April 21, 2023. The copyright holder for this preprint (which was not certified by peer review) is the author/funder, who has granted bioRxiv a license to display the preprint in perpetuity. It is made available under aCC-BY 4.0 International license.

^{13}C NMR of **8** (DMSO-d_6, 126 MHz)

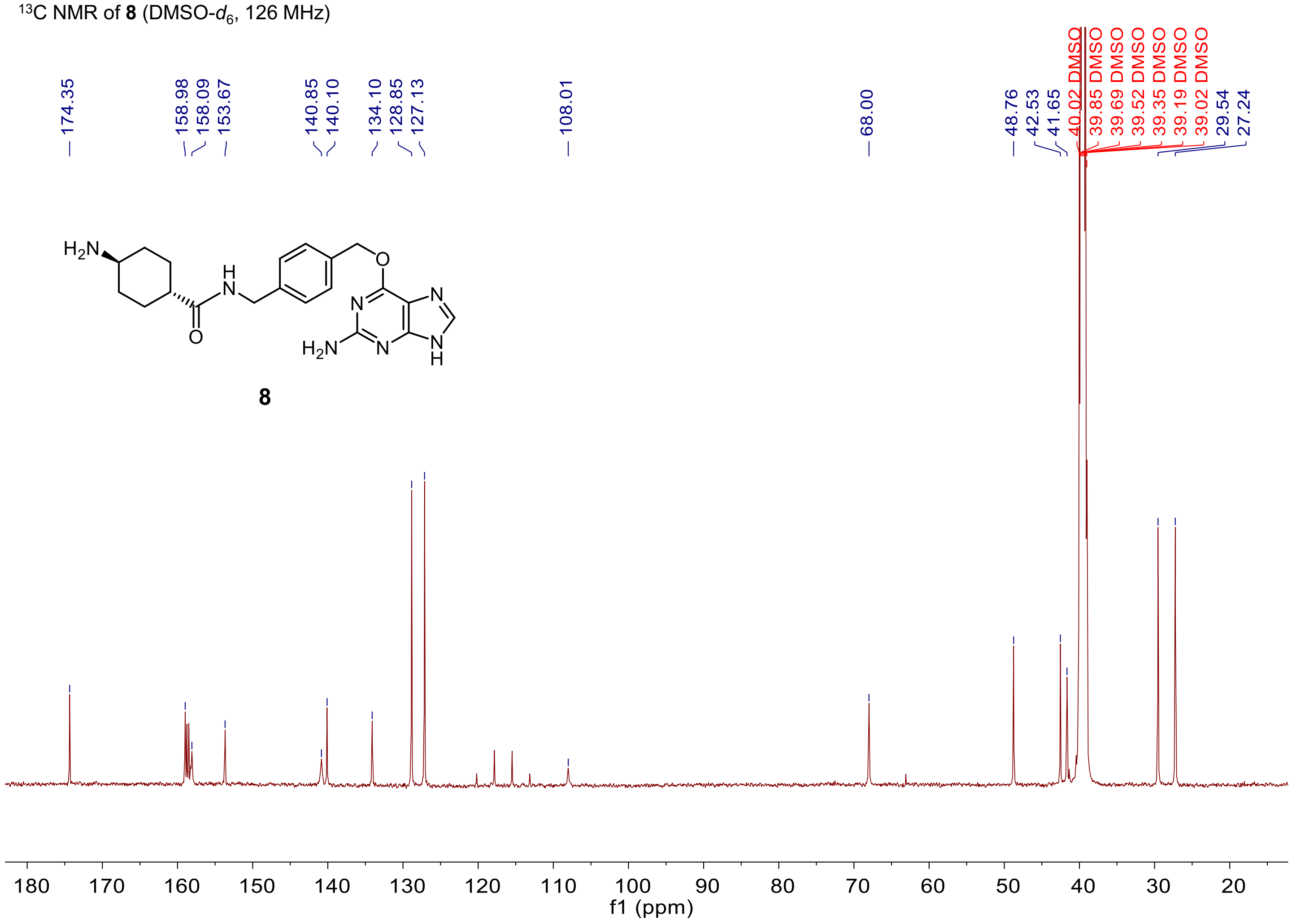

bioRxiv preprint doi: https://doi.org/10.1101/2023.04.19.537522; this version posted April 21, 2023. The copyright holder for this preprint (which was not certified by peer review) is the author/funder, who has granted bioRxiv a license to display the preprint in perpetuity. It is made available under aCC-BY 4.0 International license.

^{1}H NMR of **9** (DMSO-d_6, 500 MHz)

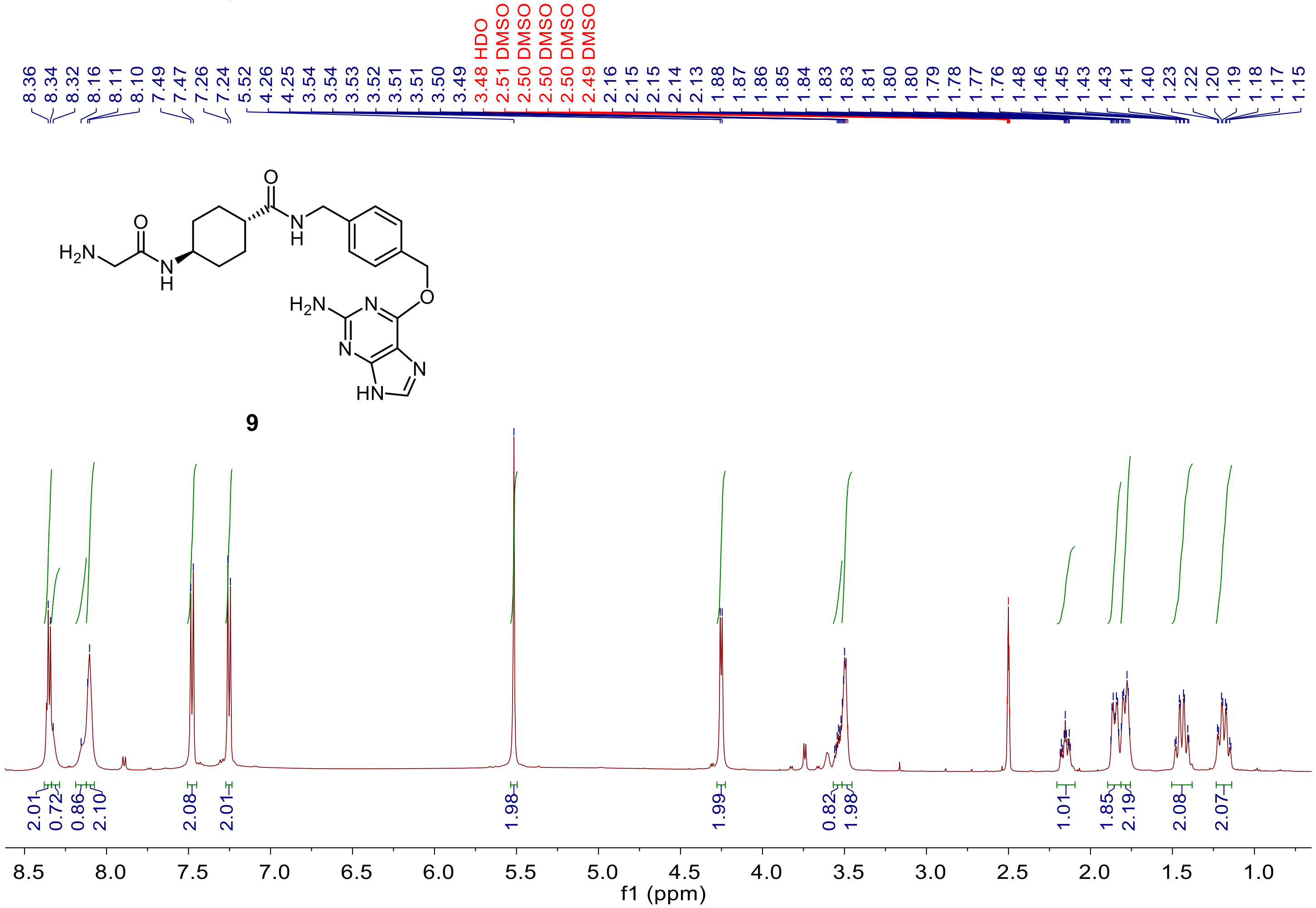

bioRxiv preprint doi: https://doi.org/10.1101/2023.04.19.537522; this version posted April 21, 2023. The copyright holder for this preprint (which was not certified by peer review) is the author/funder, who has granted bioRxiv a license to display the preprint in perpetuity. It is made available under aCC-BY 4.0 International license.

^{13}C NMR of **9** (DMSO-d_6, 126 MHz)

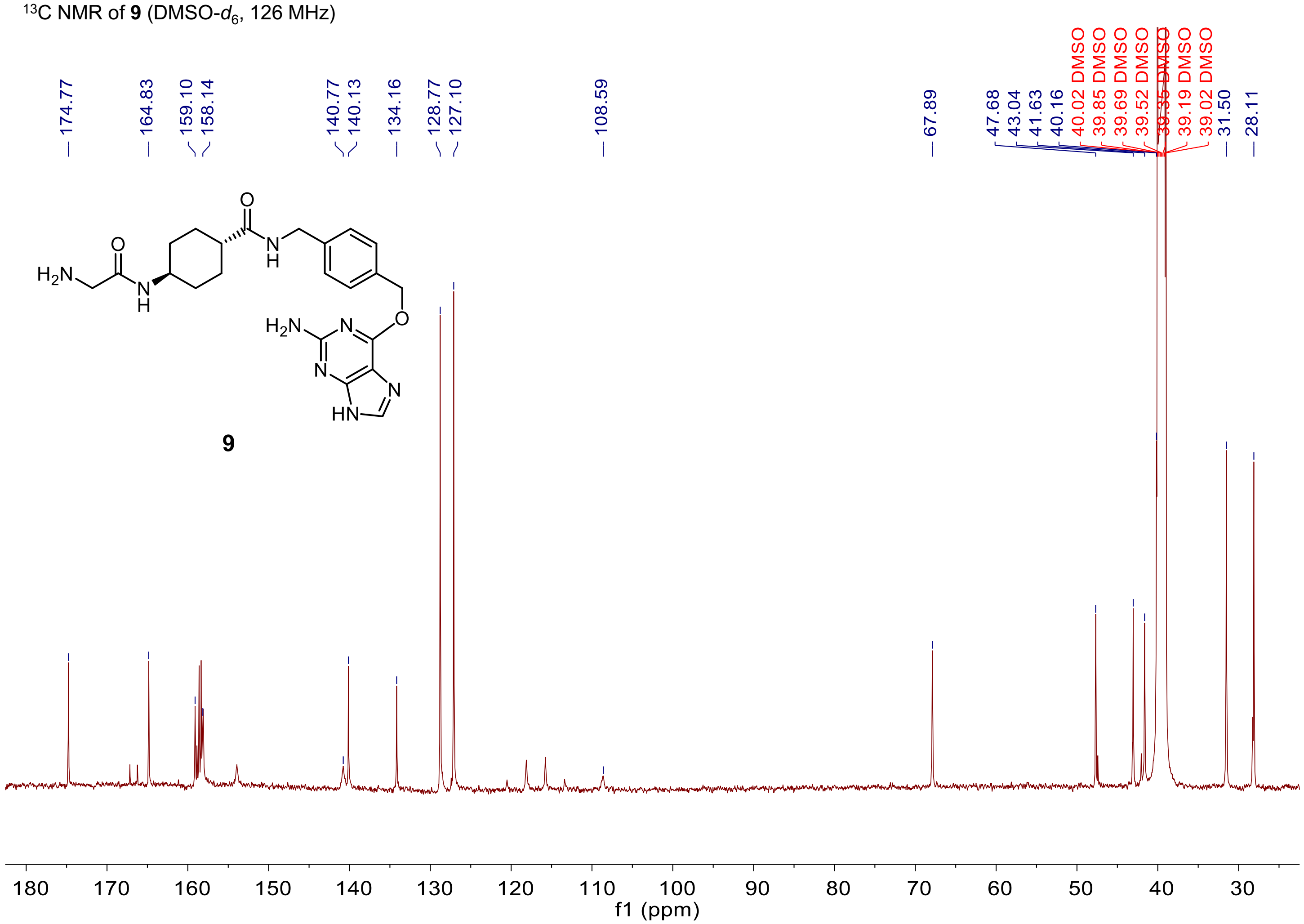

bioRxiv preprint doi: https://doi.org/10.1101/2023.04.19.537522; this version posted April 21, 2023. The copyright holder for this preprint (which was not certified by peer review) is the author/funder, who has granted bioRxiv a license to display the preprint in perpetuity. It is made available under aCC-BY 4.0 International license.

^{1}H NMR of **P4** (DMSO-d_6, 500 MHz)

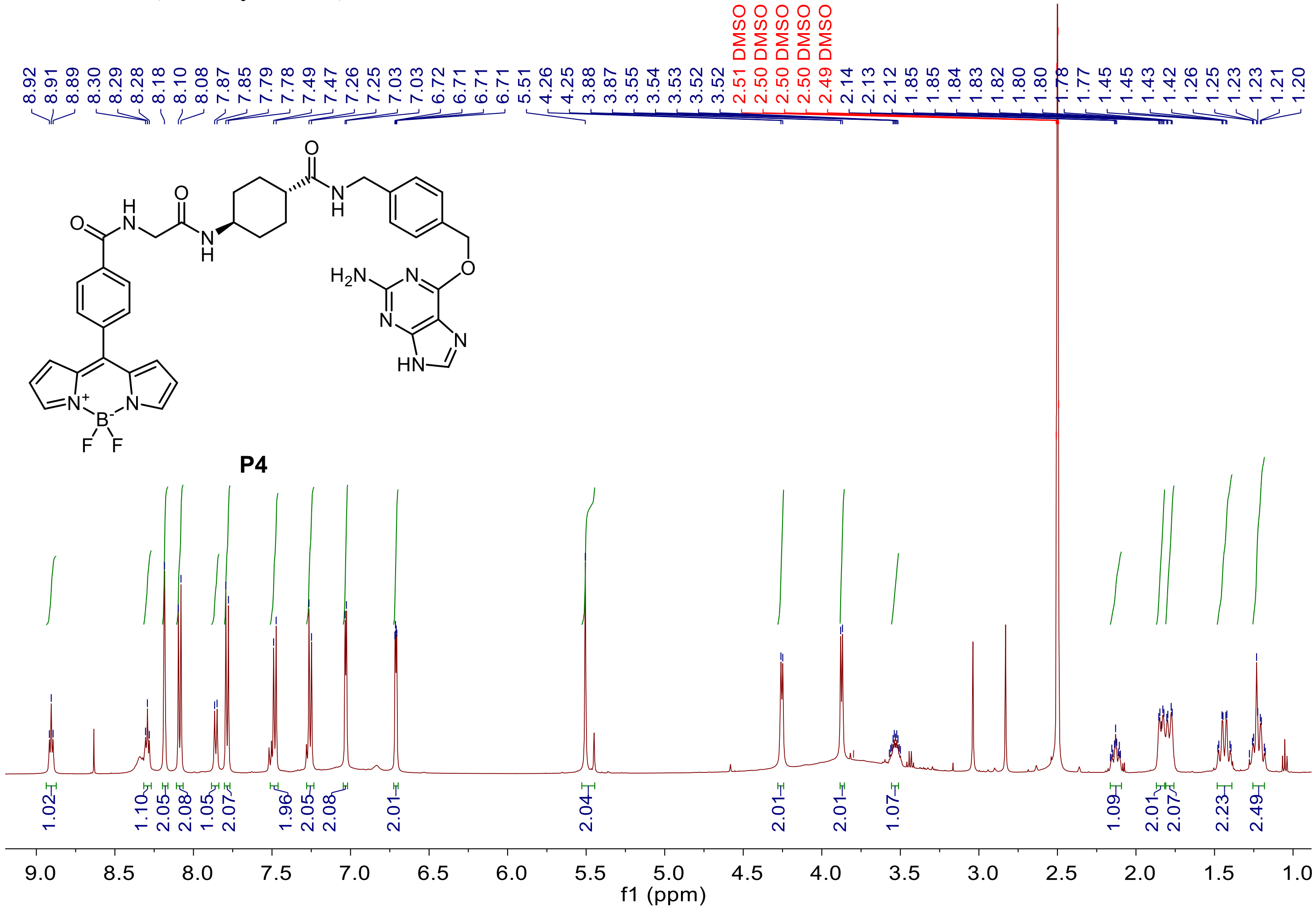

bioRxiv preprint doi: https://doi.org/10.1101/2023.04.19.537522; this version posted April 21, 2023. The copyright holder for this preprint (which was not certified by peer review) is the author/funder, who has granted bioRxiv a license to display the preprint in perpetuity. It is made available under aCC-BY 4.0 International license.

^{13}C NMR of **P4** (DMSO-d_6, 126 MHz)

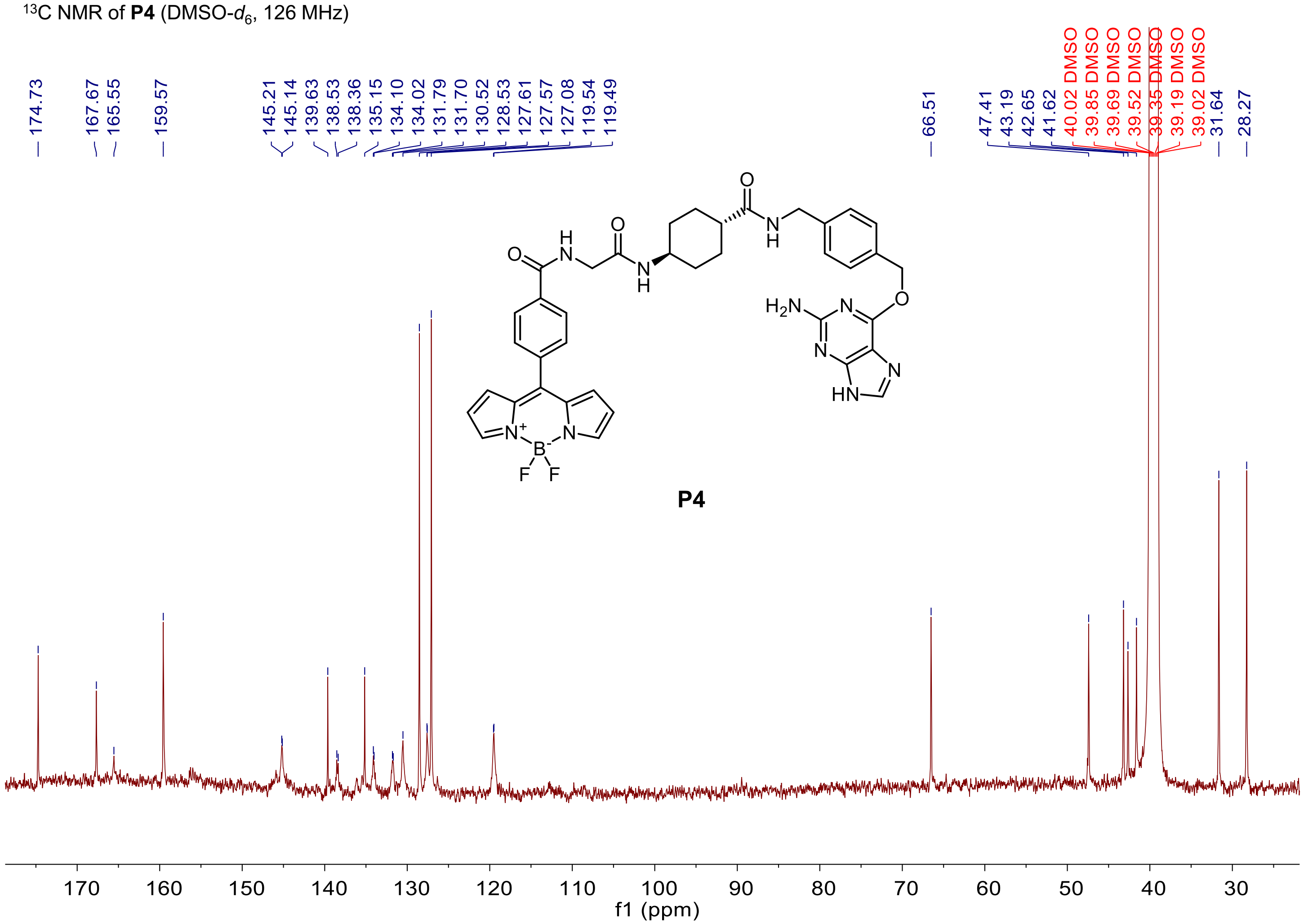

bioRxiv preprint doi: https://doi.org/10.1101/2023.04.19.537522; this version posted April 21, 2023. The copyright holder for this preprint (which was not certified by peer review) is the author/funder, who has granted bioRxiv a license to display the preprint in perpetuity. It is made available under aCC-BY 4.0 International license.

^{19}F NMR of **P4** (DMSO-d_6, 471 MHz)

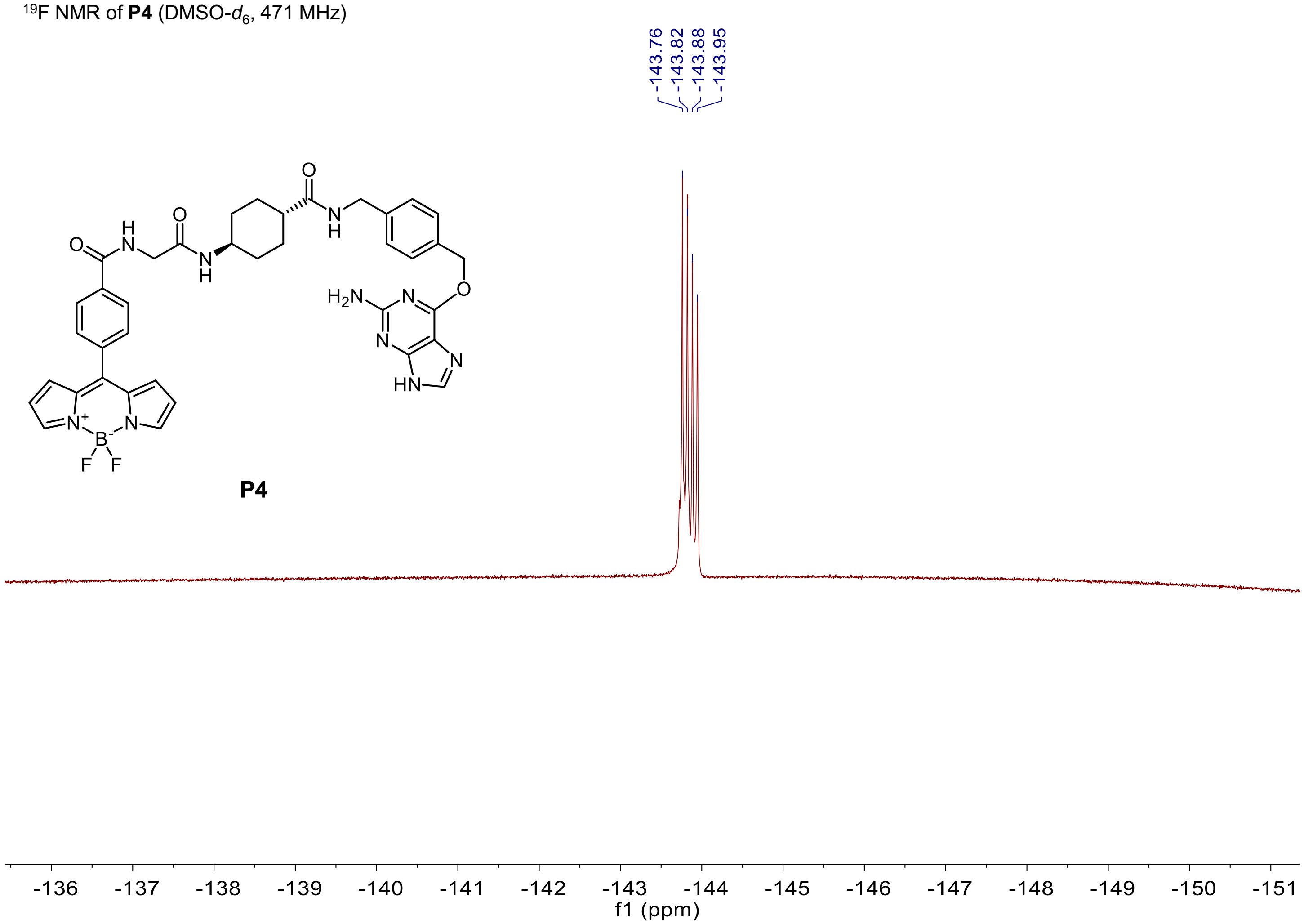

Supplement: Supplement 1 [file NIHPP2023.04.19.537522v1-supplement-1.pdf]
